# *SALL3* mediates the loss of neuroectodermal differentiation potential in human embryonic stem cells with chromosome 18q loss

**DOI:** 10.1016/j.stemcr.2024.03.001

**Published:** 2024-03-28

**Authors:** Yingnan Lei, Diana Al Delbany, Nuša Krivec, Marius Regin, Edouard Couvreu de Deckersberg, Charlotte Janssens, Manjusha Ghosh, Karen Sermon, Claudia Spits

**Affiliations:** 1 Research Group Reproduction and Genetics, Faculty of Medicine and Pharmacy, Vrije Universiteit Brussel, Brussels, Laarbeeklaan 103, 1090 Brussels, Belgium

**Keywords:** SALL3, 18q loss, differentiation bias, neuroectoderm, human embryonic stem cells, human pluripotent stem cells

## Abstract

Human pluripotent stem cell (hPSC) cultures are prone to genetic drift, because cells that have acquired specific genetic abnormalities experience a selective advantage *in vitro*. These abnormalities are highly recurrent in hPSC lines worldwide, but their functional consequences in differentiating cells are scarcely described. In this work, we show that the loss of chromosome 18q impairs neuroectoderm commitment and that downregulation of *SALL3*, a gene located in the common 18q loss region, is responsible for this failed neuroectodermal differentiation. Knockdown of *SALL3* in control lines impaired differentiation in a manner similar to the loss of 18q, and transgenic overexpression of *SALL3* in hESCs with 18q loss rescued the differentiation capacity of the cells. Finally, we show that loss of 18q and downregulation of *SALL3* leads to changes in the expression of genes involved in pathways regulating pluripotency and differentiation, suggesting that these cells are in an altered state of pluripotency.

## Highlights

• hPSC cultures worldwide recurrently acquire losses of chromosome 18q
• Loss of 18q results in impaired neuroectodermal commitment
• Downregulation of *SALL3*, located in 18q, is responsible for this impairment
• Downregulation of *SALL3* deregulates pluripotency and differentiation pathways

## Teaser

hPSC cultures commonly acquire recurrent chromosomal abnormalities, among which is loss of chromosome 18q. Lei and colleagues show that this aneuploidy impedes neuroectoderm commitment due to decreased expression of SALL3, a gene located in the common region of deletion. SALL3 deregulation alters pluripotency and differentiation-associated gene expression. Its knockdown mimics 18q loss, and its overexpression rescues neuroectoderm differentiation.

## Introduction

Human pluripotent stem cells (hPSCs), including human embryonic stem cells (hESCs) and induced pluripotent stem cells (hiPSCs), are self-renewing cells that can give rise to any cell type originating from any of the three embryonic germ layers. This makes hPSCs an attractive resource for *in vitro* disease modeling, developmental biology research, drug discovery, and cell transplantation therapy. A substantial number of clinical trials are under way using hPSC-derived cell products, including for the treatment of age-related macular degeneration, spinal cord injury, and type 1 diabetes (Kobold et al., 2020; Yamanaka, 2020). An important hurdle for both safe clinical translation and the reliable use of hPSCs as *in vitro* research models is the occurrence of cell culture drift due to the acquisition of genetic abnormalities (Andrews et al., 2022). A subset of these genetic aberrations is highly recurrent, and these aberrations are found in hPSC lines worldwide. These recurrent changes vary in size from single-nucleotide point mutations to large chromosome structural variants, the most common being gains of chromosomes 1q, 12p, 17, 20, and X and losses of 10p, 18q and 22p, as well as mutations in *TP53* (Amps et al., 2011; Merkle et al., 2017, 2022). Other genetic changes include epigenetic variations, including erosion of X chromosome inactivation (Bar and Benvenisty, 2019; Geens et al., 2016), mutations in the mitochondrial genome (Van Haute et al., 2013; Zambelli et al., 2018), and an array of other point mutations and structural variants spread throughout the genome (Avior et al., 2019; Merkle et al., 2022).

These recurrent genetic changes arise from the pool of common variations in hPSC cultures via different cell competition mechanisms. hPSCs are prone to replication stress, leading to DNA damage, which in turn is a source of *de novo* genetic variation (Halliwell et al., 2020; Jacobs et al., 2014). For instance, up to 20% of cells in an hESC culture carry *de novo* structural variants, but only a minority of them have the potential to confer a selective advantage to the cells (Jacobs et al., 2014, 2016; Keller et al., 2019), leading to the mutant cells rapidly outcompeting their genetically balanced counterparts (Avery et al., 2013; Nguyen et al., 2014; Olariu et al., 2010; Price et al., 2021). The exact traits that the different chromosomal abnormalities confer on undifferentiated cells, as well as the specific driver genes of these traits, are only well established for the gain of 20q11.21 (Spits et al., 2008). This abnormality confers a decreased sensitivity to apoptosis-inducing events due to increased expression of the gene *BCL2L1*, located in the minimal gained region (Amps et al., 2011; Avery et al., 2013; Nguyen et al., 2014). For gains of 12p, it is thought that *NANOG* drives at least part of the growth advantage of the cells (Ben-David et al., 2014), and cells with a complex karyotype carrying all of the most common abnormalities (gains of 1, 12, 17 and 20q) can outcompete the other cells by corralling and mechanical compression (Price et al., 2021).

An important concern about these genetic variants is whether and how they alter the differentiation capacity of hPSCs and potentially prime differentiated cells for malignant transformation (Andrews et al., 2022; Keller et al., 2018). The gain of 1q is common in cancers, particularly lung adenocarcinoma, breast invasive carcinoma, and liver hepatocellular carcinoma (Taylor et al., 2018), and appears to confer a growth advantage to cells during differentiation from hESCs to neural precursors (Varela et al., 2012). Moreover, variants in 1q21.1 can alter neurodevelopmental trajectories upon hiPSC differentiation, with the deletion of 1q21.1 accelerating neuronal production and its duplication delaying the transition from neural progenitor cell to neuron (Chapman et al., 2022). Gains in chromosome 12 are frequently found in testicular germ cell tumors (Baker et al., 2007). hPSCs with trisomy 12 display a reduced tendency toward spontaneous differentiation (Ben-David et al., 2014). The highly recurrent gain of 20q11.21 impairs the neuroectodermal lineage commitment of hPSCs (Jo et al., 2020; Markouli et al., 2019), and hESCs with 20q11.1q11.2 amplification have a reduced propensity to differentiate down the hematopoietic lineage, maintain more immature phenotypes along the neural differentiation trajectory, and generate teratomas with foci of undifferentiated cells (Werbowetski-Ogilvie et al., 2009). The gain of chromosome 17 is common in neuroblastomas, testicular germ cell tumors, and breast cancers (Baker et al., 2007), and hPSC lines with a gain of chromosome 17 show altered differentiation patterns in embryoid bodies (Fazeli et al., 2011).

Deletions of chromosome 18q are one of the rarer recurrent structural chromosomal abnormalities in hPSCs. This deletion was first reported as a single event by Maitra et al. in 2005 (Maitra et al., 2005), and our group later found 18q deletions in three different hESC lines at relatively early passages, always as part of a derivative chromosome 18 (Spits et al., 2008). A large study by Amps et al. in 2011 revealed 5 instances of this deletion in hESC (Amps et al., 2011), and WiCell reported that 4% of the 7,300 hPSC cultures evaluated over nearly 8 years carried an 18q deletion (WiCell Cytogenetics Lab, https://www.wicell.org/media.acux/29102c0e-e88e-426b-ab7d-bac4c2a9ec6a). Chromosome 18q loss is common in cancers, especially gastrointestinal tract cancers (Taylor et al., 2018), and is linked to several disorders, including congenital malformations, developmental delays, and intellectual disability (Hogendorf et al., 2021). However, the impact of 18q loss on the functional properties of hPSCs is unknown. Therefore, the aim of this work was to examine the functional effects of 18q deletions during hPSC differentiation into the three embryonic germ layers and to determine the molecular mechanisms involved.

## Results

### The minimal common region of 18q loss spans 14 genes expressed in undifferentiated hESCs and includes *SALL3*

We initially identified 18q losses in the hESC lines Vrije Universiteit Brussel (VUB)04 and VUB26, in the form of a derivative chromosome 18. VUB04 presented a deletion at 18q21.2qter and a duplication at 5q14.2qter, and VUB26 showed the minimal 18q loss region (18q23qter) and a duplication at 7q33qter (Figure S1; Table S1) (Spits et al., 2008). These two hESC lines were not used in the present work because they further genetically drifted and acquired gain of 1q and 20q11.21, but their analysis helped to narrow down the common 18q deletion region. In the present study, we used two other hESC lines bearing derivative chromosomes 18 involving a loss of 18q losses (hESC^del18q^), VUB14^del18q^, and VUB13^del18q^, as well as three chromosomally balanced lines (hESC^WT^ [wild type]), VUB14^WT^, and VUB04^WT^ and VUB03^WT^, which served as controls for VUB13^del18q^ since VUB13^WT^ was lost (details on the karyotypes of the lines and their characterization are shown in Figure S2; Table S1). We used shallow genome sequencing to confirm the karyotypes before starting the experiments, and all of the lines were routinely inspected, with qPCR assays targeting recurrent chromosomal abnormalities (1q, 12p, 20q11.21, and 17q) to confirm their genomic stability for the duration of the different experiments.

The 18q losses exhibited a common loss region from base pairs 75,773,285 to 80,373,285, spanning 37 loci. Bulk RNA sequencing (RNA-seq) of undifferentiated hESCs indicated that 14 genes within this region are expressed in undifferentiated hESCs, with counts per million greater than one in at least two samples (Figure S1A). Of these coding genes, *ADNP2*, *SALL3*, and *TXNL4A* had the highest expression and showed decreased transcript levels in mutant cells. *ADNP2* and *TXNL4A* have no known function in hPSC. *ADNP2* is predicted to be a transcription factor, and its silencing increases oxidative stress-mediated cell death (Kushnir et al., 2008). *TXNL4A* is a component of the U5 small ribonucleoprotein particle, which is involved in pre-mRNA splicing and is associated with Burn-McKeown syndrome (Wood et al., 2022). *SALL3* was more promising as a candidate driver gene because it has previously been reported to regulate the differentiation propensity of hiPSC lines (Kuroda et al., 2019). Kuroda et al. showed that hiPSC lines expressing high levels of *SALL3* differentiated preferentially into ectoderm, whereas hiPSC lines expressing lower levels of *SALL3* tended to differentiate into mesoderm and endoderm (Kuroda et al., 2019). It has also been shown that *SALL3* interacts with the Mediator complex in neural stem cells (Quevedo et al., 2019) and is related to the development of the nervous system (Ott et al., 1996). Considering these previous findings, we hypothesized that the decreased expression of *SALL3* because of a loss of one copy of the gene could alter the differentiation capacity of hESCs^del18q^.

### hESCs with 18q loss show impaired neuroectoderm differentiation

**Figure 1:**
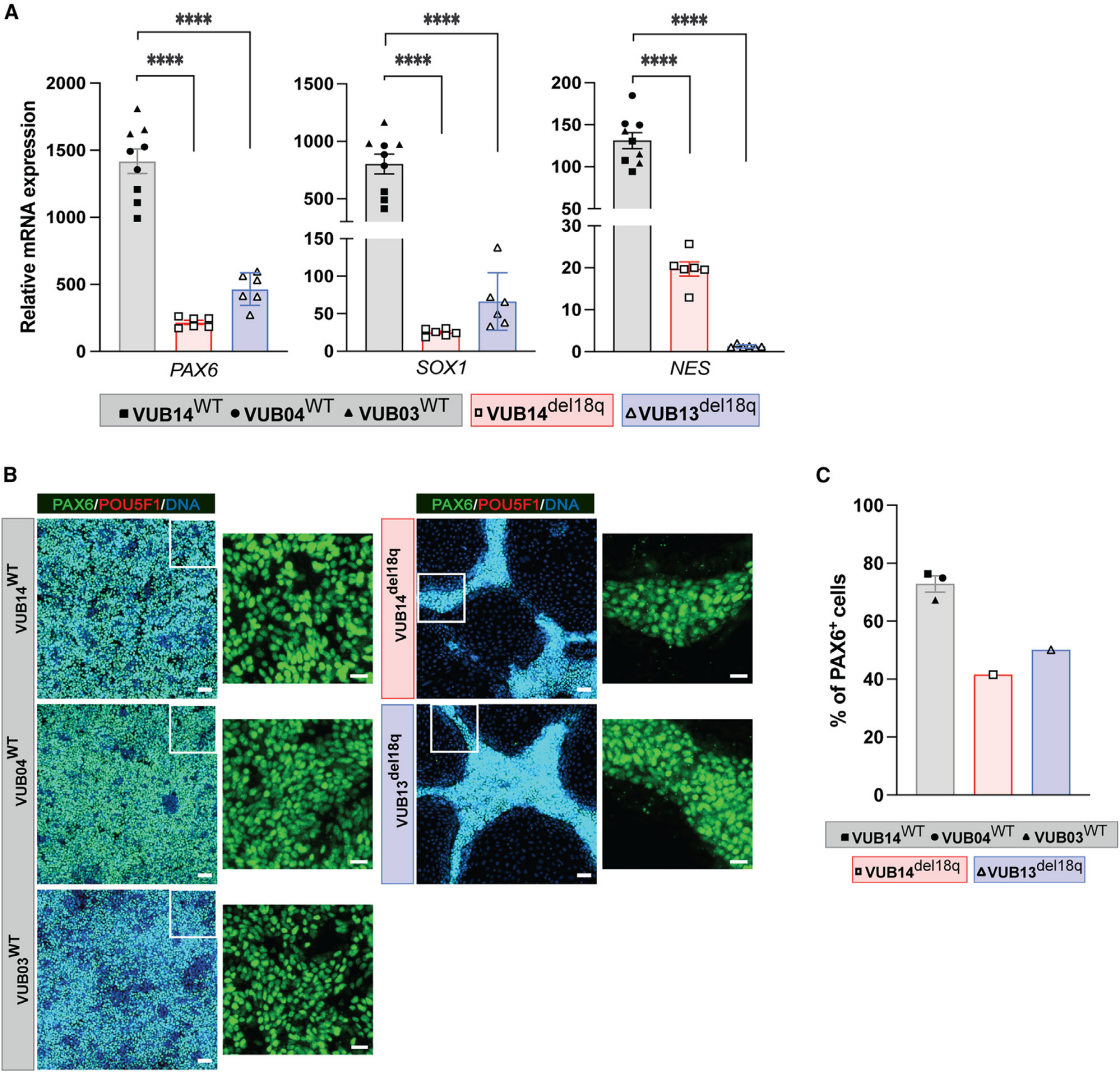
hESCs with 18q loss show impaired neuroectoderm differentiation (A) Relative mRNA expression for neuroectoderm markers. Data are shown as means ± SEMs. Each data point refers to an independent differentiation experiment, and ^∗^, ^∗∗^, ^∗∗∗^, and ^∗∗∗∗^ represent statistical significance between samples at 5%, 1%, 0.1%, and 0.01%, respectively (unpaired t test). (B) Immunostaining in mutant and control lines. Scale bars (original images), 50 μm, scale bars (magnification images), 20 μm. (C) Percentages of PAX6^+^ cells in the immunostainings shown in (B).

As a first step, we investigated the effect of 18q deletion on hESC ectoderm lineage commitment. hESC^WT^ and hESC^del18q^ were subjected to neuroectoderm differentiation for 8 days using LDN193189 (LDN), SB431542 (SB) and retinoic acid (RA) (Douvaras and Fossati, 2015) (Figure S1B). We measured the mRNA levels of different neuroectoderm markers to evaluate neuroectoderm differentiation efficiency and the expression of the undifferentiated state markers *NANOG* and *POUF51* (Figures 1A and S3A). VUB13^del18q^ and VUB14^del18q^ had significantly lower mRNA levels of *PAX6*, *NES*, and *SOX1*, compared to the levels in hESC^WT^ (for all genes and both lines, p ≤ 0.0001, unpaired t test), indicating a decreased neuroectodermal differentiation efficiency in hESC^del18q^. *POU5F1* and *NANOG* mRNA expression levels were almost undetectable for all differentiated cells (Figure S3A). We also evaluated the differentiation of hESC^WT^ and hESC^del18q^ cells by immunostaining (Figures 1B and 1C). We observed a lower percentage of PAX6^+^ cells in differentiated hESC^del18q^ than in differentiated hESC^WT^ cells (70% in hESC^WT^ vs. 50% in VUB13^del18q^ and 41% in VUB14^del18q^; Figure 1C), which was consistent with the decrease in the levels of *PAX6* mRNA. Taken together, these results show that hESC^del18q^ differentiation into neuroectoderm is impaired, and rather than to remain undifferentiated state, they misspecify.

### hESC^del18q^ readily differentiates into mesendoderm derivatives but shows abnormal cardiomyocyte progenitor differentiation

**Figure 2:**
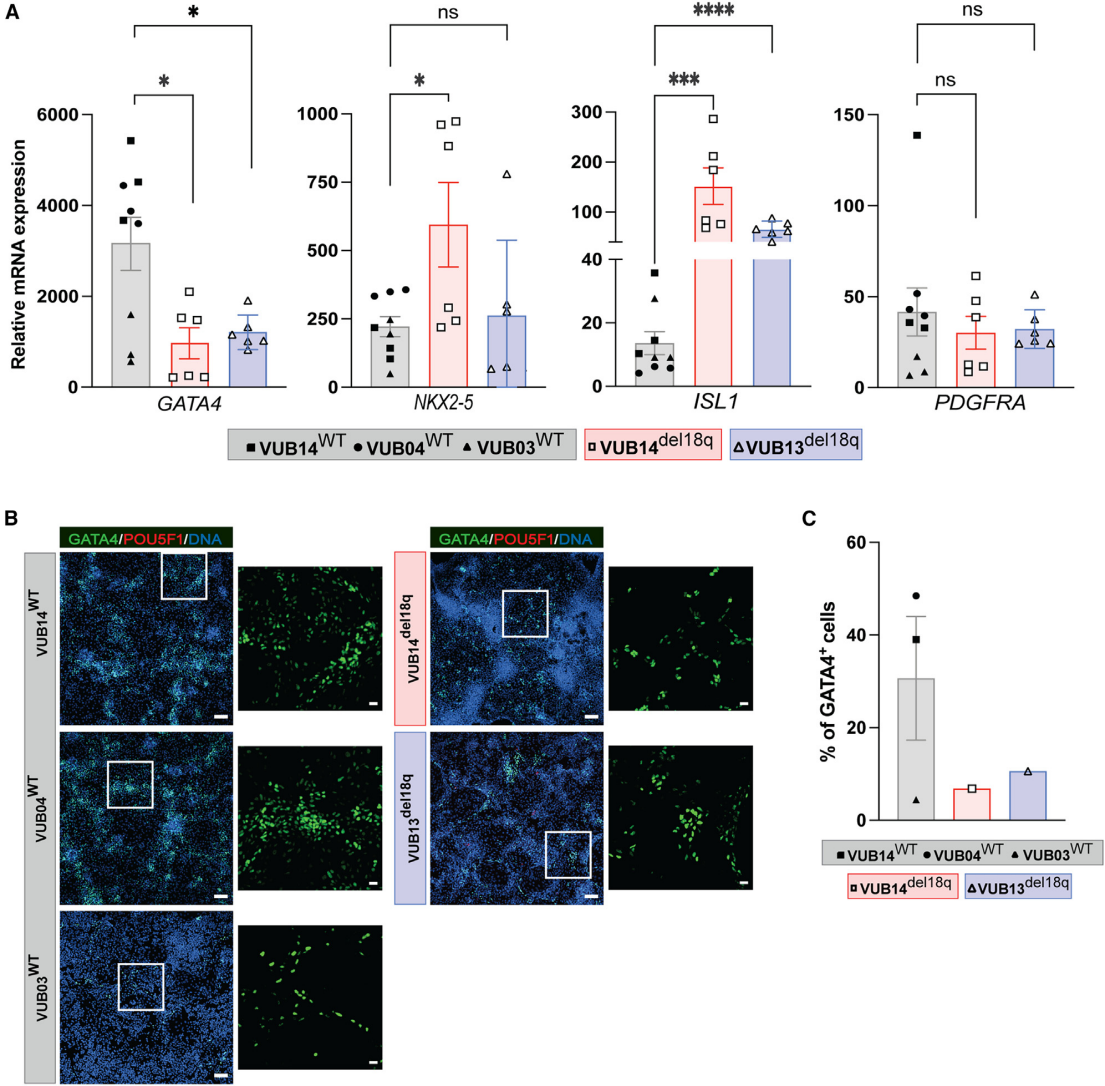
hESC^del18q^ cells show abnormal cardiac progenitor differentiation (A) Relative mRNA expression for cardiac progenitor markers. Data are shown as means ± SEMs. Each data point refers to an independent differentiation experiment, and ^∗^, ^∗∗^, ^∗∗∗^, and ^∗∗∗∗^ represent statistical significance between samples at 5%, 1%, 0.1%, and 0.01% respectively (unpaired t test). ns, not significant. (B) Immunostaining for GATA4 and POU5F1 in mutant and control lines. Scale bars (original images), 100 μm, and scale bars (magnification images), 20 μm. (C) Percentage of GATA4^+^ cells in the immunostainings shown in (B).

We further investigated the impact of 18q deletion on the mesendoderm differentiation capacity of hESCs by differentiating hESC^WT^ and hESC^del18q^ into, in this case, cardiac progenitors and hepatoblasts. Thus, we induced the differentiation of hESC^WT^ and hESC^del18q^ into mesoderm using a 5-day cardiac progenitor induction protocol described previously (Lin and Zou, 2020) (Figure S1B). We evaluated the mRNA levels of the cardiac progenitor markers *GATA4*, *ISL1*, *NKX2-5*, and *PDGFRA* (Figure 2A). hESC^del18q^ showed lower levels of *GATA4* mRNA (p = 0.02 for VUB13^del18q^ and p = 0.01 VUB14^del18q^, unpaired t test; Figure 2A). hESC^del18q^ lines expressed *ISL1* at 15-fold higher levels, on average, than hESC^WT^ lines (p < 0.0001 for VUB13^del18q^ and p = 0.0005 VUB14^del18q^, unpaired t test; Figure 2A). We found no significant or consistent difference in the expression levels of *NKX2-5* or *PDGFRA* between the control and mutant cardiac progenitor groups (p = 0.01 for *NKX2-5* VUB14^del18q^, others not significant, unpaired t test; Figure 2A). We also evaluated the proportion of differentiated cardiac progenitor and undifferentiated cells by immunostaining for GATA4 and POU5F1. The percentage of GATA4^+^ cells was overall lower in hESC^WT^ than in hESC^del18q^, whereas the cells were POU5F1^−^ (Figures 2B and 2C). Taken together, and bearing in mind the temporal expression of these markers during cardiac differentiation (Doyle et al., 2015; Zhang et al., 2019), the results suggest that hESC^del18q^ may experience differentiation delays or arrest, reaching an ISL1^high^ GATA4^low^ stage at day 5 but remaining less mature than their hESC^WT^ counterparts.

**Figure 3:**
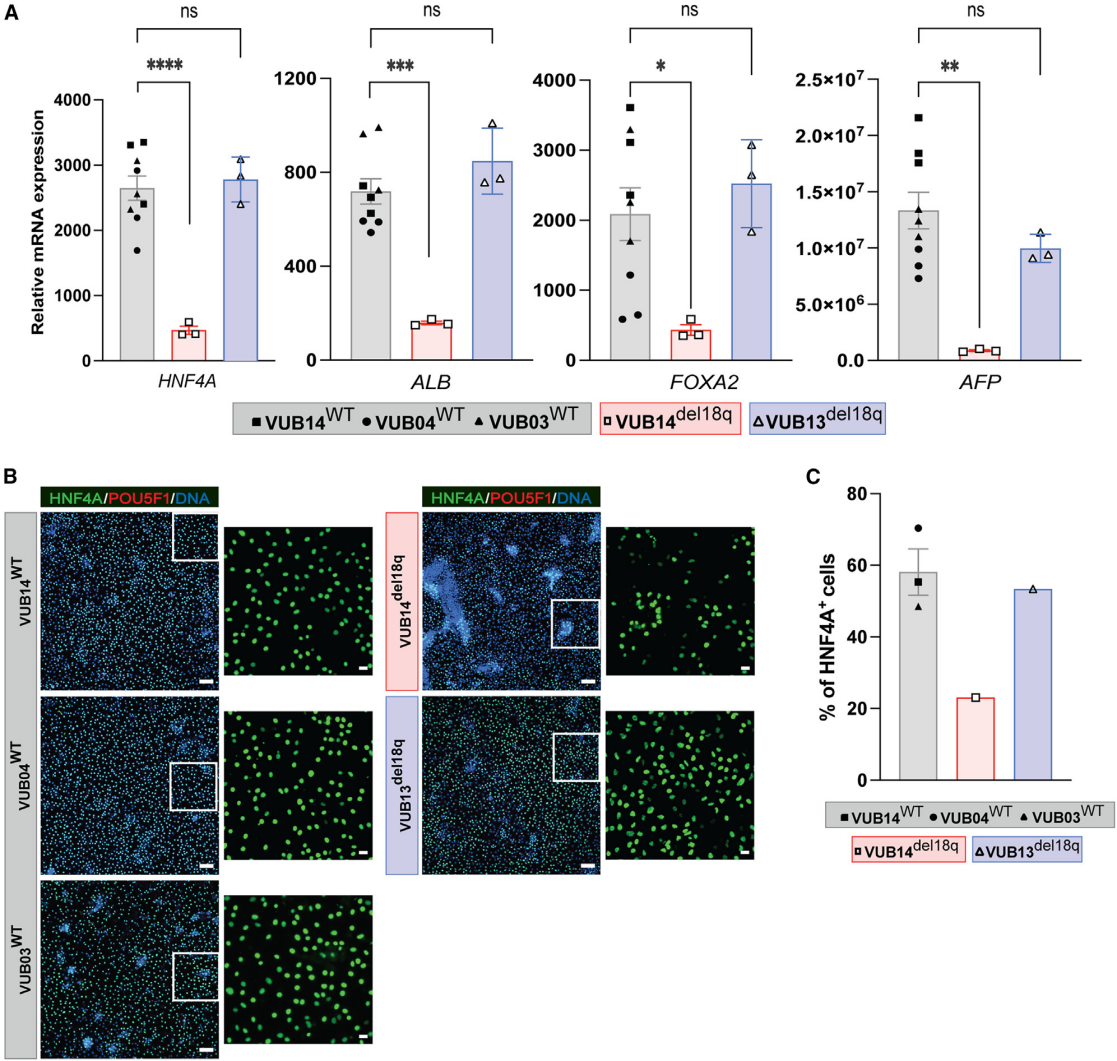
hESC^del18q^ and their genetically balanced counterparts differentiate equally well into hepatoblasts (A) Relative mRNA expression of hepatoblast markers. Data are shown as means ± SEMs. Each data point refers to an independent differentiation experiment, and ^∗^, ^∗∗^, ^∗∗∗^, and ^∗∗∗∗^ represent statistical significance between samples at 5%, 1%, 0.1%, and 0.01%, respectively (unpaired t test). (B) Immunostaining for HNF4A and POU5F1 in mutant and control lines. Scale bars (original images), 100 μm, and scale bars (magnification images), 20 μm. (C) Percentage of HNF4A^+^ cells in the immunostainings shown in (B).

Next, we differentiated hESC^WT^ and hESC^del18q^ into hepatoblasts by applying the modified differentiation protocol for 8 days, as described previously (Boon et al., 2020) (Figure S1B). We measured the expression levels of the hepatoblast markers *HNF4A*, *AFP*, *ALB*, and *FOXA2* (Figure 3A). We found no consistent difference in the mRNA expression levels of *HNF4A*, *ALB*, *FOXA2*, and *AFP* between hESC^WT^ and hESC^del18q^ (not significant for VUB13^del18q^, p < 0.0001, p = 0.0002, p = 0.03, and p = 0.0016, respectively, for VUB14^del18q^, unpaired t test; Figure 3A). We further evaluated hepatoblast differentiation by immunostaining for HNF4A and found that the percentages of HNF4A^+^ cells in hESC^del18q^ cells were in line with the mRNA expression. VUB13^del18q^ showed results similar to those in WT cells (58% in hESC^WT^ vs. 53% in VUB13^del18q^), whereas VUB14^del18q^ had 23% positive cells (Figures 3B and 3C).

For both mesodermal and endodermal lineage commitment, all hESC^WT^ and hESC^del18q^ lines displayed low mRNA and protein levels of undifferentiated state markers (Figures S3B and S3C), indicating a loss of pluripotency for all cell lines during differentiation. Overall, our results indicate that hESC^WT^ and hESC^del18q^ differentiate into definitive mesoderm and endoderm and that there may be a delay or impairment in the progression of hESC^del18q^ toward the cardiac progenitor stage. The differences in gene expression observed during hepatoblast differentiation are likely due to between-line variation in differentiation propensity rather than to the 18q deletion itself.

### Downregulation of *SALL3* impairs neuroectoderm differentiation but does not affect differentiation into mesoderm and endoderm

We next examined *SALL3* mRNA expression levels in undifferentiated cells and found that *SALL3* expression was significantly lower in hESC^del18q^ than in hESC^WT^ (Figures S1A and S3D), supporting the notion that *SALL3* could be a key gene in the altered differentiation capacity of hESCs^del18q^. We first generated *SALL3* knockdown (KD) lines from three hESC^WT^ lines (VUB02^WT^, VUB03^WT^, and VUB04^WT^) by transducing a lentiviral vector containing short hairpin RNA (shRNA) targeting the *SALL3* transcript (hESC^WT_*SALL3*KD^) or a nontargeting shRNA as a control (hESC^WT−NT^) (Figure S3E). Next, we generated hESC^del18q^ with stable overexpression (OE) of *SALL3* (hESC^del18q_*SALL3*OE^) by transducing VUB13^del18q^ and VUB14^del18q^ with the *SALL3* lentiviral vector, and we verified the OE by measuring *SALL3* mRNA levels, which were significantly increased in VUB13^del18q_*SALL3*OE^ (3-fold) and VUB14^del18q_*SA LL3*OE^ (5-fold) compared to controls (Figure S3F).

**Figure 4:**
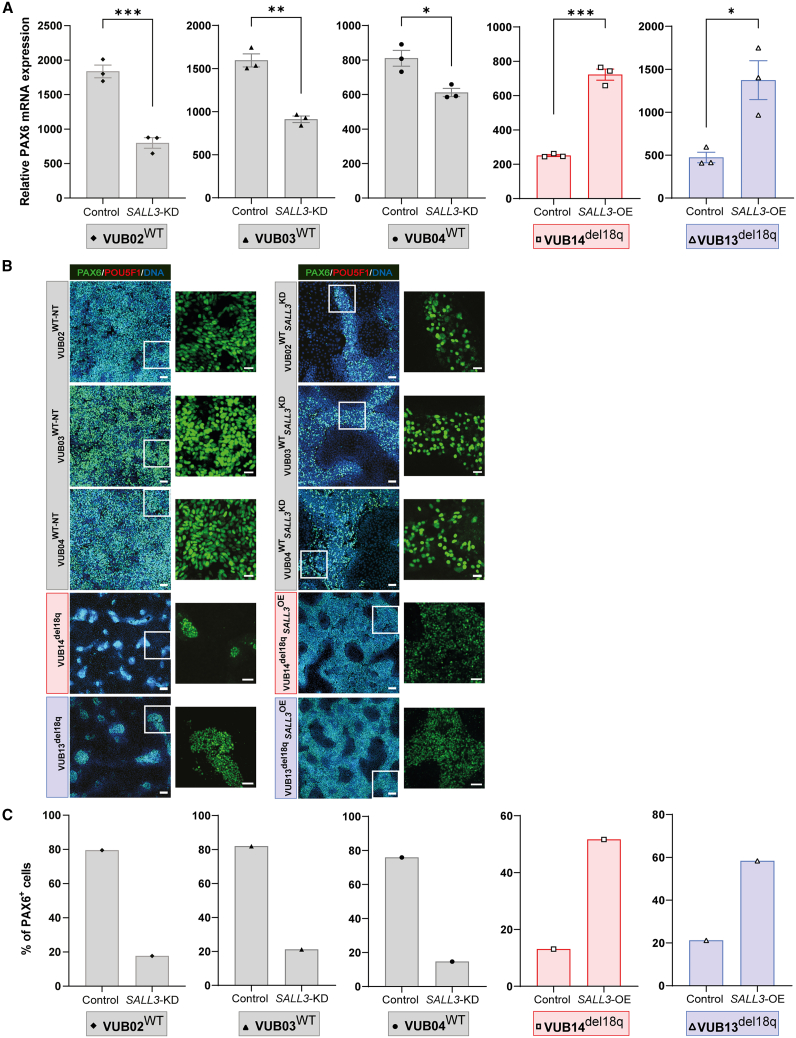
Downregulation of *SALL3* drives the impaired neuroectoderm differentiation of hESCs with 18q loss (A) Relative mRNA expression for *PAX6*. Data are shown as means ± SEMs. Each data point refers to an independent differentiation experiment, and ^∗^, ^∗∗^, ^∗∗∗^, and ^∗∗∗∗^ represent statistical significance between samples at 5%, 1%, 0.1%, and 0.01%, respectively (unpaired t test). (B) Immunostaining for PAX6 and POU5F1 in mutant and control lines. Scale bars (KD groups), 50 μm, and magnification 20 μm; scale bars (OE groups), 100 μm, and magnification, 50 μm. (C) Percentage of PAX6^+^ cells in the immunostainings shown in (B).

To investigate the role of *SALL3* in regulating hESC differentiation propensity, we first induced neuroectodermal differentiation in hESC^WT_*SALL3*KD^, hESC^del18q_*SALL3*OE^, and the corresponding control cells. All three hESC^WT_*SALL3*KD^ lines had lower levels of all neuroectodermal (NE) markers than nontarget controls (Figures 4A, S4A, and S4B). PAX6 protein levels were also reduced in hESC^WT_*SALL3*KD^ (Figures 4B and 4C), with only 20% of the cells expressing PAX6, compared to 80% of PAX6^+^ cells in the control group (Figures 4B and 4C). Our results show that *SALL3* suppression in hESC^WT^ recapitulates the impaired NE differentiation seen in hESC^del18q^ lines. In contrast, hESC^del18q_*SALL3*OE^ cells efficiently differentiated into neuroectoderm cells, accompanied by a significant increase in the mRNA levels of *PAX6*, *SOX1*, and *NES* (Figures 4A, S4A, and S4B); moreover, hESC^del18q_*SALL3*OE^ cultures included more PAX6^+^ cells than hESC^del18q^ cultures (60% vs. 20%, respectively) (Figures 4B and 4C). These results indicate that exongenous *SALL3* expression can rescue the impairment of ectoderm differentiation caused by 18q loss.

**Figure 5:**
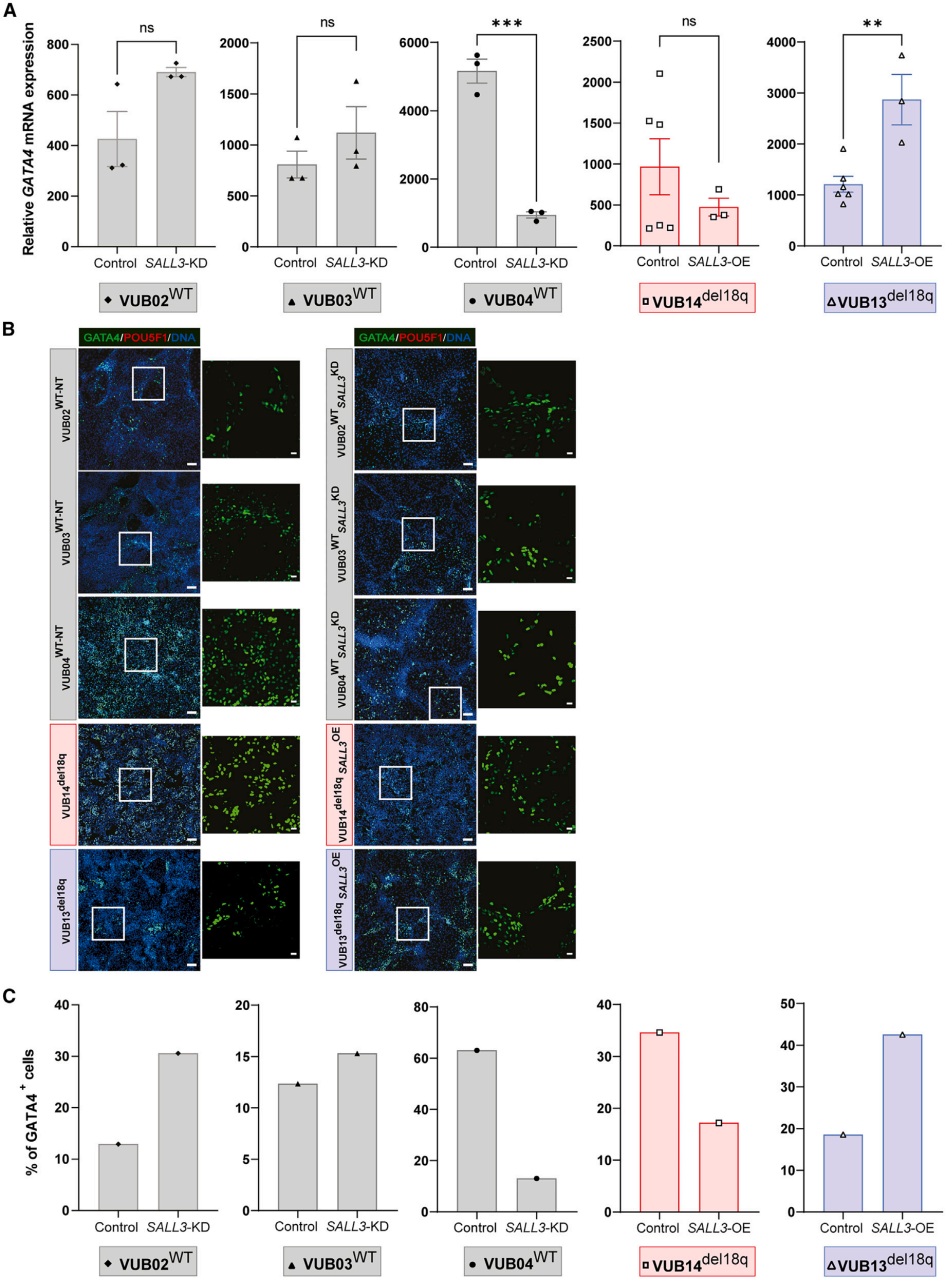
Changes in *SALL3* expression do not regulate cardiac progenitor differentiation (A) Relative mRNA expression for *GATA4*. Data are shown as means ± SEMs. Each data point refers to an independent differentiation experiment, and ^∗^, ^∗∗^, ^∗∗∗^, and ^∗∗∗∗^ represent statistical significance between samples at 5%, 1%, 0.1%, and 0.01%, respectively (unpaired t test). (B) Immunostaining for GATA4 and POU5F1 in mutant and control lines. Scale bars (original images), 100 μm, and scale bars (magnification images), 20 μm. (C) Percentage of GATA4^+^ cells in the immunostainings shown in (B).

Next, we induced the differentiation of the different hESC lines into cardiac progenitors. The three hESC^WT_*SALL3*KD^ lines differentiated inconsistently toward mesoderm fates. VUB04^WT_*SALL3*KD^ showed lower levels of *GATA4* (Figure 5A; p = 0.0003, unpaired t test), whereas compared to the controls, VUB03^WT_*SALL3*KD^ and VUB02^WT_*SALL3*KD^ showed no differences in *GATA4* expression (Figure 5A). In addition, the percentage of GATA4^+^ cells detected by immunostaining in hESC^WT_*SALL3*KD^ followed the same pattern, consistent with the mRNA levels of each line (Figures 5B and 5C). The hESC^del18q_*SALL3*OE^ cells exhibited variable marker profiles, with increases in GATA4 expression at both the mRNA (Figure 5A; p = 0.004, unpaired t test) and protein levels for VUB13^del18q_*SALL3*OE^, but no difference in *GATA4* mRNA levels (Figure 5A; p = 0.3622, unpaired t test) and a slight decrease in GATA4 protein levels in VUB14^del18q_*SALL3*OE^ (Figures 5B and 5C). Similarly, the mRNA expression levels of other markers, *NKX2-5*, *ISL1*, and *PDGFRA*, showed no consistent trend in the hESC^WT_*SALL3*KD^ groups and exhibited no consistent differences in hESCs^del18q^ and hESC^del18q*SALL3*OE^ (Figures S5A–S5C).

**Figure 6:**
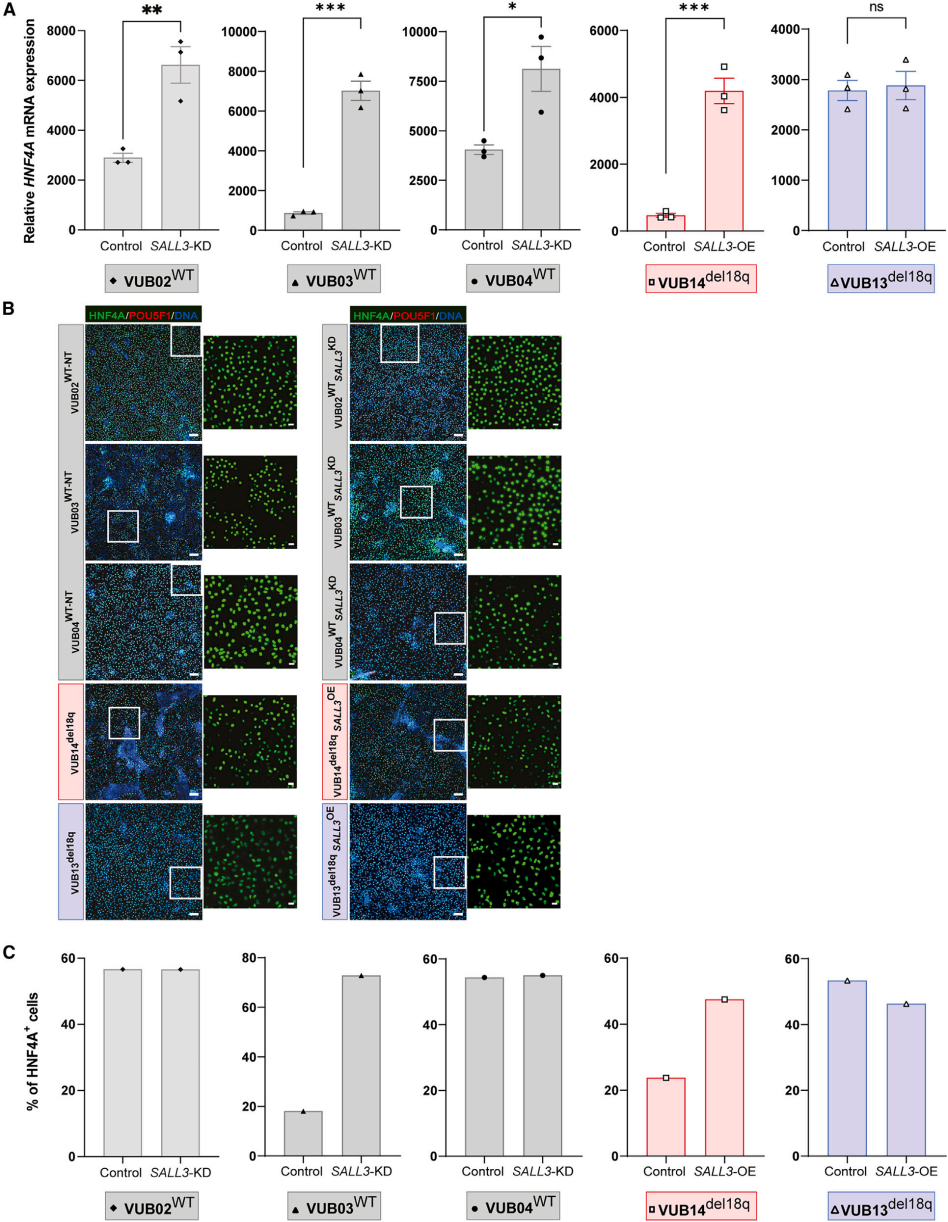
Changes in *SALL3* expression do not consistently affect hepatoblast differentiation (A) Relative mRNA expression of *HNF4A*. Data are shown as means ± SEMs. Each data point refers to an independent differentiation experiment, and ^∗^, ^∗∗^, ^∗∗∗^, and ^∗∗∗∗^ represent statistical significance between samples at 5%, 1%, 0.1%, and 0.01%, respectively (unpaired t test). (B) Immunostaining for HNF4A and POU5F1 in mutant and control lines. Scale bars (original images), 100 μm, and scale bars (magnification images), 20 μm. (C) Percentage of HNF4A^+^ cells.

When hESCs were differentiated into hepatoblast, *SALL3* downregulation in hESC^WT^ resulted in increased *HNF4A*, *ALB*, and *FOXA2* mRNA expression (Figures 6A, S6B, and S6C). The percentage of HNF4A^+^ cells was higher in VUB03^WT_*SALL3*KD^ cells, but not in VUB04^WT_*SALL3*KD^ and VUB02^WT_S*ALL3*KD^ cells compared to controls (Figures 6B and 6C). The mRNA expression of another marker, *AFP*, also did not show consistent changes (Figure S6A). Upon the OE of *SALL3*, the differentiation profiles into hepatoblast did not show the expected mirroring effect. The mRNA expression of all of the hepatoblast markers *HNF4A*, *ALB*, *AFP*, and *FOXA2* increased in VUB14^del18q_*SALL3*OE^ cells, consistent with the changes observed in the HNF4A protein level, but this same effect was not observed in VUB13^del18q_*SALL3*OE^ cells (Figures 6 and S6).

Overall, these results suggest that the effect of *SALL3* on mesoderm and endoderm differentiation may be line specific and is not the reason for the delayed progression seen during cardiac differentiation in hESCs^18q^.

### Downregulation of *SALL3* and loss of 18q result in the deregulation of genes in pathways associated with pluripotency and differentiation

**Figure 7:**
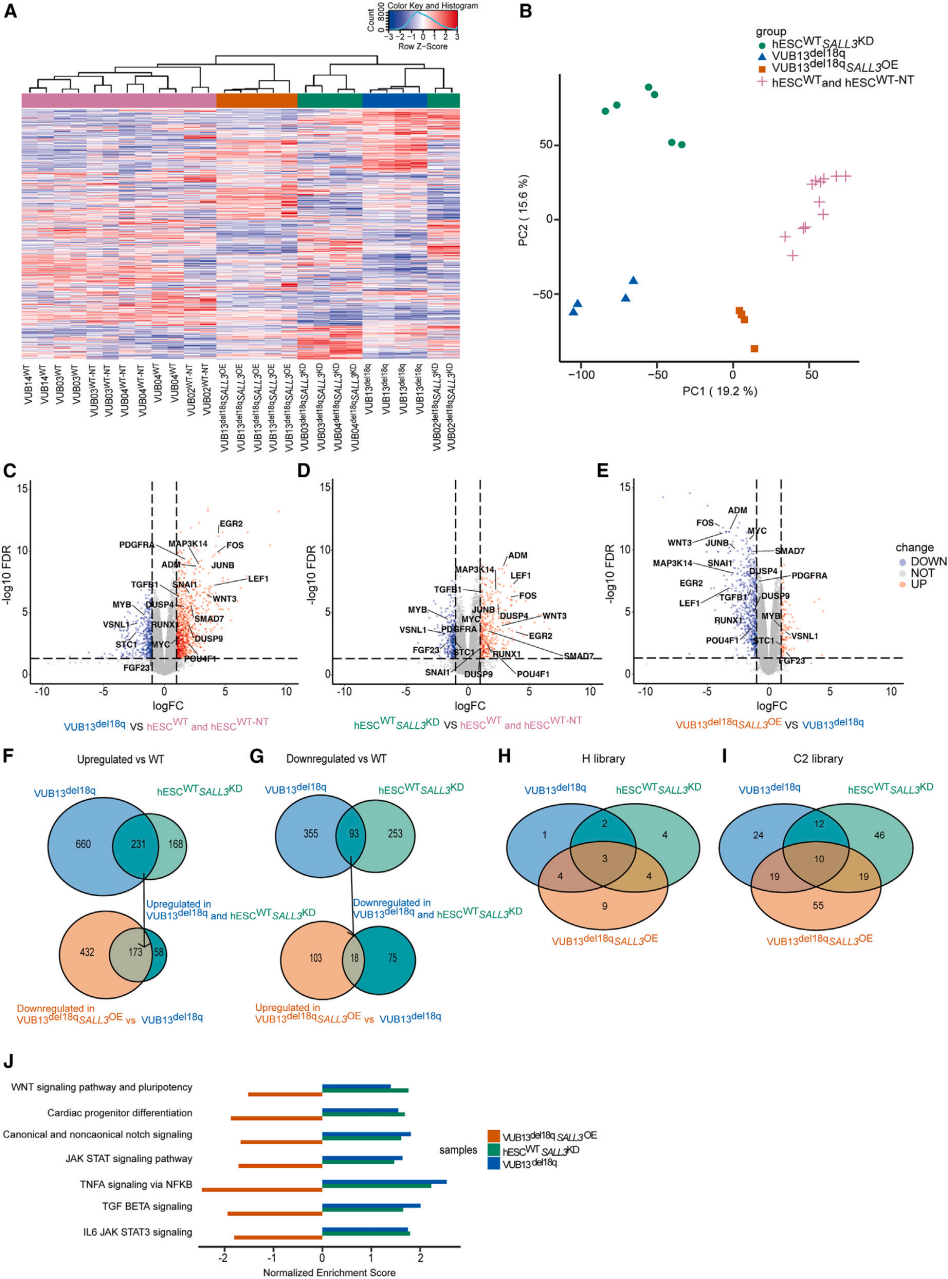
Downregulation of *SALL3* and loss of 18q result in the deregulation of genes in pathways associated with pluripotency and differentiation (A and B) Unsupervised clustering heatmap (A) and PCA (B) of the coding genes with a count per million >1 in at least 2 samples. (C–E) Volcano plots of differential gene expression analysis for VUB13^del18q^ vs. hESC^WT^ and hESC^WT−NT^. The labeled genes are the top 20 commonly deregulated genes across the 3 comparisons (C), hESC^WT_*SALL3*KD^ vs. hESC^WT^ and hESC^WT−NT^ (D), and VUB13^del18q_*SALL3*OE^ vs. VUB13^del18q^ (E), with a cutoff value of |log_2_fold change|> 1 and FDR < 0.05. (F) Venn diagrams show the genes downregulated in VUB13^del18q_*SALL3*OE^ and upregulated in VUB13^del18q^ and hESC^WT_*SALL3*KD^. (G) Venn diagrams showing the genes upregulated in VUB13^del18q_*SALL3*OE^ and downregulated in VUB13^del18q^ and hESC^WT_*SALL3*KD^. (H) Venn diagrams of the pathways in the H library common among VUB13^del18q^, hESC^WT_*SALL3*KD^, VUB13^del18q_*SALL3*OE^, and hESC^WT^. (I) Venn diagrams of the pathways in the C2 library common among VUB13^del18q^, hESC^WT_*SALL3*KD^, VUB13^del18q_*SALL3*OE^, and hESC^WT^. (J) Pathways commonly deregulated among the different samples are associated with pluripotency and differentiation.

To gain deeper insight into the effect of *SALL3* downregulation on the global transcriptomic profile of cells with 18q loss, we carried out bulk RNA-seq of hESC^WT−NT^ (N = 6), hESC^WT_*SALL3*KD^ (N = 6), hESC^WT^ (N = 6), VUB13^del18q^ (N = 4), and VUB13^del18q_*SALL3*OE^ (N = 5) cells. To estimate the similarity among the samples, we generated a distance clustering heatmap with global row scaling (Figure 7A) and performed principal-component analysis (PCA) (Figure 7B). The heatmap shows that although VUB13^del18q^ and hESC^WT_*SALL3*KD^ cluster together, they cluster apart from the WT cell lines and from VUB13^del18q_*SALL3*OE^. The samples in this last group clustered more closely to the WT cell lines than to their unmodified VUB13^del18q^ (Figure 7A). This pattern was also reflected in the first dimension of the PCA (Figure 7B), in which hESC^WT^, hESC^WT−NT^, and VUB13^del18q_*SALL3*OE^ clustered more closely with one another than with VUB13^del18q^ and hESC^WT_*SALL3*KD^. These results suggest that the downregulation of *SALL3* in WT cells is sufficient to alter the transcriptome such that its profile is closer to that of an hESC line with a loss of 18q, and *SALL3* OE in hESC^del18q^ can restore the transcriptome to a near-WT state. Taken together, these findings support our hypothesis that the differences between hESC^WT^ and hESC^del18q^ are driven mostly by the downregulation of *SALL3* due to the loss of one copy of this gene. For this reason, we pooled the hESC^WT−NT^ with the untreated hESC^WT^ for further analysis.

Next, we carried out differential gene expression analysis to gain further insight into which genes and pathways form the basis of the differences between hESC^WT^ and hESC^del18q^ and which of these are driven by *SALL3*. Figures 7C–7E show volcano plots of the differentially expressed genes in the different groups. We considered this differential expression to be significant at a |log_2_fold change| > 1.0 and false discovery rate (FDR) < 0.05. VUB13^del18q^ shows 891 and 448 genes that are significantly upregulated and downregulated in hESC^del18q^, respectively, compared to the WT cells (Figure 7C). Downregulation of *SALL3* in WT cells led to the differential expression of 745 genes, 399 of which were upregulated and 346 of which were downregulated (Figure 7D). OE of *SALL3* in VUB13^del18q^ resulted in the upregulation of 121 genes and the downregulation of 605 genes (Figure 7E).

To elucidate which of the transcriptional differences between hESC^WT^ and hESC^del18q^ are mediated by *SALL3*, we investigated the overlaps in up- and downregulated genes across conditions. First, we found that 231 and 93 genes were commonly up- and downregulated, respectively, between VUB13^del18q^ and hESC^WT_*SALL3*KD^, representing 26% of the differentially expressed genes in hESCs with an 18q deletion (Figures 7F and 7G). Second, we compared these subsets of genes to the genes with altered gene expression in hESC^del18q^ upon OE of *SALL3*. We compared the genes that were upregulated by *SALL3* KD and by loss of 18q to those downregulated by OE of *SALL3* in hESC^18q^, and vice versa (Figures 7F and 7G). Of the 231 upregulated genes shared by the VUB13^del18q^ and hESC^WT_*SALL3*KD^ groups, 173 genes showed increased expression in VUB13^del18q_*SALL3*OE^ (Figure 7F). In addition, 18 of the 93 downregulated genes shared by VUB13^del18q^ and hESC^WT_*SALL3*KD^ displayed increased expression in VUB13^del18q^*SALL3*^OE^ (Figure 7G). This further refined the gene set to a core of 191 genes that are the most strongly regulated by *SALL3* in hESCs, both by the loss of a copy of the gene itself and by the modulation of its expression (Table S2). The other differences in gene expression are likely associated with the loss of other genes in 18q or with the OE of genes in the duplicated regions of chromosomes 5 and 7, which are part of the derivative chromosome 18 in hESC^del18q^.

Finally, to identify potential molecular targets and elucidate the underlying functional mechanisms that contribute to the observed impairment of differentiation capacity in hESC^del18q^, we analyzed the differential gene expression of the three groups using gene set enrichment analysis and the MSigDB database. Specifically, we focused on the Kyoto Encyclopedia of Genes and Genomes and WikiPathways databases within the C2 library and the pathways of the H library. We filtered the significant pathways based on a normalized enrichment score |NES| > 1, a p < 0.05, and the proportion of leading-edge genes accounting for >30% of the entire gene set involved in the pathway.

Figures 7H and 7I show Venn diagrams of the overlap between significantly enriched pathways in the C2 and H libraries, respectively, for each of the three groups. The full list can be found in Table S3. In total, we found 13 pathways that overlapped among the 3 groups, all 13 of which were positively enriched in both VUB13^del18q^ and hESC^WT_*SALL3*KD^ and negatively enriched in VUB13^del18q_*SALL3*OE^ (Figure 7J shows the cell-type-relevant pathways; the whole set can be found in Table S4). Because of the critical role that these pathways play in pluripotency maintenance and differentiation, we analyzed the expression of pluripotency-associated genes, and we found that *NANOG*, *POU5F1*, *LIN28*, *SOX2*, *PODXL*, *SUSD2*, *MYC*, *FOXD3*, and *DPPA3* are overexpressed in VUB13^del18q^, with all but *DPPA3* and *POU5F1* also being overexpressed upon *SALL3*^KD^ and downregulated by transgenic *SALL3* OE in VUB13^del18q^ (Figure S7A).

## Discussion

In this study, we examined the repercussions of the loss of chromosome 18q on the differentiation capacity of hESCs. For this, we used an early-stage differentiation approach to generate lineage-specific cell types representing the three germ layers: neuroectoderm, hepatoblast, and cardiac progenitors. Our *in vitro* lineage commitment studies indicated that the deletion of 18q in hESCs impaired neuroectodermal differentiation and delayed cardiac progenitor differentiation, whereas no consistent differences were observed in the commitment toward hepatoblasts. It is important to bear in mind that we used a set of three or four markers to establish the cell identity after differentiation, precluding an in-depth analysis of the impact of the mutation on the further maturation and functionality of the obtained cells.

To study the mechanistic basis for these changes in differentiation, we looked at the genes located in the minimal region of loss. We found that decreased *SALL3* expression due to the loss of one copy of the gene was sufficient to result in the observed decreased neuroectoderm differentiation, but not to modulate cardiac and hepatoblast differentiation. In this sense, our results are only partially aligned with those obtained by Kuroda et al. (2019). These authors found that downregulating *SALL3* resulted not only in decreased neuroectoderm differentiation, similar to our results, but also in increased cardiac progenitor differentiation. Although we can only speculate about the reasons for these differences, it is likely that the genetic background of the cell lines plays an important role (Kilpinen et al., 2017). Also, given that *SALL3* has been reported to be a modulator of DNMT3A and DNMT3B activity (Kuroda et al., 2019; Shikauchi et al., 2009), the preexisting epigenetic marks in each of the lines, particularly histone modifications, may influence the recruitment of *de novo* methyl transferases to methylate the DNA (Baubec et al., 2015; Weinberg et al., 2019). Furthermore, it is possible that other genes located in 18q, or in the gain regions of chromosomes 5 and 7, cause cell-line-specific effects.

In line with this reasoning, the gene expression analysis revealed that only part of the divergence between hESCs with 18q loss and their chromosomally normal isogenic counterparts was related to the differential expression of *SALL3*. Interestingly, the core set of deregulated genes was associated with pathways involved in the maintenance of and exit from the undifferentiated pluripotent state, and key regulators of pluripotency were both upregulated in hESCs^18q^ and regulated by *SALL3* expression. For instance, transforming growth factor β (TGF-β) signaling is key to the maintenance of the primed pluripotent state (Weinberger et al., 2016). TGF-β and bone morphogenetic protein 4 (BMP4) signaling are also core genes in regulating the balance between neuroectoderm differentiation and mesendoderm in both humans and mice (Park, 2011). Notch activation mediates TGF-β signaling during hESC and mesenchymal stem cell differentiation into smooth muscle cells (Kurpinski et al., 2010), and its inhibition supports naive state consolidation in rodent models (Weinberger et al., 2016). The cytokine tumor necrosis factor α has been shown to negatively regulate the differentiation of various cell types, including cardiomyocytes (Hamid et al., 2016) and embryoid bodies (Wuu et al., 1998). In addition, nuclear factor-κB inhibition has been found to mediate naive pluripotency in mice (Dutta et al., 2011). Overall, these results suggest that downregulation of *SALL3* due to the loss of 18q alters undifferentiated-state maintenance in hESCs by affecting pluripotency-associated pathways, and these changes have profound effects on the differentiation capacity of the cells.

With hPSCs steadily moving into clinical trials (Kobold et al., 2020) and being broadly used as a cell source for *in vitro* modeling of, for instance, developmental processes and diseases, determining the impact of recurrent genetic abnormalities is critical (Andrews et al., 2022; Keller et al., 2018). Work from our group and others is beginning to generate a detailed picture showing how these genetic abnormalities affect differentiation in a cell lineage-specific manner. For instance, 20q11.21 gain impairs neuroectoderm commitment without affecting mesendoderm induction (Jo et al., 2020; Markouli et al., 2019), and recently, it has been shown that cells with an isochromosome 20q are not able to survive retinal pigmented epithelial cell differentiation and display overall disruptions in the ability to correctly differentiate (Vitillo et al., 2023). In this work, we show that 18q loss specifically impairs neuroectoderm commitment and appears to delay cardiac differentiation. Taken together, these results highlight the importance of the genetic screening of hPSC cultures to ensure that these abnormalities do not pass unnoticed. In a research setting, chromosomal abnormalities could lead to confounding effects that decrease the reliability and reproducibility of the work, and in a clinical setting, they could lead at best to decreased therapeutic efficacy and at worst to tumorigenesis (Andrews et al., 2022; Keller et al., 2018; Yamanaka, 2020).

In conclusion, in this study, we have characterized the differentiation capacity of hESCs with 18q loss, one of the recurrent, albeit less common, genetic abnormalities found in hPSC cultures. We found that these cells are characterized by abnormal differentiation into cardiac progenitors and an impaired capacity for neuroectoderm commitment, the latter driven by the loss of one copy of *SALL3*. This gene is an inhibitor of *DNMT3B*, and its downregulation results in changes in the expression of genes involved in the maintenance of pluripotency and in hESC differentiation. Further research will be needed to assess whether other cell-type-specific effects of this abnormality exist and may be revealed by longer differentiation protocols, beyond the progenitor stage, as well as the potential consequences of 18q loss for oncogenic potential.

## Experimental procedures

### Resource availability

#### Lead contact

Further information and requests for resources should be directed to the corresponding author, Claudia Spits (claudia.spits@vub.be).

#### Materials availability

All VUB stem cell lines in this study, including the genetically abnormal sublines and genetically modified lines, are available upon request and after signing a materials transfer agreement.

#### Data and code availability

Raw sequencing data of human samples are considered personal data by the General Data Protection Regulation of the European Union (Regulation (EU) 2016/679), because SNPs can be extracted from the reads and cannot be shared publicly. The data can be obtained from the corresponding author upon reasonable request and after signing a data use agreement. The RNA-seq counts per million tables are provided in the Data S1, which allow for downstream gene expression analysis. The data supporting all of the figures in this paper can be found at the Open Science Framework repository: https://osf.io/hpaxc/.

### Ethics statement

For all parts of this study, the design and conduct complied with all of the relevant regulations regarding the use of human materials, and all of them were approved by the local ethical committee of the University Hospital UZ Brussel and the Vrije Universiteit Brussel (file no. B.U.N. 1432020000284). All of the patients donating embryos to derive hESC lines gave written consent.

### hESC maintenance and passaging

All of the hESC lines in this study were derived in-house in the past. The details on the derivation and results of the characterization, including tests for pluripotency, were reported previously (Mateizel et al., 2006; 2010) and can be also found at the Open Science Framework repository: https://osf.io/esmz8/. The lines are registered in the EU hPSC registry (https://hpscreg.eu/) and available upon request. All of the experiments in this study have been carried out on cells that belong to the same working cell bank, each of which tested for genetic content by shallow whole-genome sequencing and for mycoplasma. No cells were used beyond five passages after being drawn from the working bank.

The hESCs were maintained in NutriStem hESC XF medium (NS medium; Biological Industries) with 100 U/mL penicillin/streptomycin (Thermo Fisher Scientific) in a 37°C incubator with 5% CO_2_ on Biolaminin 521-coated dishes (Biolamina). The culture medium was changed daily. The cells were passaged as single cells using TrypLE Express (Thermo Fisher Scientific) and split when reaching 70%–90% confluence. The medium was supplemented with 10 μM rho kinase inhibitor Y-27632 (Tocris) for the first 24 h after passaging.

### Copy-number variant (CNV) analysis

The genetic content of the hESCs was assessed through shallow whole-genome sequencing by the BRIGHTcore of UZ Brussels, Belgium, as previously described (Bayindir et al., 2015). We also conducted CNV analysis using quantitative real-time PCR at regular intervals, particularly before and after performing lentiviral transduction and starting differentiation. DNA was extracted with a DNeasy Blood and Tissue Kit (Qiagen) according to the manufacturers’ protocol. qPCR was performed with the copy-number assays *RNaseP* (Thermo Fisher Scientific) as a reference and *KIF14*, *NANOG*, *NMT1*, and *ID1* (Thermo Scientific) covering the 1q, 12p, 17q, and 20q regions, respectively. The reaction systems were prepared by mixing TaqMan 2× Mastermix Plus–Low ROX (Eurogentec) and the TaqMan assays together with the DNA samples. qPCR was performed on a ViiA7 thermocycler (Thermo Fisher Scientific), and Applied Biosystems Copy Caller version 2.1 was used to analyze the CNVs.

### Total RNA isolation, cDNA synthesis, and quantitative real-time PCR for gene expression analysis

Total RNA was isolated using RNeasy Mini and Micro kits (Qiagen) following the manufacturer’s guidelines, including on-column DNase I treatment. mRNA was reverse transcribed into biotinylated cDNA using the First-Strand cDNA Synthesis Kit (Cytiva) with the NotI-d(T)18 primer. Quantitative real-time PCR was carried out using TaqMan mRNA expression assays (Thermo Fisher Scientific, listed in Table S5) and TaqMan 2× Mastermix Plus–Low ROX (Eurogentec) on a ViiA 7 thermocycler (Thermo Fisher Scientific) using the standard settings provided by the manufacturer. The relative expression was determined by the comparative Ct method, and *GUSB* was used as the housekeeping gene.

### Immunostaining

Differentiated cells were fixed in a solution of PBS containing 3.7% formaldehyde (Sigma-Aldrich) for 15 min, permeabilized in 0.1% Triton X-100 for 10 min (Sigma-Aldrich) and blocked with 10% fetal bovine serum (FBS; Thermo Fisher Scientific) for 1 h at room temperature (RT). Primary antibodies were diluted in a 10% FBS (Thermo Fisher Scientific) blocking solution and incubated overnight at 4°C. Alexa 488 or Alexa 594 conjugated secondary antibodies (diluted in 10% FBS) and Hoescht (1:1,000 dilution, ThermoFisher Scientific) were applied for 1–2 h at RT. Confocal images were acquired with an LSM800 confocal microscope (Carl Zeiss). For quantification, the positive cells were counted and compared to the number of Hoescht-stained nuclei to determine the percent positivity using ZEN 2 (blue edition) imaging software. The areas are randomly selected based on the Hoescht channel, and the positive cells are quantified by calculating the ratio of total positive cells to the total number of cells within those areas. The numbers of cells quantified for each image can be found in Table S6. The lists with antibodies can be found in Table S7.

### *In vitro* differentiation of iPSCs

The neuroectoderm differentiation was based on Douvaras and Fossati (2015). In brief, 90% confluent hESCs were subjected to neural induction for 8 days using 100 nM RA (Sigma-Aldrich), 10 μM SB431542 (Tocris), and 250 nM LDN193189 (STEMCELL Technologies). The induction of cardiac progenitor differentiation was based on Lin and Zou (2020). hESCs at 80%–90% confluence were treated with 5 mM CHIR99021 (Tocris) for 24 h, after which the cells were cultured in medium with 0.6 U/mL heparin (Sigma-Aldrich) for 24 h. Subsequently, the medium was supplemented with 0.6 U/mL heparin and 3 mM IWP2 (Tocris) for another 3 days. The 8-day depatoblast differentiation was based on Boon et al. (2020). Differentiation was initiated at 40%–50% confluency, and the hESCs were treated with with 50 ng/mL activin A (STEMCELL Technologies), 50 ng/mL WNT3A (PeproTech), and 6 μL/mL DMSO (Sigma-Aldrich) for 48 h. The cells were incubated for an additional 48 h in the same medium without WNT3A. Then, the medium was changed to contain 50 ng/mL BMP4 (STEMCELL Technologies) and 6 μL/mL DMSO for the following 4 days. More details on the differentiation and the media composition can be found in the supplemental information.

### Generation of *SALL3* KD and OE cell lines

hESC^WT_*SALL3*KD^ cells were generated by infecting hESC^WT^ with lentiviral particles expressing *SALL3*-targeted shRNAs (SigmaMISSION shRNA targeting set TRCN0000019754, TRCN0000417790) or control shRNA plasmid. hESC^del18q_*SALL3*OE^ cells were generated by infecting hESC^del18q^ with lentiviral particles containing the pLVSIN-EF1α puromycin vector expressing *SALL3* (a gift from Yoji Sato, Division of Cell-Based Therapeutic Products, National Institute of Health Sciences, Japan).

### RNA-seq

RNA-seq library preparation was performed using QuantSeq 3′ mRNA-Seq Library Prep Kits (Lexogen) following Illumina protocols. Sequencing was performed on a high-throughput Illumina NextSeq 500 flow cell. On average, 13.9 × 10^6^ ± 7.1 × 10^6^ paired-end reads per sample were uniquely mapped, with an average coverage per base of 101 paired reads. Details on the bioinformatic processing can be found in the supplemental information.

### Statistics

All of the differentiation experiments were carried out in at least triplicate (n ≥ 3). All of the data are presented as the mean ± SEM. Statistical evaluation of the differences between two groups was performed using unpaired two-tailed t tests in GraphPad Prism9 software, with p < 0.05 determined to indicate significance.

## Supplemental information

Document S1. Figures S1–S7, Tables S1 and S5–S7, and Supplemental methods

Table S2. Differentially expressed genes identified by RNA sequencing

Table S3. All enriched pathways identified by GSEA

Table S4. Commonly enriched pathways, source data to Figure 3J

Data S1. CPM table RNA sequencing

Document S2. Article plus supplemental information
