# Supplementary material for: SALL3 mediates the loss of neuroectodermal differentiation potential in human embryonic stem cells with chromosome 18q loss
Source: Stem Cell Reports. 2024 Mar 28;19(4):562–78. doi: 10.1016/j.stemcr.2024.03.001 (PMC11096619; doi:10.1016/j.stemcr.2024.03.001)
Supplement: Document S1. Figures S1–S7, Tables S1 and S5–S7, and Supplemental methods [file mmc1.pdf]

Stem Cell Reports, Volume *19*

Supplemental Information

SALL3 mediates the loss of neuroectodermal differentiation potential in human embryonic stem cells with chromosome 18q loss

Yingnan Lei, Diana Al Delbany, Nuša Krivec, Marius Regin, Edouard Couvreu de Deckersberg, Charlotte Janssens, Manjusha Ghosh, Karen Sermon, and Claudia Spits

Supplementary table 1. Passage range and karyotype of the lines in this study

Line	Passage range	Karyotype	Method of karyotyping and size in Mb
VUB14^{wt}	51-55	46, XX	Shallow whole genome sequencing
VUB04^{wt}	16-23	46, XX	Shallow whole genome sequencing
VUB03^{wt}	19-25	46, XX	Shallow whole genome sequencing
VUB13^{del18q}	52-58	46, XX<br>dup(5) (q21.3qter)<br>del(18) (q21.2qter)	Shallow whole genome sequencing<br>chr5: 33 Mb<br>chr18: 29 Mb
VUB14^{del18q}	49-54	46, XX,<br>dup(7)(p22.3pter)<br>del(18)(q21.32qter)	Shallow whole genome sequencing<br>chr7: 32 Mb<br>chr18: 21 Mb
VUB04	29	46, XX	Array-based comparative genomic hybridization (published in Spits et al., 2008)
	66	46, XX,<br>dup(5)(q14.2qter),<br>del(18)(q21.2qter)	Array-based comparative genomic hybridization (published in Spits et al., 2008)<br>Chr5: 99.5 Mb<br>Chr18: 28.7 Mb
VUB26	10	46, XX,<br>dup(7)(q33qter),<br>del(18)(q23qter)	Array-based comparative genomic hybridization (published in Spits et al., 2008)<br>Chr7: 11.3 Mb<br>Chr18: 4.6 Mb

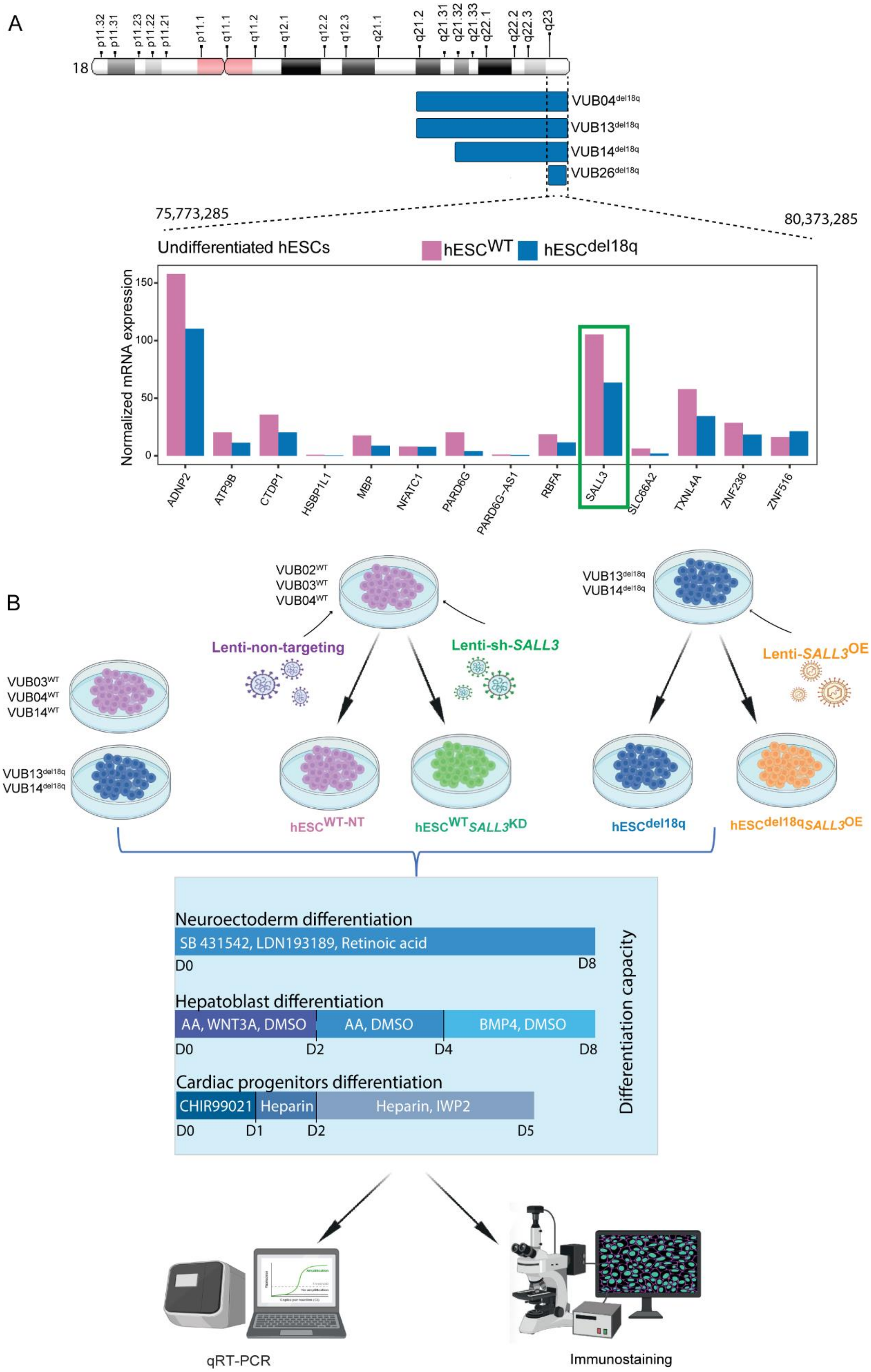
A
18
p11.32
p11.31
p11.23
p11.22
p11.21
p11.1
q11.1
q11.2
q12.1
q12.2
q12.3
q21.1
q21.2
q21.31
q21.32
q21.33
q22.1
q22.2
q22.3
q23
VUB04del18q
VUB13del18q
VUB14del18q
VUB26del18q
75,773,285
80,373,285
Undifferentiated hESCs
hESCWT
hESCdel18q
Normalized mRNA expression
150
100
50
0
ADNP2
ATP9B
CTDP1
HSBP1L1
MBP
NFATC1
PARD6G
PARD6G-AS1
RBFA
SALL3
SLC66A2
TXNL4A
ZNF236
ZNF516
B
VUB02WT
VUB03WT
VUB04WT
VUB13del18q
VUB14del18q
Lenti-non-targeting
Lenti-sh-SALL3
Lenti-SALL3OE
VUB03WT
VUB04WT
VUB14WT
VUB13del18q
VUB14del18q
hESCWT-NT
hESCWTSALL3KD
hESCdel18q
hESCdel18qSALL3OE
Neuroectoderm differentiation
SB 431542, LDN193189, Retinoic acid
D0
D8
Hepatoblast differentiation
AA, WNT3A, DMSO
AA, DMSO
BMP4, DMSO
D0
D2
D4
D8
Cardiac progenitors differentiation
CHIR99021
Heparin
Heparin, IWP2
D0
D1
D2
D5
Differentiation capacity
qRT-PCR
Immunostaining

Supplementary figure 1. The minimal common region of 18q loss spans 14 genes expressed in undifferentiated hESCs, including *SALL3*. (A) Graphical representation of the regions of chromosome 18 loss in all VUB hESC^{del18q} lines and showing the normalized expression of the genes located in the minimal common region of 18q loss examined by bulk-RNA sequencing to compare the transcriptome of hESC^{del18q} and hESC^{WT} as well as the genetically modified counterparts. (B) Overview of the experimental setup. We used two lines carrying a loss of 18q and three genetically balanced lines. The WT lines were also genetically modified to down-regulate *SALL3*, while 18q loss lines were modified to over-express *SALL3*. All lines were subjected to differentiate to neuroectoderm, hepatoblasts and cardiac progenitors, all differentiation experiments were carried out at least in triplicate. qRT-PCR and immunostaining were used to assess the identity of the differentiated cells. AA: Activin A; Lenti-sh-*SALL3*: lentiviral transduction for a short-hairpin RNA against *SALL3.* Lenti-*SALL3*-OE: lentiviral transduction with a construct for the transgenic over-expression of *SALL3.*

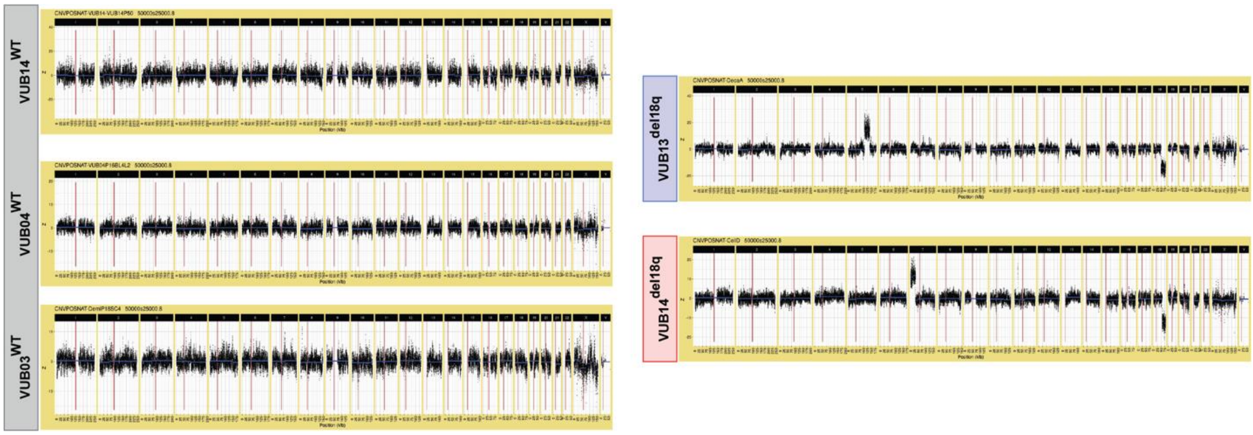

Supplementary figure 2. Shallow sequencing results of the lines used in this study

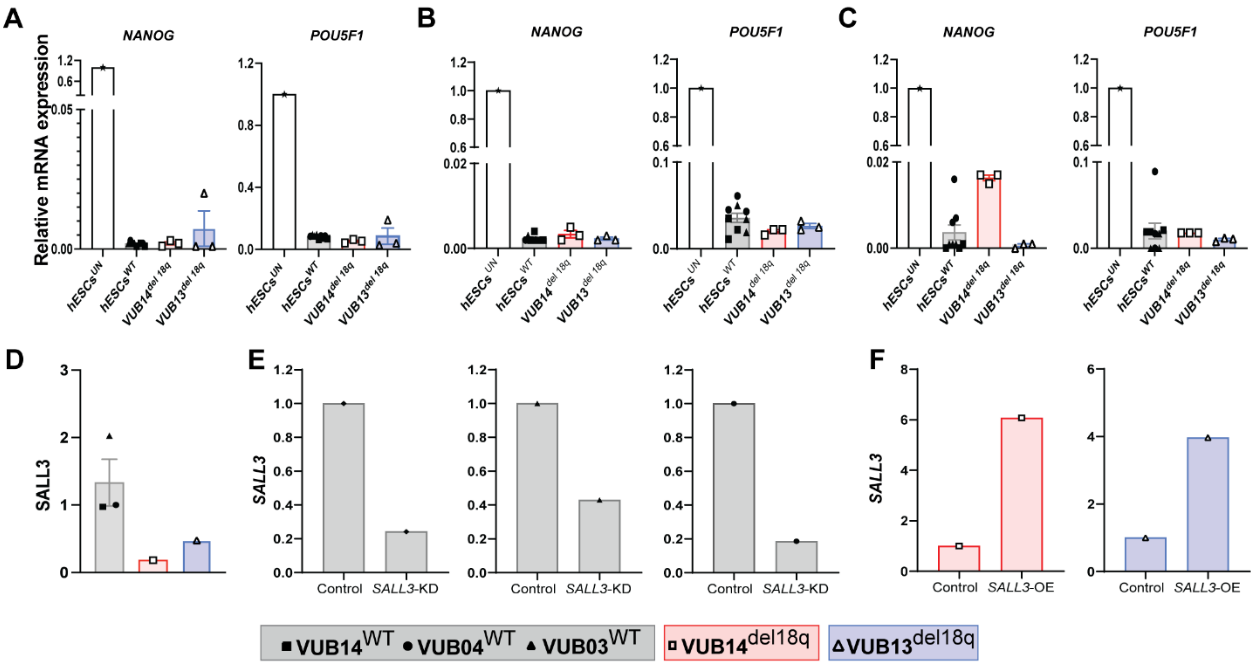

Supplementary figure 3. The relative mRNA expression of undifferentiated state markers during the differentiation (A-C) **and the relative *SALL3* mRNA expression** (D-F). Relative mRNA expression as measured by qRT-PCR for pluripotency markers *NANOG*, *POU5F1* during the differentiation process (n = 3). Data are shown as means ± SEM, each pattern indicates different cell lines. Neuroectoderm (A) cardiac progenitor (B) hepatoblasts (C). Relative *SALL3* mRNA expression as measured by qRT-PCR between hESC^{del18q} compared to hESC^{WT} (D). Relative *SALL3* mRNA expression as measured by qRT-PCR between hESC$^{WT\text{-}NT}$ and hESC^{WT_SALL3KD} (E). Relative *SALL3* mRNA expression as measured by qRT-PCR between hESC^{del18q} and hESC^{del18q_SALL3OE} (F)

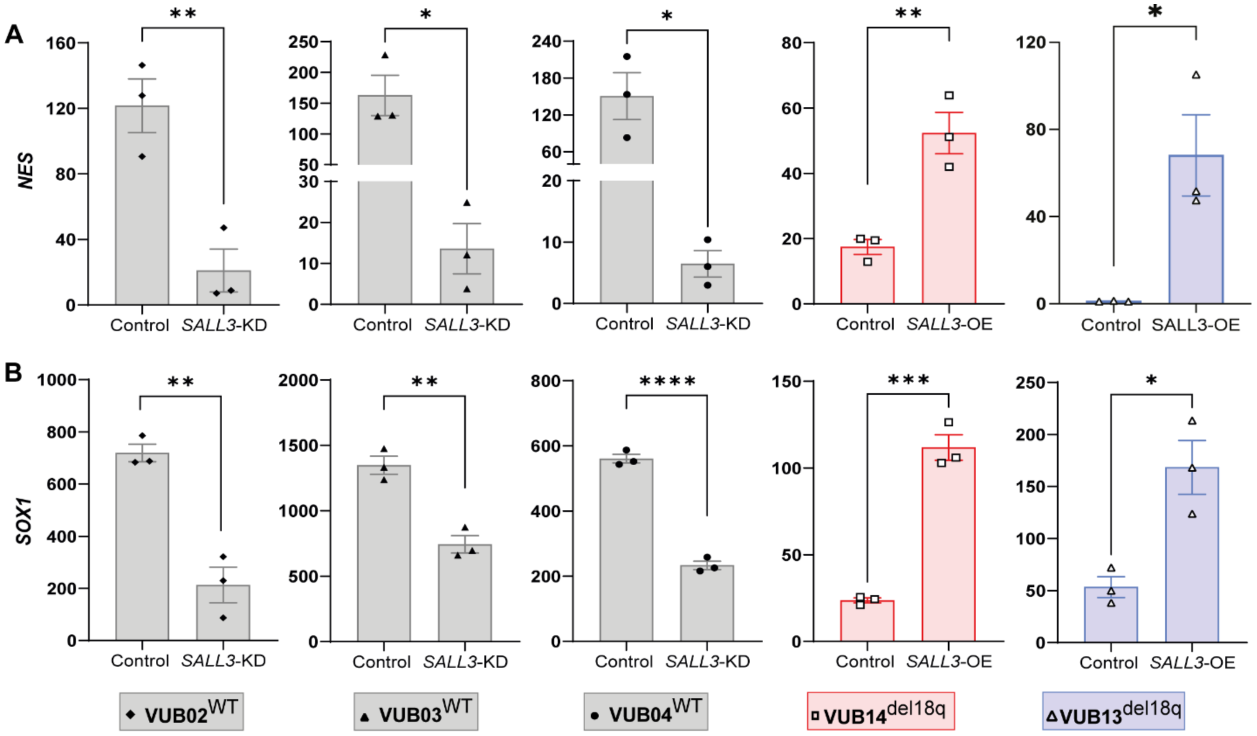

Supplementary Figure 4. *SALL3* drives the impaired neuroectoderm differentiation (A-B) Relative mRNA expression for neuroectoderm markers (n = 3) by qRT-PCR. Different patterns indicate different cell lines, and the horizontal bars with asterisks *, **, *** and **** represent statistical significance between samples at 5%, 1%, 0.1% and 0.01% respectively (unpaired t-test). *NES* (A), *SOX1*(B).

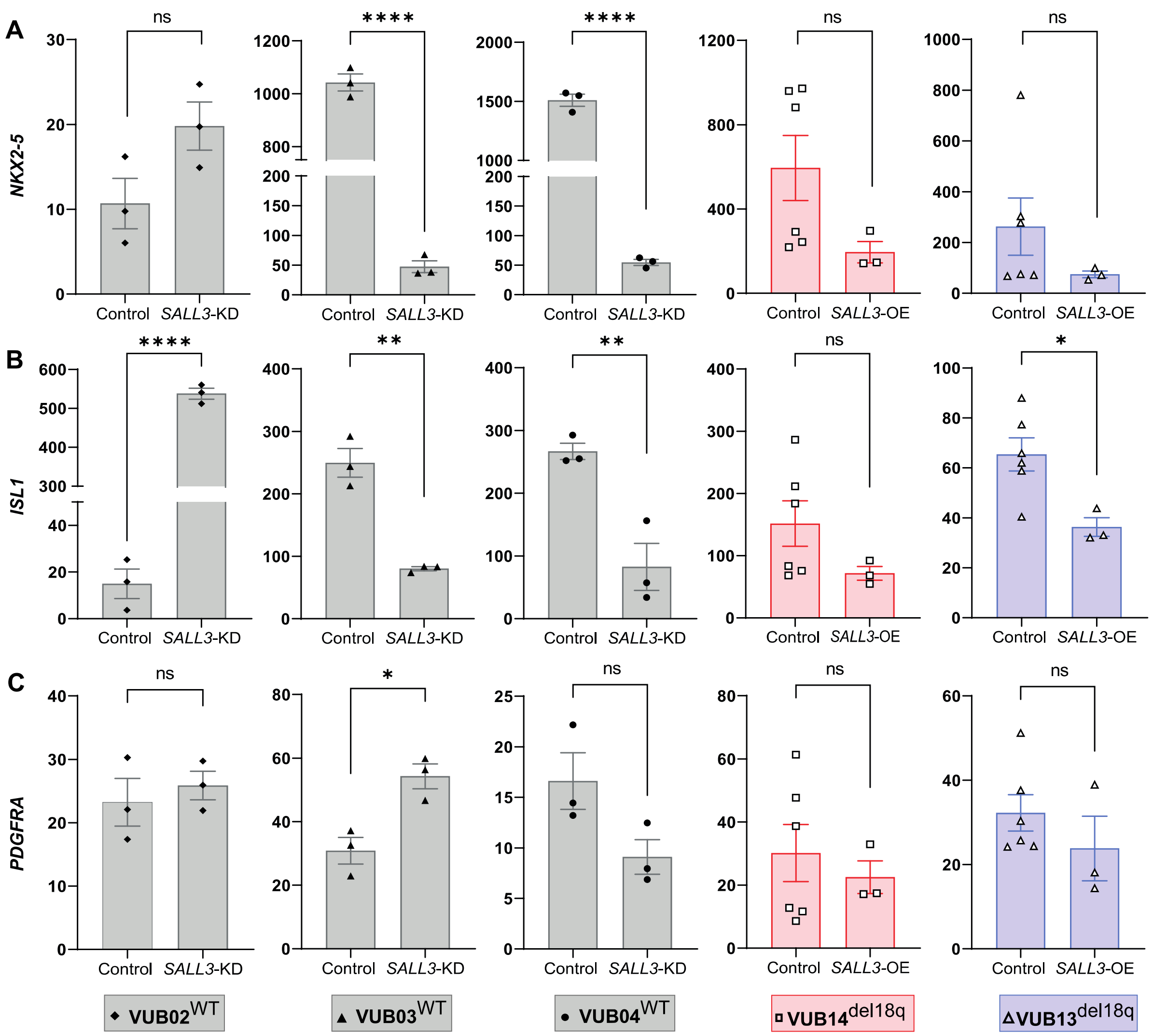

Supplementary Figure 5. *SALL3* is not affecting the cells ability to generate mesoderm (A-C) Relative mRNA expression for mesoderm markers measured by qRT-PCR (n = 3). Data are shown as the means ± SEM. Different patterns indicate different cell lines, and the horizontal bars with asterisks *, **, *** and **** represent statistical significance between samples at 5%, 1%, 0.1% and 0.01% respectively (unpaired t-test). *NKX2-5*(A), *ISL1*(B), *PDGFRA*(C).

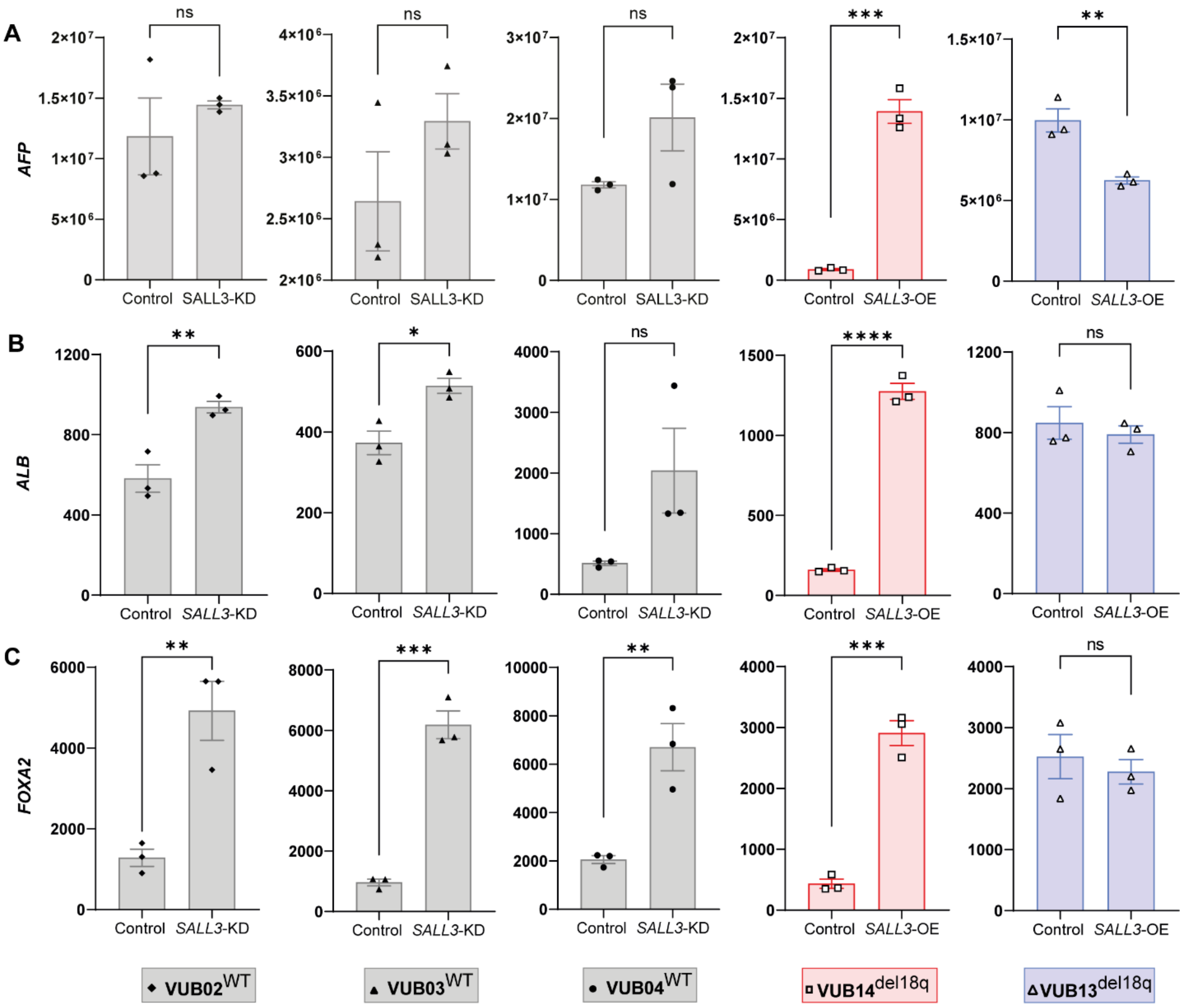

Supplementary Figure 6. *SALL3* is affecting the cells ability to generate endoderm (A-C) Relative mRNA expression for endoderm markers (n = 3) by qRT-PCR. Data are shown as the means ± SEM. Different patterns indicate different cell lines, and the horizontal bars with asterisks *, **, *** and **** represent statistical significance between samples at 5%, 1%, 0.1% and 0.01% respectively (unpaired t-test). *AFP*(A), *ALB*(B), *FOXA2*(C).

A

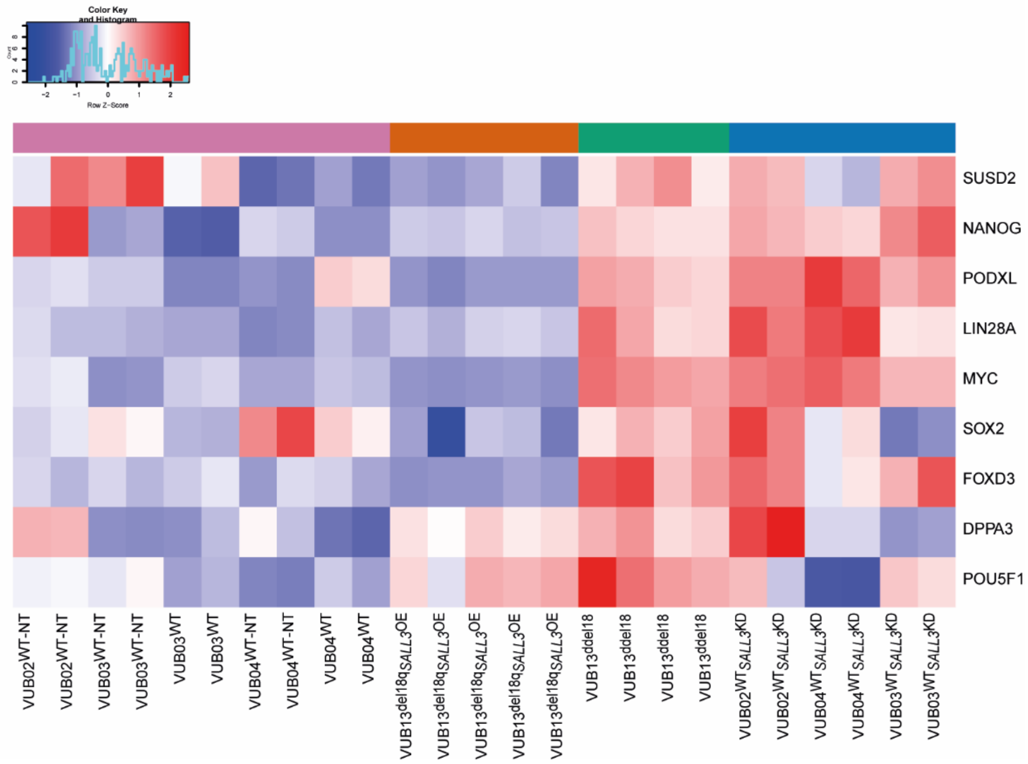

Supplementary Figure 7. Global transcriptomic profile analysis (A) Heatmap of pluripotency-associated genes

Supplementary methods

Definitive neuroectoderm specification

The protocol for inducing neuroectoderm differentiation was adapted from the protocol described by Douvaras and colleagues(Douvaras and Fossati, 2015). In brief, hESCs were seeded on Biolaminin 521 (Biolamina®)-coated 24-well plates at a ratio of 100,000 cells per cm^2 and grown to 90% confluence. Then, differentiation was induced by incubating the hESCs with neural induction specification medium for up to 8 days with daily medium changes. The neural induction specification medium consisted of basal medium supplemented with the following differentiation factors: 100 nM retinoic acid (Sigma-Aldrich), 10 µM SB431542 (Tocris), and 250 nM LDN193189 (STEMCELL Technologies). The basal medium was prepared by mixing DMEM/F12 (Thermo Scientific) basic medium supplemented with 1x NEAA (Thermo Scientific), 1x GlutaMAX (Thermo Scientific), 1x 2-mercaptoethanol (Thermo Scientific), 25 µg/mL insulin (Sigma-Aldrich) and 1x penicillin/streptomycin (P/S) (Thermo Scientific).

Cardiac progenitor differentiation

The induction of cardiac progenitor differentiation was initiated using a slightly modified version of the protocol from a previous publication(Lin and Zou, 2020). hESCs were seeded on Biolaminin-521-coated 24-well plates at a density of 100,000 cells/cm^2 and allowed to reach 80–90% confluence. At this point, differentiation was initiated by treating the hESCs with cardiomyocyte differentiation basal medium (CDBM) with 5 mM CHIR99021 (Tocris) to activate Wnt/β-catenin signaling and form the mesendoderm layer. After 24 h, CHIR99021 was removed, and the cells were cultured in CDBM with 0.6 U/mL heparin (Sigma-Aldrich) for 24 h. Subsequently, the CDBM medium was supplemented with 0.6 U/ml heparin and 3 mM IWP2 (Tocris) for another 3-day incubation with medium refreshed daily. The cardiomyocyte differentiation basal medium (CDBM) was composed of 10 µg/ml transferrin (Sigma Aldrich), 1x chemically defined lipid concentrate (Thermo Scientific) and E8 basal medium. The E8 medium was composed of DMEM/F12 (Thermo Scientific) basic medium with 64 mg/L L-ascorbic acid (Sigma-Aldrich) and 13.6 µg/L sodium selenium (Sigma-Aldrich).

Hepatoblast differentiation

Differentiation of hESCs into hepatoblasts was conducted using a protocol based on(Boon et al., 2020). The hESCs were seeded at a density of $5x10^4$ cells/cm^2 on 24-well plates precoated with Biolaminin-521. Upon reaching approximately 40-50% confluency, the hESCs were treated with liver differentiation medium (LDM) along with 50 ng/mL Activin A (STEMCELL Technologies), 50 ng/mL WNT3A (PeproTech) and 6 µL/mL DMSO (Sigma-Aldrich) for 48 h. The cells were incubated for an additional 48 h in the same medium without WNT3A. Then, the medium was changed to LDM with 50 ng/mL BMP4 (STEMCELL Technologies) and 6 µL/mL DMSO for the following 4 days, with the medium changed every two days. The samples were collected on day 8. LDM medium was created by adding MCDB 201 medium (pH=7.2, Sigma-Aldrich), L-ascorbic acid (Sigma-Aldrich), insulin-transferrin-selenium (ITS-G, Thermo Scientific),

linoleic acid-albumin from bovine serum albumin (LA-BSA, Sigma-Aldrich), 2-mercaptoethanol (Thermo scientific), and dexamethasone (Sigma-Aldrich) to DMEM high glucose medium (Westburg Life Sciences).

RNA sequencing

The FastQC algorithm(Love et al., 2014) was used to perform quality control on the raw sequence reads prior to the downstream analysis. The raw reads were aligned to the new version of the human Ensembl reference genome (GRCh38.p13) with Ensembl (GRCh38.83gtf) annotation using STAR version 2.5.3 in 2-pass mode(Dobin et al., 2013).The aligned reads were then quantified, and transcript abundances were estimated using RNA-seq by expectation maximization (RSEM, version 1.3.3)(Li and Dewey, 2011).

The count matrices were imported into R software (version 3.3.2) for further processing. The edgeR(Robinson et al., 2010) package was utilized to identify differentially expressed genes (DEGs) between groups. Transcripts with a count per million (cpm) greater than 1 in at least two samples were considered for the downstream analysis. Genes with a log2-fold change greater than 1 or less than -1 and a false discovery rate (FDR)-adjusted P-value less than 0.05 were considered significantly differentially expressed. Volcano plots of DEGs were generated using the ggplot2(Wickham, 2016) package in R, while Venn diagrams using VennDiagram function were used to visualize the overlap of the DEGs among different individuals. Principal component analysis (PCA) and heatmap clustering were performed using normalized counts and R packages. The heatmap was generated using the heatmap.2 funtion. PCA was performed using the prcomp function and plotted using ggplot2. Gene set enrichment analysis (GSEA) was applied to detect the enrichment of pathways using the GSEA function in R with the MSigDB C2 and H databases. Values are ranked by sign(logFC)*(-log10(FDR)). $|NES| > 1$ and p-value < 0.05 were considered the thresholds for significance for the gene sets.

References

Boon, R., Kumar, M., Tricot, T., Elia, I., Ordovas, L., Jacobs, F., One, J., De Smedt, J., Eelen, G., Bird, M., et al. (2020). Amino acid levels determine metabolism and CYP450 function of hepatocytes and hepatoma cell lines. Nature Communications 2020 11:1 *11*, 1–16. https://doi.org/10.1038/s41467-020-15058-6.

Dobin, A., Davis, C.A., Schlesinger, F., Drenkow, J., Zaleski, C., Jha, S., Batut, P., Chaisson, M., and Gingeras, T.R. (2013). STAR: ultrafast universal RNA-seq aligner. Bioinformatics *29*, 15. https://doi.org/10.1093/BIOINFORMATICS/BTS635.

Douvaras, P., and Fossati, V. (2015). Generation and isolation of oligodendrocyte progenitor cells from human pluripotent stem cells. Nature Protocols 2015 10:8 *10*, 1143–1154. https://doi.org/10.1038/nprot.2015.075.

Li, B., and Dewey, C.N. (2011). RSEM: Accurate transcript quantification from RNA-Seq data with or without a reference genome. BMC Bioinformatics *12*, 1–16. https://doi.org/10.1186/1471-2105-12-323/TABLES/6.

Lin, Y., and Zou, J. (2020). Differentiation of Cardiomyocytes from Human Pluripotent Stem Cells in Fully Chemically Defined Conditions. STAR Protoc *1*. https://doi.org/10.1016/J.XPRO.2020.100015.

Love, M.I., Huber, W., and Anders, S. (2014). Moderated estimation of fold change and dispersion for RNA-seq data with DESeq2. Genome Biol *15*, 1–21. https://doi.org/10.1186/S13059-014-0550-8/FIGURES/9.

Robinson, M.D., McCarthy, D.J., and Smyth, G.K. (2010). edgeR: a Bioconductor package for differential expression analysis of digital gene expression data. Bioinformatics *26*, 139–140. https://doi.org/10.1093/BIOINFORMATICS/BTP616.

Wickham, H. (2016). ggplot2. https://doi.org/10.1007/978-3-319-24277-4.

Supplementary table 5

TaqMan Gene Expression Assays

Gene	Supplier	Catalog number
GUSB	Applied Biosystems™	4326320E
PAX6	Thermo Scientific	Hs0024087
SOX1	Thermo Scientific	Hs01057642
NES	Thermo Scientific	Hs04187831
AFP	Thermo Scientific	Hs00173490
HNF4A	Thermo Scientific	Hs00604435
FOXA2	Thermo Scientific	Hs00232764
ALB	Thermo Scientific	Hs00609411
GATA4	Thermo Scientific	Hs01034629
NKX2-5	Thermo Scientific	Hs00231763
PDGFRA	Thermo Scientific	Hs00998018
ISL1	Thermo Scientific	Hs00158126
POU5F1	Thermo Scientific	Hs00742896
NANOG	Thermo Scientific	Hs02387400

TaqMan Copy Number Variation Assay

Gene	Supplier	Catalog number	Chromosome
RNaseP	Thermo Scientific	4403326	reference
ID1	Thermo Scientific	Hs01892845	20q
KIF14	Thermo Scientific	Hs00637799	1q
NANOG	Thermo Scientific	Hs03820140	12p
NMT1	Thermo Scientific	Hs0550678	17q

Supplementary table 6. Numbers of cells quantified for each image.

**Figure 1, Neuroectoderm**	Each datapoint shown in 1C represents the average of the quantification of 3 randomly taken images of the same well: $VUB14^{wt}$ N= 21907 cells, $VUB04^{wt}$ N= 22399 cells, $VUB03^{wt}$ N= 22696 cells, $VUB14^{del18q}$ N= 10882 cells, $VUB13^{18qdel}$ N= 13462 cells.
**Figure 2, Cardiac progenitors**	Each datapoint shown in 2C represents the average of the quantification of 5 randomly taken images of the same well: $VUB14^{wt}$ N= 25513 cells, $VUB04^{wt}$ N= 27758 cells, $VUB03^{wt}$ N= 24031 cells, $VUB14^{del18q}$ N= 25348 cells, $VUB13^{18qdel}$ N= 23857 cells.
**Figure 3, Hepatoblasts**	Each datapoint shown in 3C represents the average of the quantification of 5 randomly taken images of the same well: $VUB14^{wt}$ N= 15325 cells, $VUB04^{wt}$ N= 12171 cells, $VUB03^{wt}$ N= 11816 cells, $VUB14^{del18q}$ N= 21692 cells, $VUB13^{del18q}$ N= 17302 cells
**Figure 4, Neuroectoderm**	Each datapoint shown in 4C represents the average of the quantification of 3 randomly taken images of the same well: $VUB04^{WT\text{-}NT}$ N= 23280 cells, $VUB04^{WT\_\mathit{SALL3}KD}$ N= 16441 cells, $VUB03^{WT\text{-}NT}$ N= 21189 cells, $VUB03^{WT\_\mathit{SALL3}KD}$ N= 20844 cells, $VUB02^{WT\text{-}NT}$ N= 23952 cells, $VUB02^{WT\_\mathit{SALL3}KD}$ N= 13023 cells, $VUB14^{del18q}$ N= 27466 cells, $VUB14^{del18q\_\mathit{SALL3}OE}$ N= 49709 cells, $VUB13^{del18q}$ N= 39424 cells, $VUB13^{del18q\_\mathit{SALL3}OE}$ N= 48155 cells.
**Figure 5, Cardiac progenitors**	Each datapoint shown in 5C represents the average of the quantification of 5 randomly taken images of the same well: $VUB04^{WT\text{-}NT}$ N= 15015 cells, $VUB04^{WT\_\mathit{SALL3}KD}$ N= 14997 cells, $VUB03^{WT\text{-}NT}$ N= 23719 cells, $VUB03^{WT\_\mathit{SALL3}KD}$ N= 13114 cells, $VUB02^{WT\text{-}NT}$ N= 14659 cells, $VUB02^{WT\_\mathit{SALL3}KD}$ N= 12089 cells, $VUB14^{del18q}$ N= 23652 cells, $VUB14^{del18q\_\mathit{SALL3}OE}$ N= 23809 cells, $VUB13^{del18q}$ N= 22286 cells, $VUB13^{del18q\_\mathit{SALL3}OE}$ N= 17318 cells.
**Figure 6, Hepatoblasts**	Each datapoint shown in 6C represents the average of the quantification of 5 randomly taken images of the same well: $VUB04^{WT\text{-}NT}$ N= 15703 cells, $VUB04^{WT\_\mathit{SALL3}KD}$ N= 12316 cells, $VUB03^{WT\text{-}NT}$ N= 28766 cells, $VUB03^{WT\_\mathit{SALL3}KD}$ N= 17809 cells, $VUB02^{WT\text{-}NT}$ N= 20361 cells, $VUB02^{WT\_\mathit{SALL3}KD}$ N= 17414 cells, $VUB14^{del18q}$ N= 22061 cells, $VUB14^{del18q\_\mathit{SALL3}OE}$ N= 18296 cells, $VUB13^{del18q}$ N= 17302 cells, $VUB13^{del18q\_\mathit{SALL3}OE}$ N= 16282 cells.

Supplementary table 7

Primary Antibodies

Gene	Species	Supplier	Catalog number	Dilution
PAX6	Mouse	Abcam	ab78545	1:200
HNF4A	Mouse	Santa Cruz Biotechnology	Sc-374229	1:200
GATA4	Rabbit	Cell Signaling Technology	36966S	1:200
POU5F1	Rabbit	Cell Signaling Technology	2840S	1:400
POU5F1	Mouse	Santa Cruz Biotechnology	sc-5279	1:200

Secondary Antibodies

Gene	Conjugate	Supplier	Catalog number	Dilution
Donkey anti Mouse	Alexa Fluor 488	Thermo Fischer Scientific	A-21202	1:200
Donkey anti Mouse	Alexa Fluor 594	Thermo Fischer Scientific	A-21203	1:200
Donkey anti Rabbit	Alexa Fluor 488	Thermo Fischer Scientific	A-21206	1:200
Donkey anti Rabbit	Alexa Fluor 594	Thermo Fischer Scientific	A-21207	1:200
Hoechst 33342		Invitrogen	H3570	1:2000
