# Supplementary material for: SALL3 mediates the loss of neuroectodermal differentiation potential in human embryonic stem cells with chromosome 18q loss
Source: Stem Cell Reports. 2024 Mar 28;19(4):562–78. doi: 10.1016/j.stemcr.2024.03.001 (PMC11096619; doi:10.1016/j.stemcr.2024.03.001)
Supplement: Document S2. Article plus supplemental information [file mmc6.pdf]

Stem Cell Reports
Article
OPEN ACCESS

SALL3 mediates the loss of neuroectodermal differentiation potential in human embryonic stem cells with chromosome 18q loss

Yingnan Lei,[1] Diana Al Delbany,[1] Nuša Krivec,[1] Marius Regin,[1] Edouard Couvreu de Deckersberg,[1] Charlotte Janssens,[1] Manjusha Ghosh,[1] Karen Sermon,[1] and Claudia Spits[1,2,*]
[1]Research Group Reproduction and Genetics, Faculty of Medicine and Pharmacy, Vrije Universiteit Brussel, Brussels, Laarbeeklaan 103, 1090 Brussels, Belgium
[2]Lead contact
*Correspondence: claudia.spits@vub.be
https://doi.org/10.1016/j.stemcr.2024.03.001

SUMMARY

Human pluripotent stem cell (hPSC) cultures are prone to genetic drift, because cells that have acquired specific genetic abnormalities experience a selective advantage *in vitro*. These abnormalities are highly recurrent in hPSC lines worldwide, but their functional consequences in differentiating cells are scarcely described. In this work, we show that the loss of chromosome 18q impairs neuroectoderm commitment and that downregulation of *SALL3*, a gene located in the common 18q loss region, is responsible for this failed neuroectodermal differentiation. Knockdown of *SALL3* in control lines impaired differentiation in a manner similar to the loss of 18q, and transgenic overexpression of *SALL3* in hESCs with 18q loss rescued the differentiation capacity of the cells. Finally, we show that loss of 18q and downregulation of *SALL3* leads to changes in the expression of genes involved in pathways regulating pluripotency and differentiation, suggesting that these cells are in an altered state of pluripotency.

INTRODUCTION

Human pluripotent stem cells (hPSCs), including human embryonic stem cells (hESCs) and induced pluripotent stem cells (hiPSCs), are self-renewing cells that can give rise to any cell type originating from any of the three embryonic germ layers. This makes hPSCs an attractive resource for *in vitro* disease modeling, developmental biology research, drug discovery, and cell transplantation therapy. A substantial number of clinical trials are under way using hPSC-derived cell products, including for the treatment of age-related macular degeneration, spinal cord injury, and type 1 diabetes (Kobold et al., 2020; Yamanaka, 2020). An important hurdle for both safe clinical translation and the reliable use of hPSCs as *in vitro* research models is the occurrence of cell culture drift due to the acquisition of genetic abnormalities (Andrews et al., 2022). A subset of these genetic aberrations is highly recurrent, and these aberrations are found in hPSC lines worldwide. These recurrent changes vary in size from single-nucleotide point mutations to large chromosome structural variants, the most common being gains of chromosomes 1q, 12p, 17, 20, and X and losses of 10p, 18q and 22p, as well as mutations in *TP53* (Amps et al., 2011; Merkle et al., 2017, 2022). Other genetic changes include epigenetic variations, including erosion of X chromosome inactivation (Bar and Benvenisty, 2019; Geens et al., 2016), mutations in the mitochondrial genome (Van Haute et al., 2013; Zambelli et al., 2018), and an array of other point mutations and structural variants spread throughout the genome (Avior et al., 2019; Merkle et al., 2022).

These recurrent genetic changes arise from the pool of common variations in hPSC cultures via different cell competition mechanisms. hPSCs are prone to replication stress, leading to DNA damage, which in turn is a source of *de novo* genetic variation (Halliwell et al., 2020; Jacobs et al., 2014). For instance, up to 20% of cells in an hESC culture carry *de novo* structural variants, but only a minority of them have the potential to confer a selective advantage to the cells (Jacobs et al., 2014, 2016; Keller et al., 2019), leading to the mutant cells rapidly outcompeting their genetically balanced counterparts (Avery et al., 2013; Nguyen et al., 2014; Olariu et al., 2010; Price et al., 2021). The exact traits that the different chromosomal abnormalities confer on undifferentiated cells, as well as the specific driver genes of these traits, are only well established for the gain of 20q11.21 (Spits et al., 2008). This abnormality confers a decreased sensitivity to apoptosis-inducing events due to increased expression of the gene *BCL2L1*, located in the minimal gained region (Amps et al., 2011; Avery et al., 2013; Nguyen et al., 2014). For gains of 12p, it is thought that *NANOG* drives at least part of the growth advantage of the cells (Ben-David et al., 2014), and cells with a complex karyotype carrying all of the most common abnormalities (gains of 1, 12, 17 and 20q) can outcompete the other cells by corralling and mechanical compression (Price et al., 2021).

An important concern about these genetic variants is whether and how they alter the differentiation capacity of hPSCs and potentially prime differentiated cells for malignant transformation (Andrews et al., 2022; Keller et al., 2018). The gain of 1q is common in cancers, particularly lung adenocarcinoma, breast invasive carcinoma, and liver

© 2024 The Author(s).
This is an open access article under the CC BY license (http://creativecommons.org/licenses/by/4.0/).

hepatocellular carcinoma (Taylor et al., 2018), and appears to confer a growth advantage to cells during differentiation from hESCs to neural precursors (Varela et al., 2012). Moreover, variants in 1q21.1 can alter neurodevelopmental trajectories upon hiPSC differentiation, with the deletion of 1q21.1 accelerating neuronal production and its duplication delaying the transition from neural progenitor cell to neuron (Chapman et al., 2022). Gains in chromosome 12 are frequently found in testicular germ cell tumors (Baker et al., 2007). hPSCs with trisomy 12 display a reduced tendency toward spontaneous differentiation (Ben-David et al., 2014). The highly recurrent gain of 20q11.21 impairs the neuroectodermal lineage commitment of hPSCs (Jo et al., 2020; Markouli et al., 2019), and hESCs with 20q11.1q11.2 amplification have a reduced propensity to differentiate down the hematopoietic lineage, maintain more immature phenotypes along the neural differentiation trajectory, and generate teratomas with foci of undifferentiated cells (Werbowetski-Ogilvie et al., 2009). The gain of chromosome 17 is common in neuroblastomas, testicular germ cell tumors, and breast cancers (Baker et al., 2007), and hPSC lines with a gain of chromosome 17 show altered differentiation patterns in embryoid bodies (Fazeli et al., 2011).

Deletions of chromosome 18q are one of the rarer recurrent structural chromosomal abnormalities in hPSCs. This deletion was first reported as a single event by Maitra et al. in 2005 (Maitra et al., 2005), and our group later found 18q deletions in three different hESC lines at relatively early passages, always as part of a derivative chromosome 18 (Spits et al., 2008). A large study by Amps et al. in 2011 revealed 5 instances of this deletion in hESC (Amps et al., 2011), and WiCell reported that 4% of the 7,300 hPSC cultures evaluated over nearly 8 years carried an 18q deletion (WiCell Cytogenetics Lab, https://www.wicell.org/media.acux/29102c0e-e88e-426b-ab7d-bac4c2a9ec6a). Chromosome 18q loss is common in cancers, especially gastrointestinal tract cancers (Taylor et al., 2018), and is linked to several disorders, including congenital malformations, developmental delays, and intellectual disability (Hogendorf et al., 2021). However, the impact of 18q loss on the functional properties of hPSCs is unknown. Therefore, the aim of this work was to examine the functional effects of 18q deletions during hPSC differentiation into the three embryonic germ layers and to determine the molecular mechanisms involved.

RESULTS

The minimal common region of 18q loss spans 14 genes expressed in undifferentiated hESCs and includes *SALL3*

We initially identified 18q losses in the hESC lines Vrije Universiteit Brussel (VUB)04 and VUB26, in the form of a derivative chromosome 18. VUB04 presented a deletion at 18q21.2qter and a duplication at 5q14.2qter, and VUB26 showed the minimal 18q loss region (18q23qter) and a duplication at 7q33qter (Figure S1; Table S1) (Spits et al., 2008). These two hESC lines were not used in the present work because they further genetically drifted and acquired gain of 1q and 20q11.21, but their analysis helped to narrow down the common 18q deletion region. In the present study, we used two other hESC lines bearing derivative chromosomes 18 involving a loss of 18q losses (hESC^{del18q}), VUB14^{del18q}, and VUB13^{del18q}, as well as three chromosomally balanced lines (hESC^{WT} [wild type]), VUB14^{WT}, and VUB04^{WT} and VUB03^{WT}, which served as controls for VUB13^{del18q} since VUB13^{WT} was lost (details on the karyotypes of the lines and their characterization are shown in Figure S2; Table S1). We used shallow genome sequencing to confirm the karyotypes before starting the experiments, and all of the lines were routinely inspected, with qPCR assays targeting recurrent chromosomal abnormalities (1q, 12p, 20q11.21, and 17q) to confirm their genomic stability for the duration of the different experiments.

The 18q losses exhibited a common loss region from base pairs 75,773,285 to 80,373,285, spanning 37 loci. Bulk RNA sequencing (RNA-seq) of undifferentiated hESCs indicated that 14 genes within this region are expressed in undifferentiated hESCs, with counts per million greater than one in at least two samples (Figure S1A). Of these coding genes, *ADNP2*, *SALL3*, and *TXNL4A* had the highest expression and showed decreased transcript levels in mutant cells. *ADNP2* and *TXNL4A* have no known function in hPSC. *ADNP2* is predicted to be a transcription factor, and its silencing increases oxidative stress-mediated cell death (Kushnir et al., 2008). *TXNL4A* is a component of the U5 small ribonucleoprotein particle, which is involved in pre-mRNA splicing and is associated with Burn-McKeown syndrome (Wood et al., 2022). *SALL3* was more promising as a candidate driver gene because it has previously been reported to regulate the differentiation propensity of hiPSC lines (Kuroda et al., 2019). Kuroda et al. showed that hiPSC lines expressing high levels of *SALL3* differentiated preferentially into ectoderm, whereas hiPSC lines expressing lower levels of *SALL3* tended to differentiate into mesoderm and endoderm (Kuroda et al., 2019). It has also been shown that *SALL3* interacts with the Mediator complex in neural stem cells (Quevedo et al., 2019) and is related to the development of the nervous system (Ott et al., 1996). Considering these previous findings, we hypothesized that the decreased expression of *SALL3* because of a loss of one copy of the gene could alter the differentiation capacity of hESCs^{del18q}.

hESCs with 18q loss show impaired neuroectoderm differentiation

As a first step, we investigated the effect of 18q deletion on hESC ectoderm lineage commitment. $hESC^{WT}$ and $hESC^{del18q}$ were subjected to neuroectoderm differentiation for 8 days using LDN193189 (LDN), SB431542 (SB) and retinoic acid (RA) (Douvaras and Fossati, 2015) (Figure S1B). We measured the mRNA levels of different neuroectoderm markers to evaluate neuroectoderm differentiation efficiency and the expression of the undifferentiated state markers *NANOG* and *POUF51* (Figures 1A and S3A). $VUB13^{del18q}$ and $VUB14^{del18q}$ had significantly lower mRNA levels of *PAX6*, *NES*, and *SOX1*, compared to the levels in $hESC^{WT}$ (for all genes and both lines, $p \leq 0.0001$, unpaired t test), indicating a decreased neuroectodermal differentiation efficiency in $hESC^{del18q}$. *POU5F1* and *NANOG* mRNA expression levels were almost undetectable for all differentiated cells (Figure S3A). We also evaluated the differentiation of $hESC^{WT}$ and $hESC^{del18q}$ cells by immunostaining (Figures 1B and 1C). We observed a lower percentage of $PAX6^{+}$ cells in differentiated $hESC^{del18q}$ than in differentiated $hESC^{WT}$ cells (70% in $hESC^{WT}$ vs. 50% in $VUB13^{del18q}$ and 41% in $VUB14^{del18q}$; Figure 1C), which was consistent with the decrease in the levels of *PAX6* mRNA. Taken together, these results show that $hESC^{del18q}$ differentiation into neuroectoderm is impaired, and rather than to remain undifferentiated state, they misspecify.

$hESC^{del18q}$ readily differentiates into mesendoderm derivatives but shows abnormal cardiomyocyte progenitor differentiation

We further investigated the impact of 18q deletion on the mesendoderm differentiation capacity of hESCs by differentiating $hESC^{WT}$ and $hESC^{del18q}$ into, in this case, cardiac progenitors and hepatoblasts. Thus, we induced the differentiation of $hESC^{WT}$ and $hESC^{del18q}$ into mesoderm using a 5-day cardiac progenitor induction protocol described previously (Lin and Zou, 2020) (Figure S1B). We evaluated the mRNA levels of the cardiac progenitor markers *GATA4*, *ISL1*, *NKX2-5*, and *PDGFRA* (Figure 2A). $hESC^{del18q}$ showed lower levels of *GATA4* mRNA ($p = 0.02$ for $VUB13^{del18q}$ and $p = 0.01$ $VUB14^{del18q}$, unpaired t test; Figure 2A). $hESC^{del18q}$ lines expressed *ISL1* at 15-fold higher levels, on average, than $hESC^{WT}$ lines ($p < 0.0001$ for $VUB13^{del18q}$ and $p = 0.0005$ $VUB14^{del18q}$, unpaired t test; Figure 2A). We found no significant or consistent difference in the expression levels of *NKX2-5* or *PDGFRA* between the control and mutant cardiac progenitor groups ($p = 0.01$ for *NKX2-5* $VUB14^{del18q}$, others not significant, unpaired t test; Figure 2A). We also evaluated the proportion of differentiated cardiac progenitor and undifferentiated cells by immunostaining for GATA4 and POU5F1. The percentage of $GATA4^{+}$ cells was overall lower in $hESC^{WT}$ than in $hESC^{del18q}$, whereas the cells were $POU5F1^{-}$ (Figures 2B and 2C). Taken together, and bearing in mind the temporal expression of these markers during cardiac differentiation (Doyle et al., 2015; Zhang et al., 2019), the results suggest that $hESC^{del18q}$ may experience differentiation delays or arrest, reaching an $ISL1^{high}$ $GATA4^{low}$ stage at day 5 but remaining less mature than their $hESC^{WT}$ counterparts.

Next, we differentiated $hESC^{WT}$ and $hESC^{del18q}$ into hepatoblasts by applying the modified differentiation protocol for 8 days, as described previously (Boon et al., 2020) (Figure S1B). We measured the expression levels of the hepatoblast markers *HNF4A*, *AFP*, *ALB*, and *FOXA2* (Figure 3A). We found no consistent difference in the mRNA expression levels of *HNF4A*, *ALB*, *FOXA2*, and *AFP* between $hESC^{WT}$ and $hESC^{del18q}$ (not significant for $VUB13^{del18q}$, $p < 0.0001$, $p = 0.0002$, $p = 0.03$, and $p = 0.0016$, respectively, for $VUB14^{del18q}$, unpaired t test; Figure 3A). We further evaluated hepatoblast differentiation by immunostaining for HNF4A and found that the percentages of $HNF4A^{+}$ cells in $hESC^{del18q}$ cells were in line with the mRNA expression. $VUB13^{del18q}$ showed results similar to those in WT cells (58% in $hESC^{WT}$ vs. 53% in $VUB13^{del18q}$), whereas $VUB14^{del18q}$ had 23% positive cells (Figures 3B and 3C).

For both mesodermal and endodermal lineage commitment, all $hESC^{WT}$ and $hESC^{del18q}$ lines displayed low mRNA and protein levels of undifferentiated state markers (Figures S3B and S3C), indicating a loss of pluripotency for all cell lines during differentiation. Overall, our results indicate that $hESC^{WT}$ and $hESC^{del18q}$ differentiate into definitive mesoderm and endoderm and that there may be a delay or impairment in the progression of $hESC^{del18q}$ toward the cardiac progenitor stage. The differences in gene expression observed during hepatoblast differentiation are likely due to between-line variation in differentiation propensity rather than to the 18q deletion itself.

Downregulation of *SALL3* impairs neuroectoderm differentiation but does not affect differentiation into mesoderm and endoderm

We next examined *SALL3* mRNA expression levels in undifferentiated cells and found that *SALL3* expression was significantly lower in $hESC^{del18q}$ than in $hESC^{WT}$ (Figures S1A and S3D), supporting the notion that *SALL3* could be a key gene in the altered differentiation capacity of $hESCs^{del18q}$. We first generated *SALL3* knockdown (KD) lines from three $hESC^{WT}$ lines ($VUB02^{WT}$, $VUB03^{WT}$, and $VUB04^{WT}$) by transducing a lentiviral vector containing short hairpin RNA (shRNA) targeting the *SALL3* transcript ($hESC^{WT\_SALL3KD}$) or a nontargeting shRNA as a control ($hESC^{WT-NT}$) (Figure S3E). Next, we generated $hESC^{del18q}$ with stable overexpression (OE) of *SALL3* ($hESC^{del18q\_SALL3OE}$) by transducing $VUB13^{del18q}$ and $VUB14^{del18q}$ with the *SALL3* lentiviral vector, and we verified

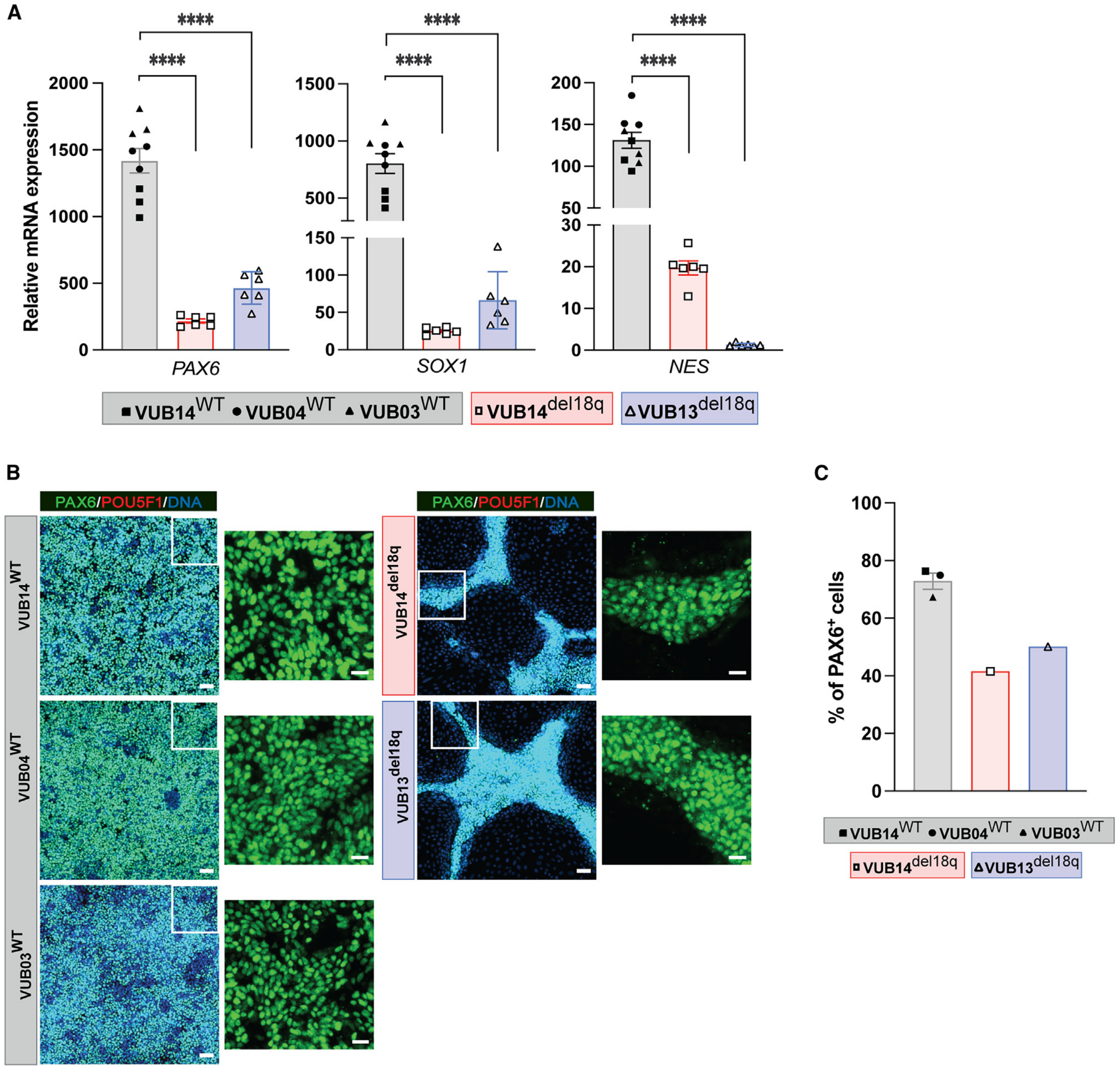

Figure 1. hESCs with 18q loss show impaired neuroectoderm differentiation

(A) Relative mRNA expression for neuroectoderm markers. Data are shown as means ± SEMs. Each data point refers to an independent differentiation experiment, and *, **, ***, and **** represent statistical significance between samples at 5%, 1%, 0.1%, and 0.01%, respectively (unpaired t test).

(B) Immunostaining in mutant and control lines. Scale bars (original images), 50 μm, scale bars (magnification images), 20 μm.

(C) Percentages of PAX6^{+} cells in the immunostainings shown in (B).

the OE by measuring *SALL3* mRNA levels, which were significantly increased in VUB13^{del18q_SALL3OE} (3-fold) and VUB14^{del18q_SALL3OE} (5-fold) compared to controls (Figure S3F).

To investigate the role of *SALL3* in regulating hESC differentiation propensity, we first induced neuroectodermal differentiation in hESC^{WT_SALL3KD}, hESC^{del18q_SALL3OE}, and the corresponding control cells. All three hESC^{WT_SALL3KD} lines had lower levels of all neuroectodermal (NE) markers than nontarget controls (Figures 4A, S4A, and S4B). PAX6 protein levels were also reduced in hESC^{WT_SALL3KD} (Figures 4B and 4C), with only 20% of the cells expressing PAX6, compared to 80% of PAX6^{+} cells in the control group (Figures 4B and 4C). Our results show that *SALL3* suppression in hESC^{WT}

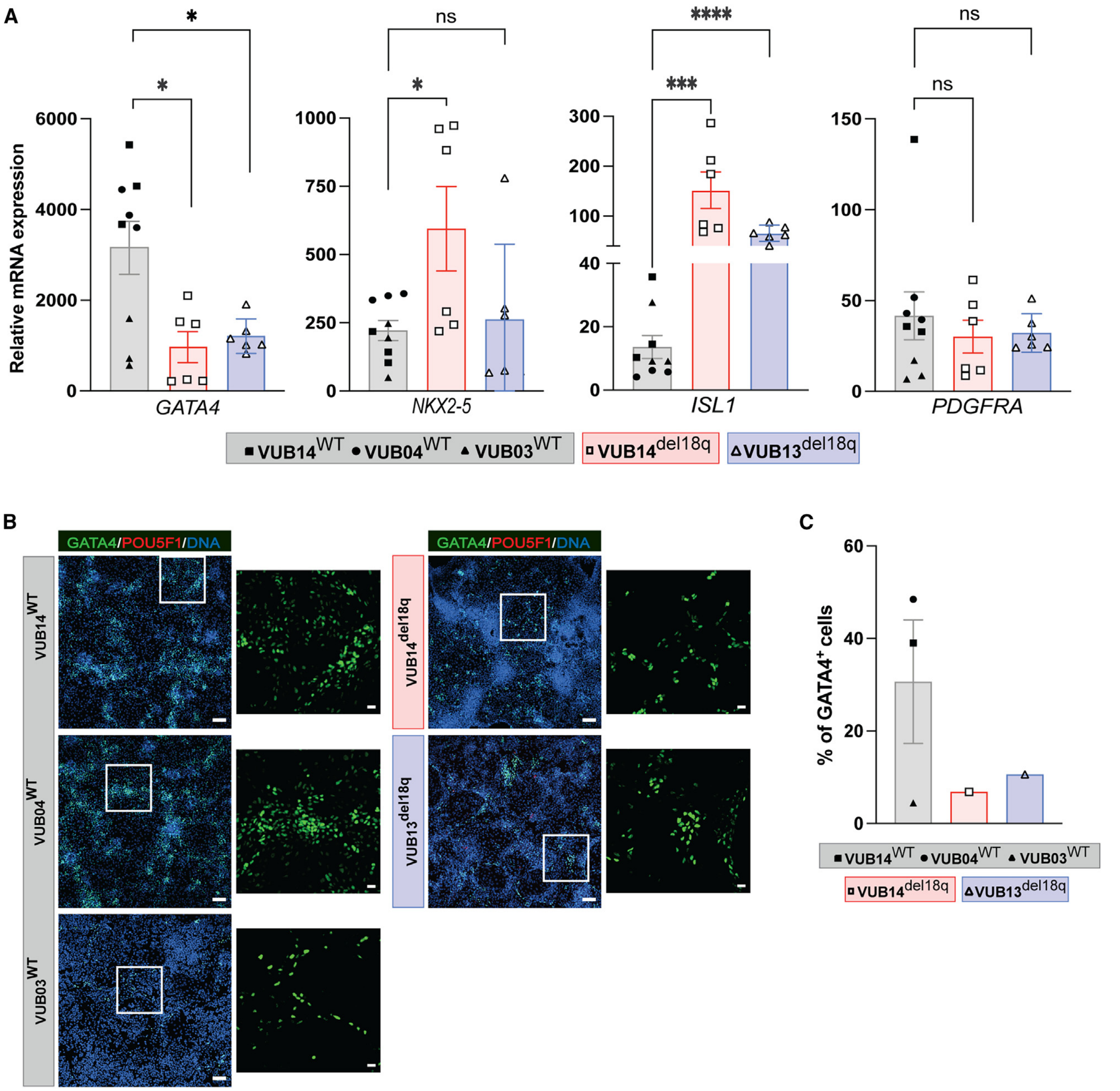

Figure 2. hESC^{del18q} cells show abnormal cardiac progenitor differentiation

(A) Relative mRNA expression for cardiac progenitor markers. Data are shown as means ± SEMs. Each data point refers to an independent differentiation experiment, and *, **, ***, and **** represent statistical significance between samples at 5%, 1%, 0.1%, and 0.01% respectively (unpaired t test). ns, not significant.

(B) Immunostaining for GATA4 and POU5F1 in mutant and control lines. Scale bars (original images), 100 μm, and scale bars (magnification images), 20 μm.

(C) Percentage of GATA4^{+} cells in the immunostainings shown in (B).

recapitulates the impaired NE differentiation seen in hESC^{del18q} lines. In contrast, hESC^{del18q_SALL3OE} cells efficiently differentiated into neuroectoderm cells, accompanied by a significant increase in the mRNA levels of *PAX6*, *SOX1*, and *NES* (Figures 4A, S4A, and S4B); moreover, hESC^{del18q_SALL3OE} cultures included more PAX6^{+} cells than hESC^{del18q} cultures (60% vs. 20%, respectively) (Figures 4B and 4C). These results indicate that exongenous *SALL3* expression can rescue the impairment of ectoderm differentiation caused by 18q loss.

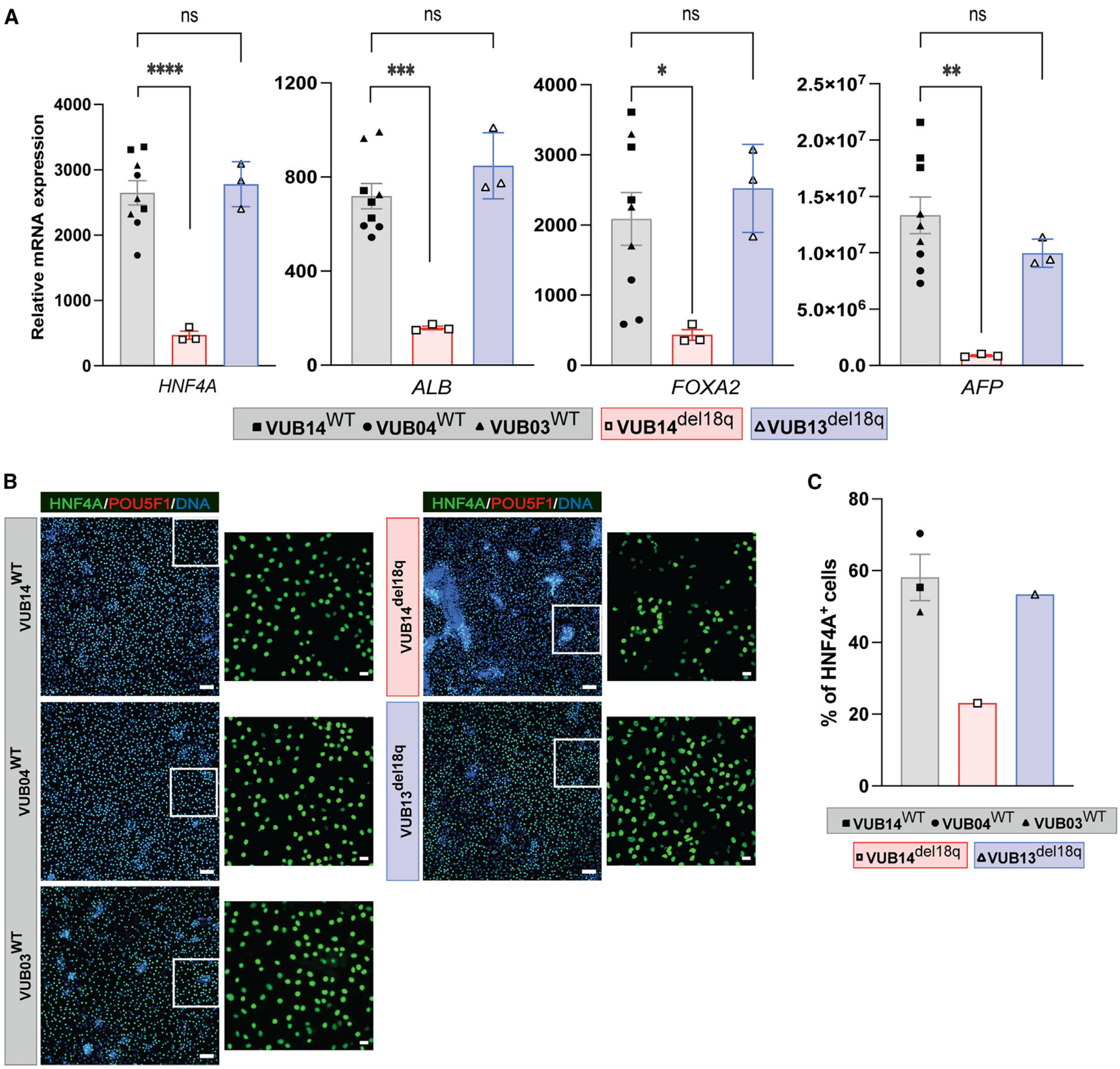

Figure 3. hESC^{del18q} and their genetically balanced counterparts differentiate equally well into hepatoblasts

(A) Relative mRNA expression of hepatoblast markers. Data are shown as means ± SEMs. Each data point refers to an independent differentiation experiment, and *, **, ***, and **** represent statistical significance between samples at 5%, 1%, 0.1%, and 0.01%, respectively (unpaired t test).

(B) Immunostaining for HNF4A and POU5F1 in mutant and control lines. Scale bars (original images), 100 μm, and scale bars (magnification images), 20 μm.

(C) Percentage of HNF4A$^+$ cells in the immunostainings shown in (B).

Next, we induced the differentiation of the different hESC lines into cardiac progenitors. The three hESC^{WT_SALL3KD} lines differentiated inconsistently toward mesoderm fates. VUB04^{WT_SALL3KD} showed lower levels of *GATA4* (Figure 5A; p = 0.0003, unpaired t test), whereas compared to the controls, VUB03^{WT_SALL3KD} and VUB02^{WT_SALL3KD} showed no differences in *GATA4* expression (Figure 5A). In addition, the percentage of GATA4$^+$ cells detected by immunostaining in hESC^{WT_SALL3KD} followed the same pattern, consistent with the mRNA levels of each line (Figures 5B and 5C). The hESC^{del18q_SALL3OE} cells exhibited variable marker profiles, with increases in GATA4 expression at both the mRNA

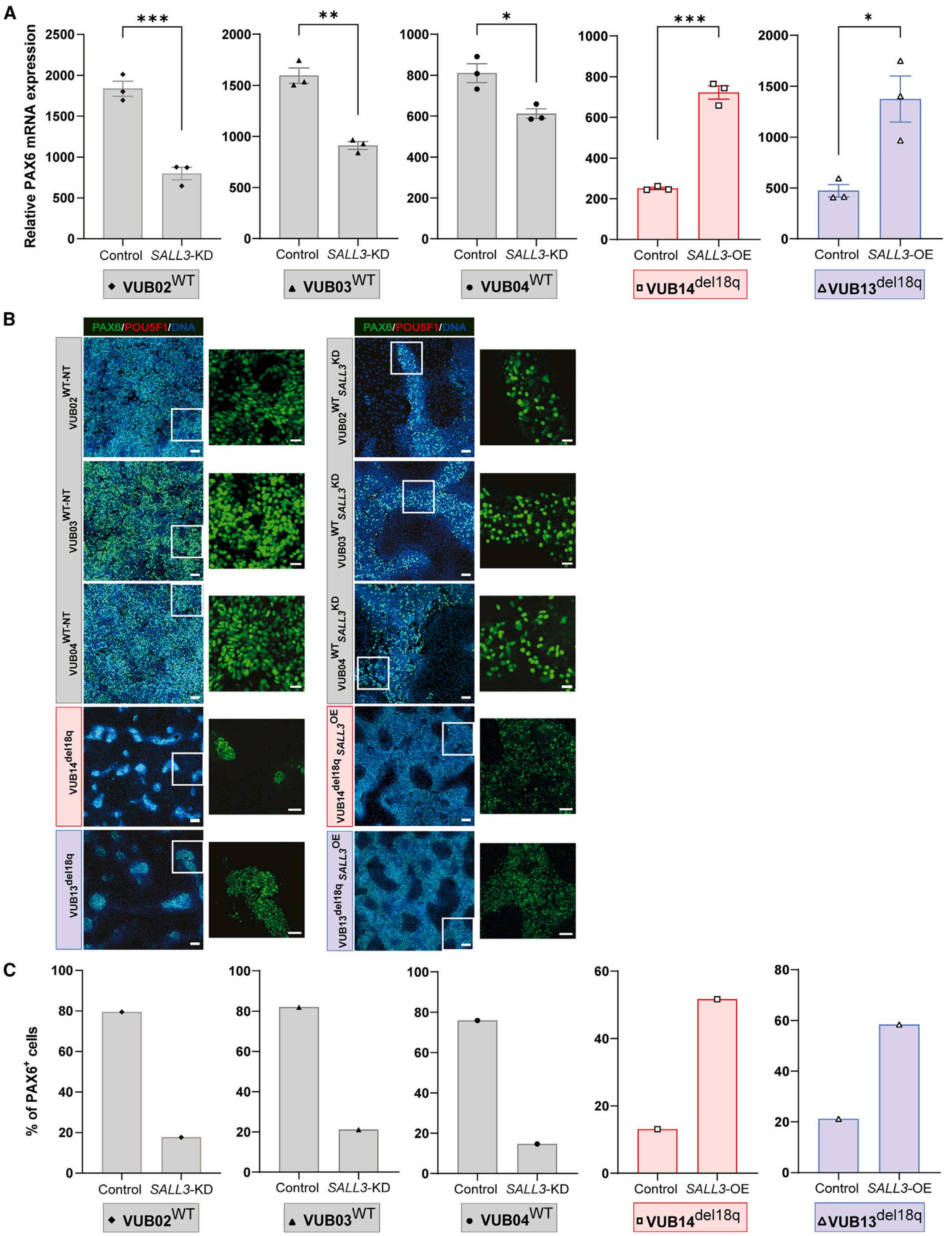

(legend on next page)

(Figure 5A; p = 0.004, unpaired t test) and protein levels for VUB13^{del18q_SALL3OE}, but no difference in *GATA4* mRNA levels (Figure 5A; p = 0.3622, unpaired t test) and a slight decrease in GATA4 protein levels in VUB14^{del18q_SALL3OE} (Figures 5B and 5C). Similarly, the mRNA expression levels of other markers, *NKX2-5*, *ISL1*, and *PDGFRA*, showed no consistent trend in the hESC^{WT_SALL3KD} groups and exhibited no consistent differences in hESCs^{del18q} and hESC^{del18qSALL3OE} (Figures S5A–S5C).

When hESCs were differentiated into hepatoblast, *SALL3* downregulation in hESC^{WT} resulted in increased *HNF4A*, *ALB*, and *FOXA2* mRNA expression (Figures 6A, S6B, and S6C). The percentage of HNF4A^{+} cells was higher in VUB03^{WT_SALL3KD} cells, but not in VUB04^{WT_SALL3KD} and VUB02^{WT_SALL3KD} cells compared to controls (Figures 6B and 6C). The mRNA expression of another marker, *AFP*, also did not show consistent changes (Figure S6A). Upon the OE of *SALL3*, the differentiation profiles into hepatoblast did not show the expected mirroring effect. The mRNA expression of all of the hepatoblast markers *HNF4A*, *ALB*, *AFP*, and *FOXA2* increased in VUB14^{del18q_SALL3OE} cells, consistent with the changes observed in the HNF4A protein level, but this same effect was not observed in VUB13^{del18q_SALL3OE} cells (Figures 6 and S6).

Overall, these results suggest that the effect of *SALL3* on mesoderm and endoderm differentiation may be line specific and is not the reason for the delayed progression seen during cardiac differentiation in hESCs^{18q}.

Downregulation of *SALL3* and loss of 18q result in the deregulation of genes in pathways associated with pluripotency and differentiation

To gain deeper insight into the effect of *SALL3* downregulation on the global transcriptomic profile of cells with 18q loss, we carried out bulk RNA-seq of hESC^{WT-NT} (N = 6), hESC^{WT_SALL3KD} (N = 6), hESC^{WT} (N = 6), VUB13^{del18q} (N = 4), and VUB13^{del18q_SALL3OE} (N = 5) cells. To estimate the similarity among the samples, we generated a distance clustering heatmap with global row scaling (Figure 7A) and performed principal-component analysis (PCA) (Figure 7B). The heatmap shows that although VUB13^{del18q} and hESC^{WT_SALL3KD} cluster together, they cluster apart from the WT cell lines and from VUB13^{del18q_SALL3OE}. The samples in this last group clustered more closely to the WT cell lines than to their unmodified VUB13^{del18q} (Figure 7A). This pattern was also reflected in the first dimension of the PCA (Figure 7B), in which hESC^{WT}, hESC^{WT-NT}, and VUB13^{del18q_SALL3OE} clustered more closely with one another than with VUB13^{del18q} and hESC^{WT_SALL3KD}. These results suggest that the downregulation of *SALL3* in WT cells is sufficient to alter the transcriptome such that its profile is closer to that of an hESC line with a loss of 18q, and *SALL3* OE in hESC^{del18q} can restore the transcriptome to a near-WT state. Taken together, these findings support our hypothesis that the differences between hESC^{WT} and hESC^{del18q} are driven mostly by the downregulation of *SALL3* due to the loss of one copy of this gene. For this reason, we pooled the hESC^{WT-NT} with the untreated hESC^{WT} for further analysis.

Next, we carried out differential gene expression analysis to gain further insight into which genes and pathways form the basis of the differences between hESC^{WT} and hESC^{del18q} and which of these are driven by *SALL3*. Figures 7C–7E show volcano plots of the differentially expressed genes in the different groups. We considered this differential expression to be significant at a $|\log_2\text{fold change}| > 1.0$ and false discovery rate (FDR) < 0.05. VUB13^{del18q} shows 891 and 448 genes that are significantly upregulated and downregulated in hESC^{del18q}, respectively, compared to the WT cells (Figure 7C). Downregulation of *SALL3* in WT cells led to the differential expression of 745 genes, 399 of which were upregulated and 346 of which were downregulated (Figure 7D). OE of *SALL3* in VUB13^{del18q} resulted in the upregulation of 121 genes and the downregulation of 605 genes (Figure 7E).

To elucidate which of the transcriptional differences between hESC^{WT} and hESC^{del18q} are mediated by *SALL3*, we investigated the overlaps in up- and downregulated genes across conditions. First, we found that 231 and 93 genes were commonly up- and downregulated, respectively, between VUB13^{del18q} and hESC^{WT_SALL3KD}, representing 26% of the differentially expressed genes in hESCs with an 18q deletion (Figures 7F and 7G). Second, we compared these subsets of genes to the genes with altered gene expression in hESC^{del18q} upon OE of *SALL3*. We compared the genes that were upregulated by *SALL3* KD and by loss of 18q to those downregulated by OE of *SALL3* in hESC^{18q}, and vice versa (Figures 7F and 7G). Of the 231 upregulated genes shared by the VUB13^{del18q} and hESC^{WT_SALL3KD} groups, 173 genes showed increased expression in VUB13^{del18q_SALL3OE}

Figure 4. Downregulation of *SALL3* drives the impaired neuroectoderm differentiation of hESCs with 18q loss

(A) Relative mRNA expression for *PAX6*. Data are shown as means ± SEMs. Each data point refers to an independent differentiation experiment, and *, **, ***, and **** represent statistical significance between samples at 5%, 1%, 0.1%, and 0.01%, respectively (unpaired t test).

(B) Immunostaining for PAX6 and POU5F1 in mutant and control lines. Scale bars (KD groups), 50 μm, and magnification 20 μm; scale bars (OE groups), 100 μm, and magnification, 50 μm.

(C) Percentage of PAX6^{+} cells in the immunostainings shown in (B).

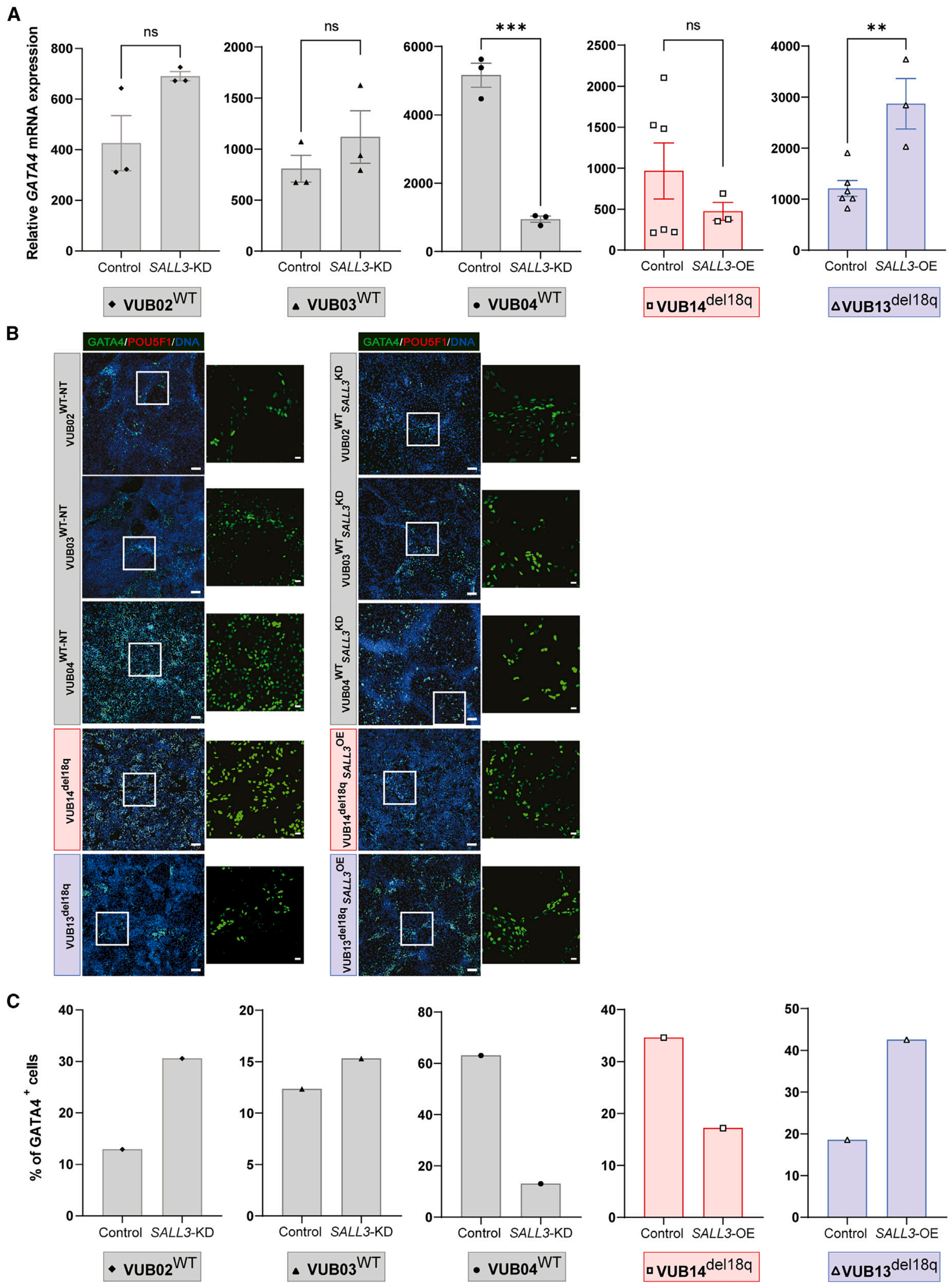

(legend on next page)

(Figure 7F). In addition, 18 of the 93 downregulated genes shared by VUB13^{del18q} and hESC^{WT_SALL3KD} displayed increased expression in VUB13^{del18q}*SALL3*^{OE} (Figure 7G). This further refined the gene set to a core of 191 genes that are the most strongly regulated by *SALL3* in hESCs, both by the loss of a copy of the gene itself and by the modulation of its expression (Table S2). The other differences in gene expression are likely associated with the loss of other genes in 18q or with the OE of genes in the duplicated regions of chromosomes 5 and 7, which are part of the derivative chromosome 18 in hESC^{del18q}.

Finally, to identify potential molecular targets and elucidate the underlying functional mechanisms that contribute to the observed impairment of differentiation capacity in hESC^{del18q}, we analyzed the differential gene expression of the three groups using gene set enrichment analysis and the MSigDB database. Specifically, we focused on the Kyoto Encyclopedia of Genes and Genomes and WikiPathways databases within the C2 library and the pathways of the H library. We filtered the significant pathways based on a normalized enrichment score $|NES| > 1$, a $p < 0.05$, and the proportion of leading-edge genes accounting for >30% of the entire gene set involved in the pathway.

Figures 7H and 7I show Venn diagrams of the overlap between significantly enriched pathways in the C2 and H libraries, respectively, for each of the three groups. The full list can be found in Table S3. In total, we found 13 pathways that overlapped among the 3 groups, all 13 of which were positively enriched in both VUB13^{del18q} and hESC^{WT_SALL3KD} and negatively enriched in VUB13^{del18q_SALL3OE} (Figure 7J shows the cell-type-relevant pathways; the whole set can be found in Table S4). Because of the critical role that these pathways play in pluripotency maintenance and differentiation, we analyzed the expression of pluripotency-associated genes, and we found that *NANOG*, *POU5F1*, *LIN28*, *SOX2*, *PODXL*, *SUSD2*, *MYC*, *FOXD3*, and *DPPA3* are overexpressed in VUB13^{del18q}, with all but *DPPA3* and *POU5F1* also being overexpressed upon *SALL3*^{KD} and downregulated by transgenic *SALL3* OE in VUB13^{del18q} (Figure S7A).

DISCUSSION

In this study, we examined the repercussions of the loss of chromosome 18q on the differentiation capacity of hESCs. For this, we used an early-stage differentiation approach to generate lineage-specific cell types representing the three germ layers: neuroectoderm, hepatoblast, and cardiac progenitors. Our *in vitro* lineage commitment studies indicated that the deletion of 18q in hESCs impaired neuroectodermal differentiation and delayed cardiac progenitor differentiation, whereas no consistent differences were observed in the commitment toward hepatoblasts. It is important to bear in mind that we used a set of three or four markers to establish the cell identity after differentiation, precluding an in-depth analysis of the impact of the mutation on the further maturation and functionality of the obtained cells.

To study the mechanistic basis for these changes in differentiation, we looked at the genes located in the minimal region of loss. We found that decreased *SALL3* expression due to the loss of one copy of the gene was sufficient to result in the observed decreased neuroectoderm differentiation, but not to modulate cardiac and hepatoblast differentiation. In this sense, our results are only partially aligned with those obtained by Kuroda et al. (2019). These authors found that downregulating *SALL3* resulted not only in decreased neuroectoderm differentiation, similar to our results, but also in increased cardiac progenitor differentiation. Although we can only speculate about the reasons for these differences, it is likely that the genetic background of the cell lines plays an important role (Kilpinen et al., 2017). Also, given that *SALL3* has been reported to be a modulator of DNMT3A and DNMT3B activity (Kuroda et al., 2019; Shikauchi et al., 2009), the preexisting epigenetic marks in each of the lines, particularly histone modifications, may influence the recruitment of *de novo* methyl transferases to methylate the DNA (Baubec et al., 2015; Weinberg et al., 2019). Furthermore, it is possible that other genes located in 18q, or in the gain regions of chromosomes 5 and 7, cause cell-line-specific effects.

In line with this reasoning, the gene expression analysis revealed that only part of the divergence between hESCs with 18q loss and their chromosomally normal isogenic counterparts was related to the differential expression of *SALL3*. Interestingly, the core set of deregulated genes was associated with pathways involved in the maintenance of and exit from the undifferentiated pluripotent state, and key regulators of pluripotency were both upregulated in hESCs^{18q} and regulated by *SALL3* expression. For instance, transforming growth factor β (TGF-β) signaling is key to the

Figure 5. Changes in *SALL3* expression do not regulate cardiac progenitor differentiation
(A) Relative mRNA expression for *GATA4*. Data are shown as means ± SEMs. Each data point refers to an independent differentiation experiment, and *, **, ***, and **** represent statistical significance between samples at 5%, 1%, 0.1%, and 0.01%, respectively (unpaired t test).
(B) Immunostaining for GATA4 and POU5F1 in mutant and control lines. Scale bars (original images), 100 μm, and scale bars (magnification images), 20 μm. (C) Percentage of GATA4^{+} cells in the immunostainings shown in (B).

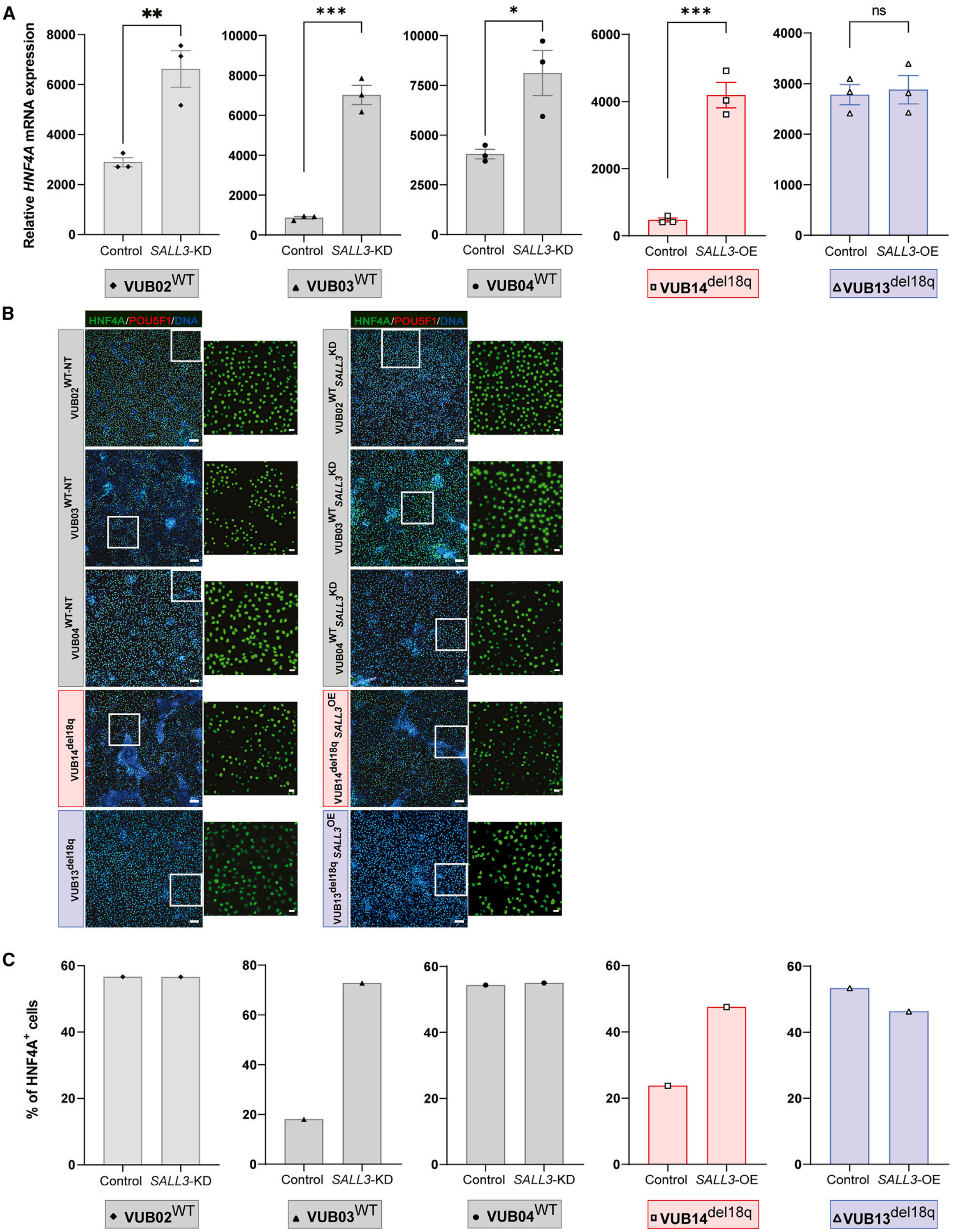

(legend on next page)

maintenance of the primed pluripotent state (Weinberger et al., 2016). TGF-β and bone morphogenetic protein 4 (BMP4) signaling are also core genes in regulating the balance between neuroectoderm differentiation and mesendoderm in both humans and mice (Park, 2011). Notch activation mediates TGF-β signaling during hESC and mesenchymal stem cell differentiation into smooth muscle cells (Kurpinski et al., 2010), and its inhibition supports naive state consolidation in rodent models (Weinberger et al., 2016). The cytokine tumor necrosis factor α has been shown to negatively regulate the differentiation of various cell types, including cardiomyocytes (Hamid et al., 2016) and embryoid bodies (Wuu et al., 1998). In addition, nuclear factor-κB inhibition has been found to mediate naive pluripotency in mice (Dutta et al., 2011). Overall, these results suggest that downregulation of *SALL3* due to the loss of 18q alters undifferentiated-state maintenance in hESCs by affecting pluripotency-associated pathways, and these changes have profound effects on the differentiation capacity of the cells.

With hPSCs steadily moving into clinical trials (Kobold et al., 2020) and being broadly used as a cell source for *in vitro* modeling of, for instance, developmental processes and diseases, determining the impact of recurrent genetic abnormalities is critical (Andrews et al., 2022; Keller et al., 2018). Work from our group and others is beginning to generate a detailed picture showing how these genetic abnormalities affect differentiation in a cell lineage-specific manner. For instance, 20q11.21 gain impairs neuroectoderm commitment without affecting mesendoderm induction (Jo et al., 2020; Markouli et al., 2019), and recently, it has been shown that cells with an isochromosome 20q are not able to survive retinal pigmented epithelial cell differentiation and display overall disruptions in the ability to correctly differentiate (Vitillo et al., 2023). In this work, we show that 18q loss specifically impairs neuroectoderm commitment and appears to delay cardiac differentiation. Taken together, these results highlight the importance of the genetic screening of hPSC cultures to ensure that these abnormalities do not pass unnoticed. In a research setting, chromosomal abnormalities could lead to confounding effects that decrease the reliability and reproducibility of the work, and in a clinical setting, they could lead at best to decreased therapeutic efficacy and at worst to tumorigenesis (Andrews et al., 2022; Keller et al., 2018; Yamanaka, 2020).

In conclusion, in this study, we have characterized the differentiation capacity of hESCs with 18q loss, one of the recurrent, albeit less common, genetic abnormalities found in hPSC cultures. We found that these cells are characterized by abnormal differentiation into cardiac progenitors and an impaired capacity for neuroectoderm commitment, the latter driven by the loss of one copy of *SALL3*. This gene is an inhibitor of *DNMT3B*, and its downregulation results in changes in the expression of genes involved in the maintenance of pluripotency and in hESC differentiation. Further research will be needed to assess whether other cell-type-specific effects of this abnormality exist and may be revealed by longer differentiation protocols, beyond the progenitor stage, as well as the potential consequences of 18q loss for oncogenic potential.

EXPERIMENTAL PROCEDURES

Resource availability

Lead contact

Further information and requests for resources should be directed to the corresponding author, Claudia Spits (claudia.spits@vub.be).

Materials availability

All VUB stem cell lines in this study, including the genetically abnormal sublines and genetically modified lines, are available upon request and after signing a materials transfer agreement.

Data and code availability

Raw sequencing data of human samples are considered personal data by the General Data Protection Regulation of the European Union (Regulation (EU) 2016/679), because SNPs can be extracted from the reads and cannot be shared publicly. The data can be obtained from the corresponding author upon reasonable request and after signing a data use agreement. The RNA-seq counts per million tables are provided in the Data S1, which allow for downstream gene expression analysis. The data supporting all of the figures in this paper can be found at the Open Science Framework repository: https://osf.io/hpaxc/.

Ethics statement

For all parts of this study, the design and conduct complied with all of the relevant regulations regarding the use of human materials, and all of them were approved by the local ethical committee of the University Hospital UZ Brussel and the Vrije Universiteit Brussel (file no. B.U.N. 1432020000284). All of the patients donating embryos to derive hESC lines gave written consent.

Figure 6. Changes in *SALL3* expression do not consistently affect hepatoblast differentiation

(A) Relative mRNA expression of *HNF4A*. Data are shown as means ± SEMs. Each data point refers to an independent differentiation experiment, and *, **, ***, and **** represent statistical significance between samples at 5%, 1%, 0.1%, and 0.01%, respectively (unpaired t test).

(B) Immunostaining for HNF4A and POU5F1 in mutant and control lines. Scale bars (original images), 100 μm, and scale bars (magnification images), 20 μm.

(C) Percentage of $HNF4A^{+}$ cells.

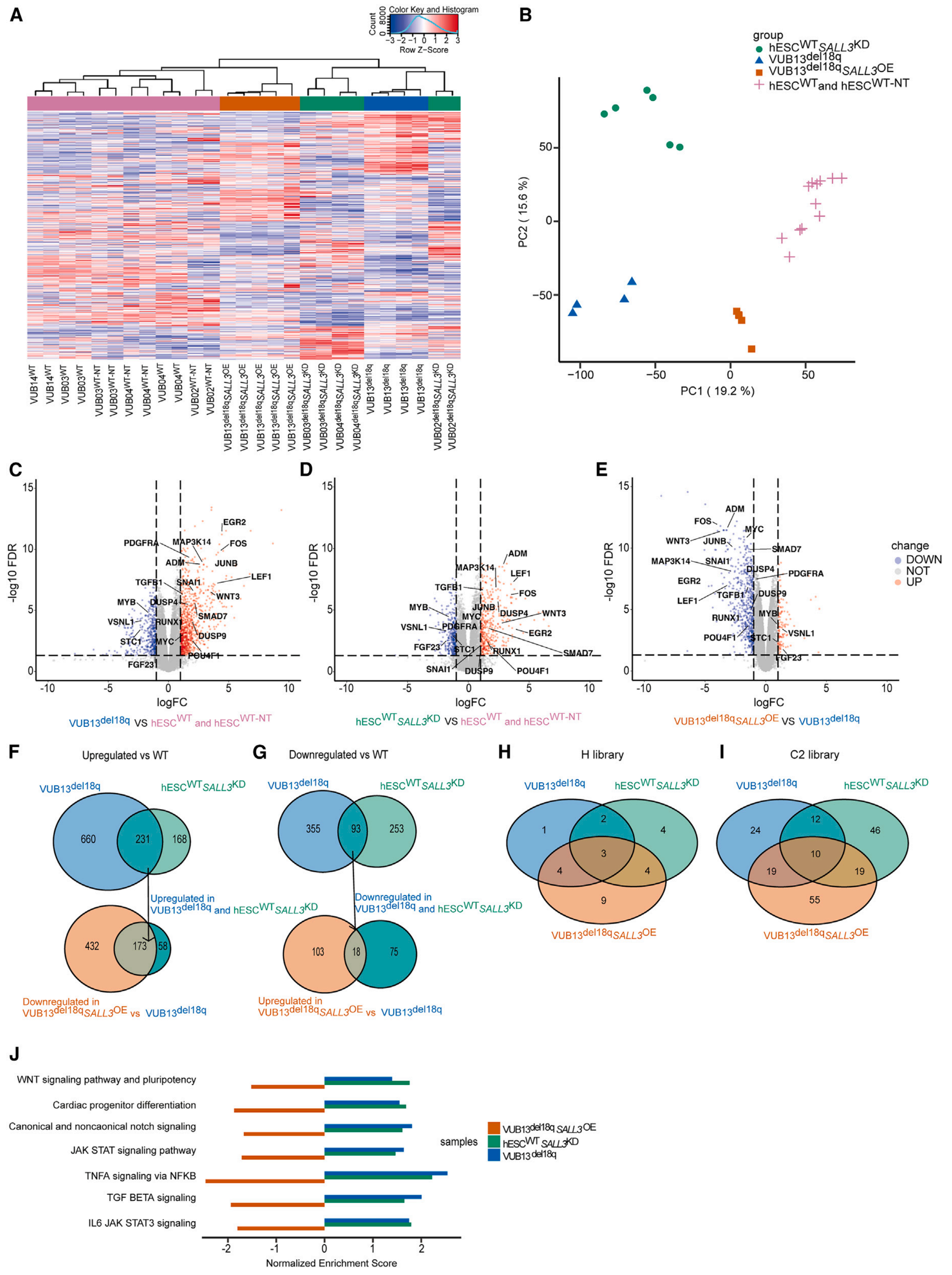

(legend on next page)

hESC maintenance and passaging

All of the hESC lines in this study were derived in-house in the past. The details on the derivation and results of the characterization, including tests for pluripotency, were reported previously (Mateizel et al., 2006; 2010) and can be also found at the Open Science Framework repository: https://osf.io/esmz8/. The lines are registered in the EU hPSC registry (https://hpscreg.eu/) and available upon request. All of the experiments in this study have been carried out on cells that belong to the same working cell bank, each of which tested for genetic content by shallow whole-genome sequencing and for mycoplasma. No cells were used beyond five passages after being drawn from the working bank.

The hESCs were maintained in NutriStem hESC XF medium (NS medium; Biological Industries) with 100 U/mL penicillin/streptomycin (Thermo Fisher Scientific) in a 37°C incubator with 5% CO_2 on Biolaminin 521-coated dishes (Biolamina). The culture medium was changed daily. The cells were passaged as single cells using TrypLE Express (Thermo Fisher Scientific) and split when reaching 70%–90% confluence. The medium was supplemented with 10 μM rho kinase inhibitor Y-27632 (Tocris) for the first 24 h after passaging.

Copy-number variant (CNV) analysis

The genetic content of the hESCs was assessed through shallow whole-genome sequencing by the BRIGHTcore of UZ Brussels, Belgium, as previously described (Bayindir et al., 2015). We also conducted CNV analysis using quantitative real-time PCR at regular intervals, particularly before and after performing lentiviral transduction and starting differentiation. DNA was extracted with a DNeasy Blood and Tissue Kit (Qiagen) according to the manufacturers' protocol. qPCR was performed with the copy-number assays *RNaseP* (Thermo Fisher Scientific) as a reference and *KIF14*, *NANOG*, *NMT1*, and *ID1* (Thermo Scientific) covering the 1q, 12p, 17q, and 20q regions, respectively. The reaction systems were prepared by mixing TaqMan 2× Mastermix Plus–Low ROX (Eurogentec) and the TaqMan assays together with the DNA samples. qPCR was performed on a ViiA7 thermocycler (Thermo Fisher Scientific), and Applied Biosystems Copy Caller version 2.1 was used to analyze the CNVs.

Total RNA isolation, cDNA synthesis, and quantitative real-time PCR for gene expression analysis

Total RNA was isolated using RNeasy Mini and Micro kits (Qiagen) following the manufacturer's guidelines, including on-column DNase I treatment. mRNA was reverse transcribed into biotinylated cDNA using the First-Strand cDNA Synthesis Kit (Cytiva) with the NotI-d(T)18 primer. Quantitative real-time PCR was carried out using TaqMan mRNA expression assays (Thermo Fisher Scientific, listed in Table S5) and TaqMan 2× Mastermix Plus–Low ROX (Eurogentec) on a ViiA 7 thermocycler (Thermo Fisher Scientific) using the standard settings provided by the manufacturer. The relative expression was determined by the comparative Ct method, and *GUSB* was used as the housekeeping gene.

Immunostaining

Differentiated cells were fixed in a solution of PBS containing 3.7% formaldehyde (Sigma-Aldrich) for 15 min, permeabilized in 0.1% Triton X-100 for 10 min (Sigma-Aldrich) and blocked with 10% fetal bovine serum (FBS; Thermo Fisher Scientific) for 1 h at room temperature (RT). Primary antibodies were diluted in a 10% FBS (Thermo Fisher Scientific) blocking solution and incubated overnight at 4°C. Alexa 488 or Alexa 594 conjugated secondary antibodies (diluted in 10% FBS) and Hoescht (1:1,000 dilution, ThermoFisher Scientific) were applied for 1–2 h at RT. Confocal images were acquired with an LSM800 confocal microscope (Carl Zeiss). For quantification, the positive cells were counted and compared to the number of Hoescht-stained nuclei to determine the percent positivity using ZEN 2 (blue edition) imaging software. The areas are randomly selected based on the Hoescht channel, and the positive cells are quantified by calculating the ratio of total positive cells to the total number of cells within those areas. The numbers of cells quantified for each image can be found in Table S6. The lists with antibodies can be found in Table S7.

In vitro differentiation of iPSCs

The neuroectoderm differentiation was based on Douvaras and Fossati (2015). In brief, 90% confluent hESCs were subjected to neural induction for 8 days using 100 nM RA (Sigma-Aldrich), 10 μM SB431542 (Tocris), and 250 nM LDN193189 (STEMCELL Technologies). The induction of cardiac progenitor differentiation was based on Lin and Zou (2020). hESCs at 80%–90% confluence were treated with 5 mM CHIR99021 (Tocris) for 24 h, after which the cells were cultured in medium with 0.6 U/mL heparin (Sigma-Aldrich) for 24 h. Subsequently, the medium was supplemented with 0.6 U/mL heparin and 3 mM IWP2 (Tocris) for another 3 days. The 8-day depatoblast differentiation was based on Boon et al. (2020). Differentiation was initiated at 40%–50% confluency, and the hESCs were treated with with 50 ng/mL activin A (STEMCELL Technologies), 50 ng/mL WNT3A (PeproTech), and

Figure 7. Downregulation of *SALL3* and loss of 18q result in the deregulation of genes in pathways associated with pluripotency and differentiation

(A and B) Unsupervised clustering heatmap (A) and PCA (B) of the coding genes with a count per million >1 in at least 2 samples.
(C–E) Volcano plots of differential gene expression analysis for VUB13^{del18q} vs. hESC^{WT} and hESC^{WT-NT}. The labeled genes are the top 20 commonly deregulated genes across the 3 comparisons (C), hESC^{WT_SALL3KD} vs. hESC^{WT} and hESC^{WT-NT} (D), and VUB13^{del18q_SALL3OE} vs. VUB13^{del18q} (E), with a cutoff value of $|\log_2\text{fold change}| > 1$ and FDR < 0.05.
(F) Venn diagrams show the genes downregulated in VUB13^{del18q_SALL3OE} and upregulated in VUB13^{del18q} and hESC^{WT_SALL3KD}.
(G) Venn diagrams showing the genes upregulated in VUB13^{del18q_SALL3OE} and downregulated in VUB13^{del18q} and hESC^{WT_SALL3KD}.
(H) Venn diagrams of the pathways in the H library common among VUB13^{del18q}, hESC^{WT_SALL3KD}, VUB13^{del18q_SALL3OE}, and hESC^{WT}.
(I) Venn diagrams of the pathways in the C2 library common among VUB13^{del18q}, hESC^{WT_SALL3KD}, VUB13^{del18q_SALL3OE}, and hESC^{WT}.
(J) Pathways commonly deregulated among the different samples are associated with pluripotency and differentiation.

6 μL/mL DMSO (Sigma-Aldrich) for 48 h. The cells were incubated for an additional 48 h in the same medium without WNT3A. Then, the medium was changed to contain 50 ng/mL BMP4 (STEMCELL Technologies) and 6 μL/mL DMSO for the following 4 days. More details on the differentiation and the media composition can be found in the supplemental information.

Generation of *SALL3* KD and OE cell lines

hESC^{WT_SALL3KD} cells were generated by infecting hESC^{WT} with lentiviral particles expressing *SALL3*-targeted shRNAs (SigmaMISSION shRNA targeting set TRCN0000019754, TRCN0000417790) or control shRNA plasmid. hESC^{del18q_SALL3OE} cells were generated by infecting hESC^{del18q} with lentiviral particles containing the pLVSIN-EF1α puromycin vector expressing *SALL3* (a gift from Yoji Sato, Division of Cell-Based Therapeutic Products, National Institute of Health Sciences, Japan).

RNA-seq

RNA-seq library preparation was performed using QuantSeq 3′ mRNA-Seq Library Prep Kits (Lexogen) following Illumina protocols. Sequencing was performed on a high-throughput Illumina NextSeq 500 flow cell. On average, $13.9 \times 10^6 \pm 7.1 \times 10^6$ paired-end reads per sample were uniquely mapped, with an average coverage per base of 101 paired reads. Details on the bioinformatic processing can be found in the supplemental information.

Statistics

All of the differentiation experiments were carried out in at least triplicate ($n \geq 3$). All of the data are presented as the mean ± SEM. Statistical evaluation of the differences between two groups was performed using unpaired two-tailed t tests in GraphPad Prism9 software, with $p < 0.05$ determined to indicate significance.

SUPPLEMENTAL INFORMATION

Supplemental information can be found online at https://doi.org/10.1016/j.stemcr.2024.03.001.

ACKNOWLEDGMENTS

The authors thank Yoji Sato from the Division of Cell-Based Therapeutic Products, National Institutes of Health Sciences in Japan, for kindly sharing the lentiviral construct for *SALL3* OE. Y.L. is a predoctoral fellow supported by the China Scholarship Council, and M.R., C.J., N.K. and E.C.d.D. are predoctoral fellows supported by the Fonds voor Wetenschappelijk Onderzoek Vlaanderen (FWO). This research was supported by the FWO (Fonds voor Wetenschappelijk Onderzoek – Vlaanderen; grant no. 1506617N) and the Methusalem Grant (to K.S., Vrije Universiteit Brussel).

AUTHOR CONTRIBUTIONS

Y.L. carried out all of the experiments and bioinformatics analysis unless stated otherwise and co-wrote the manuscript. D.A.D. cowrote the manuscript. N.K. packaged the lentivirus and assisted in transduction. E.C.d.D. assisted with the bioinformatics analysis. M.R. assisted in the microscopy and cell counting. C.J. and M.G. assisted with the cell culture. K.S. proofread the paper. C.S. cowrote the manuscript and designed and supervised the experimental work.

DECLARATION OF INTERESTS

The authors declare no competing interests.

Received: September 28, 2023
Revised: February 29, 2024
Accepted: March 1, 2024
Published: March 28, 2024

REFERENCES

International Stem Cell Initiative, Amps, K., Andrews, P.W., Anyfantis, G., Armstrong, L., Avery, S., Baharvand, H., Baker, J., Baker, D., Munoz, M.B., et al. (2011). Screening ethnically diverse human embryonic stem cells identifies a chromosome 20 minimal amplicon conferring growth advantage. Nat. Biotechnol. *29*, 1132–1144.

Andrews, P.W., Barbaric, I., Benvenisty, N., Draper, J.S., Ludwig, T., Merkle, F.T., Sato, Y., Spits, C., Stacey, G.N., Wang, H., and Pera, M.F. (2022). The consequences of recurrent genetic and epigenetic variants in human pluripotent stem cells. Cell Stem Cell *29*, 1624–1636.

Avery, S., Hirst, A.J., Baker, D., Lim, C.Y., Alagaratnam, S., Skotheim, R.I., Lothe, R.A., Pera, M.F., Colman, A., Robson, P., et al. (2013). BCL-XL Mediates the Strong Selective Advantage of a 20q11.21 Amplification Commonly Found in Human Embryonic Stem Cell Cultures. Stem Cell Rep. *1*, 379–386.

Avior, Y., Eggan, K., and Benvenisty, N. (2019). Cancer-Related Mutations Identified in Primed and Naive Human Pluripotent Stem Cells. Cell Stem Cell *25*, 456–461.

Baker, D.E.C., Harrison, N.J., Maltby, E., Smith, K., Moore, H.D., Shaw, P.J., Heath, P.R., Holden, H., and Andrews, P.W. (2007). Adaptation to culture of human embryonic stem cells and oncogenesis in vivo. Nat. Biotechnol. *25*, 207–215.

Bar, S., and Benvenisty, N. (2019). Epigenetic aberrations in human pluripotent stem cells. EMBO J *38*, e101033.

Baubec, T., Colombo, D.F., Wirbelauer, C., Schmidt, J., Burger, L., Krebs, A.R., Akalin, A., and Schübeler, D. (2015). Genomic profiling of DNA methyltransferases reveals a role for DNMT3B in genic methylation. Nature *520*, 243–247.

Bayindir, B., Dehaspe, L., Brison, N., Brady, P., Ardui, S., Kammoun, M., Van Der Veken, L., Lichtenbelt, K., Van Den Bogaert, K., Van Houdt, J., et al. (2015). Noninvasive prenatal testing using a novel analysis pipeline to screen for all autosomal fetal aneuploidies improves pregnancy management. Eur. J. Hum. Genet. *23*, 1286–1293.

Ben-David, U., Arad, G., Weissbein, U., Mandefro, B., Maimon, A., Golan-Lev, T., Narwani, K., Clark, A.T., Andrews, P.W., Benvenisty, N., and Carlos Biancotti, J. (2014). Aneuploidy induces profound changes in gene expression, proliferation and tumorigenicity of human pluripotent stem cells. Nat. Commun. *5*, 4825.

Boon, R., Kumar, M., Tricot, T., Elia, I., Ordovas, L., Jacobs, F., One, J., De Smedt, J., Eelen, G., Bird, M., et al. (2020). Amino acid levels

determine metabolism and CYP450 function of hepatocytes and hepatoma cell lines. Nat. Commun. *11*, 1393.

Chapman, G., Alsaqati, M., Lunn, S., Singh, T., Linden, S.C., Linden, D.E.J., van den Bree, M.B.M., Ziller, M., Owen, M.J., Hall, J., et al. (2022). Using induced pluripotent stem cells to investigate human neuronal phenotypes in 1q21.1 deletion and duplication syndrome. Mol. Psychiatry *27*, 819–830.

Douvaras, P., and Fossati, V. (2015). Generation and isolation of oligodendrocyte progenitor cells from human pluripotent stem cells. Nat. Protoc. *10*, 1143–1154.

Doyle, M.J., Lohr, J.L., Chapman, C.S., Koyano-Nakagawa, N., Garry, M.G., and Garry, D.J. (2015). Human Induced Pluripotent Stem Cell-Derived Cardiomyocytes as a Model for Heart Development and Congenital Heart Disease. Stem Cell Rev. Rep. *11*, 710–727.

Dutta, D., Ray, S., Home, P., Larson, M., Wolfe, M.W., and Paul, S. (2011). Self Renewal vs. Lineage Commitment of Embryonic Stem Cells: Protein Kinase C Signaling Shifts the Balance. Stem Cell. *29*, 618–628.

Fazeli, A., Liew, C.-G., Matin, M.M., Elliott, S., Jeanmeure, L.F.C., Wright, P.C., Moore, H., and Andrews, P.W. (2011). Altered patterns of differentiation in karyotypically abnormal human embryonic stem cells. Int. J. Dev. Biol. *55*, 175–180.

Geens, M., Seriola, A., Barbé, L., Santalo, J., Veiga, A., Dée, K., Van Haute, L., Sermon, K., and Spits, C. (2016). Female human pluripotent stem cells rapidly lose X chromosome inactivation marks and progress to a skewed methylation pattern during culture. Mol. Hum. Reprod. *22*, 285–298.

Halliwell, J.A., Frith, T.J.R., Laing, O., Price, C.J., Bower, O.J., Stavish, D., Gokhale, P.J., Hewitt, Z., El-Khamisy, S.F., Barbaric, I., and Andrews, P.W. (2020). Nucleosides Rescue Replication-Mediated Genome Instability of Human Pluripotent Stem Cells. Stem Cell Rep. *14*, 1009–1017.

Hamid, T., Xu, Y., Ismahil, M.A., Li, Q., Jones, S.P., Bhatnagar, A., Bolli, R., and Prabhu, S.D. (2016). TNF receptor signaling inhibits cardiomyogenic differentiation of cardiac stem cells and promotes a neuroadrenergic-like fate. Am. J. Physiol. Heart Circ. Physiol. *311*, H1189–H1201.

Van Haute, L., Spits, C., Geens, M., Seneca, S., and Sermon, K. (2013). Human embryonic stem cells commonly display large mitochondrial DNA deletions. Nat. Biotechnol. *31*, 20–23.

Hogendorf, A., Zieliński, M., Constantinou, M., Śmigiel, R., Wierzba, J., Wyka, K., Wędrychowicz, A., Jakubiuk-Tomaszuk, A., Budzynska, E., Piotrowicz, M., et al. (2021). Immune Dysregulation in Patients With Chromosome 18q Deletions—Searching for Putative Loci for Autoimmunity and Immunodeficiency. Front. Immunol. *12*, 742834.

Jacobs, K., Mertzanidou, A., Geens, M., Nguyen, H.T., Staessen, C., and Spits, C. (2014). Low-grade chromosomal mosaicism in human somatic and embryonic stem cell populations. Nat. Commun. *5*, 4227.

Jacobs, K., Zambelli, F., Mertzanidou, A., Smolders, I., Geens, M., Nguyen, H.T., Barbé, L., Sermon, K., and Spits, C. (2016). Higher-Density Culture in Human Embryonic Stem Cells Results in DNA Damage and Genome Instability. Stem Cell Rep. *6*, 330–341.

Jo, H.Y., Lee, Y., Ahn, H., Han, H.J., Kwon, A., Kim, B.Y., Ha, H.Y., Kim, S.C., Kim, J.H., Kim, Y.O., et al. (2020). Functional in vivo and in vitro effects of 20q11.21 genetic aberrations on hPSC differentiation. Sci. Rep. *10*, 18582.

Keller, A., Dziedzicka, D., Zambelli, F., Markouli, C., Sermon, K., Spits, C., and Geens, M. (2018). Genetic and epigenetic factors which modulate differentiation propensity in human pluripotent stem cells. Hum. Reprod. Update *24*, 162–175.

Keller, A., Tilleman, L., Dziedzicka, D., Zambelli, F., Sermon, K., Van Nieuwerburgh, F., Spits, C., and Geens, M. (2019). Uncovering low-level mosaicism in human embryonic stem cells using high throughput single cell shallow sequencing. Sci. Rep. *9*, 14844.

Kilpinen, H., Goncalves, A., Leha, A., Afzal, V., Alasoo, K., Ashford, S., Bala, S., Bensaddek, D., Casale, F.P., Culley, O.J., et al. (2017). Common genetic variation drives molecular heterogeneity in human iPSCs. Nature *546*, 370–375.

Kobold, S., Guhr, A., Mah, N., Bultjer, N., Seltmann, S., Seiler Wulczyn, A.E.M., Stacey, G., Jie, H., Liu, W., Löser, P., and Kurtz, A. (2020). A Manually Curated Database on Clinical Studies Involving Cell Products Derived from Human Pluripotent Stem Cells. Stem Cell Rep. *15*, 546–555.

Kuroda, T., Yasuda, S., Tachi, S., Matsuyama, S., Kusakawa, S., Tano, K., Miura, T., Matsuyama, A., and Sato, Y. (2019). SALL3 expression balance underlies lineage biases in human induced pluripotent stem cell differentiation. Nat. Commun. *10*, 2175.

Kurpinski, K., Lam, H., Chu, J., Wang, A., Kim, A., Tsay, E., Agrawal, S., Schaffer, D.V., and Li, S. (2010). Transforming Growth Factor-β and Notch Signaling Mediate Stem Cell Differentiation into Smooth Muscle Cells. Stem Cell. *28*, 734–742.

Kushnir, M., Dresner, E., Mandel, S., and Gozes, I. (2008). Silencing of the ADNP-family member, ADNP2, results in changes in cellular viability under oxidative stress. J. Neurochem. *105*, 537–545.

Lin, Y., and Zou, J. (2020). Differentiation of Cardiomyocytes from Human Pluripotent Stem Cells in Fully Chemically Defined Conditions. STAR Protoc. *1*, 100015.

Maitra, A., Arking, D.E., Shivapurkar, N., Ikeda, M., Stastny, V., Kassauei, K., Sui, G., Cutler, D.J., Liu, Y., Brimble, S.N., et al. (2005). Genomic alterations in cultured human embryonic stem cells. Nat. Genet. *37*, 1099–1103.

Markouli, C., Couvreu De Deckersberg, E., Regin, M., Nguyen, H.T., Zambelli, F., Keller, A., Dziedzicka, D., De Kock, J., Tilleman, L., Van Nieuwerburgh, F., et al. (2019). Gain of 20q11.21 in Human Pluripotent Stem Cells Impairs TGF-β-Dependent Neuroectodermal Commitment. Stem Cell Rep. *13*, 163–176.

Mateizel, I., De Temmerman, N., Ullmann, U., Cauffman, G., Sermon, K., Van de Velde, H., De Rycke, M., Degreef, E., Devroey, P., Liebaers, I., and Van Steirteghem, A. (2006). Derivation of human embryonic stem cell lines from embryos obtained after IVF and after PGD for monogenic disorders. Hum. Reprod. *21*, 503–511.

Mateizel, I., Spits, C., De Rycke, M., Liebaers, I., and Sermon, K. (2010). Derivation, culture, and characterization of VUB hESC lines. In Vitro Cell. Dev. Biol. Anim. *46*, 300–308.

Merkle, F.T., Ghosh, S., Kamitaki, N., Mitchell, J., Avior, Y., Mello, C., Kashin, S., Mekhoubad, S., Ilic, D., Charlton, M., et al. (2017).

Human pluripotent stem cells recurrently acquire and expand dominant negative P53 mutations. Nature *545*, 229–233.

Merkle, F.T., Ghosh, S., Genovese, G., Handsaker, R.E., Kashin, S., Meyer, D., Karczewski, K.J., O'Dushlaine, C., Pato, C., Pato, M., et al. (2022). Whole-genome analysis of human embryonic stem cells enables rational line selection based on genetic variation. Cell Stem Cell *29*, 472–486.e7.

Nguyen, H.T., Geens, M., Mertzanidou, A., Jacobs, K., Heirman, C., Breckpot, K., and Spits, C. (2014). Gain of 20q11.21 in human embryonic stem cells improves cell survival by increased expression of Bcl-xL. Mol. Hum. Reprod. *20*, 168–177.

Olariu, V., Harrison, N.J., Coca, D., Gokhale, P.J., Baker, D., Billings, S., Kadirkamanathan, V., and Andrews, P.W. (2010). Modeling the evolution of culture-adapted human embryonic stem cells. Stem Cell Res. *4*, 50–56.

Ott, T., Kaestner, K.H., Monaghan, A.P., and Schütz, G. (1996). The mouse homolog of the region specific homeotic gene spalt of Drosophila is expressed in the developing nervous system and in mesoderm-derived structures. Mech. Dev. *56*, 117–128.

Park, K.S. (2011). TGF-beta Family Signaling in Embryonic Stem Cells. Int. J. Stem Cells *4*, 18–23.

Price, C.J., Stavish, D., Gokhale, P.J., Stevenson, B.A., Sargeant, S., Lacey, J., Rodriguez, T.A., and Barbaric, I. (2021). Genetically variant human pluripotent stem cells selectively eliminate wild-type counterparts through YAP-mediated cell competition. Dev. Cell *56*, 2455–2470.e10.

Quevedo, M., Meert, L., Dekker, M.R., Dekkers, D.H.W., Brandsma, J.H., van den Berg, D.L.C., Ozgür, Z., van IJcken, W.F.J., Demmers, J., Fornerod, M., et al. (2019). Mediator complex interaction partners organize the transcriptional network that defines neural stem cells. Nat. Commun. *10*, 2669.

Shikauchi, Y., Saiura, A., Kubo, T., Niwa, Y., Yamamoto, J., Murase, Y., and Yoshikawa, H. (2009). SALL3 Interacts with DNMT3A and Shows the Ability To Inhibit CpG Island Methylation in Hepatocellular Carcinoma. Mol. Cell Biol. *29*, 1944–1958.

Spits, C., Mateizel, I., Geens, M., Mertzanidou, A., Staessen, C., Vandeskelde, Y., Van Der Elst, J., Liebaers, I., and Sermon, K. (2008). Recurrent chromosomal abnormalities in human embryonic stem cells. Nat. Biotechnol. *26*, 1361–1363.

Taylor, A.M., Shih, J., Ha, G., Gao, G.F., Zhang, X., Berger, A.C., Schumacher, S.E., Wang, C., Hu, H., Liu, J., et al. (2018). Genomic and Functional Approaches to Understanding Cancer Aneuploidy. Cancer Cell *33*, 676–689.e3.

Varela, C., Denis, J.A., Polentes, J., Feyeux, M., Aubert, S., Champon, B., Piétu, G., Peschanski, M., and Lefort, N. (2012). Recurrent genomic instability of chromosome 1q in neural derivatives of human embryonic stem cells. J. Clin. Invest. *122*, 569–574.

Vitillo, L., Anjum, F., Hewitt, Z., Stavish, D., Laing, O., Baker, D., Barbaric, I., and Coffey, P. (2023). The isochromosome 20q abnormality of pluripotent cells interrupts germ layer differentiation. Stem Cell Rep. *18*, 782–797.

Weinberg, D.N., Papillon-Cavanagh, S., Chen, H., Yue, Y., Chen, X., Rajagopalan, K.N., Horth, C., McGuire, J.T., Xu, X., Nikbakht, H., et al. (2019). The histone mark H3K36me2 recruits DNMT3A and shapes the intergenic DNA methylation landscape. Nature *573*, 281–286.

Weinberger, L., Ayyash, M., Novershtern, N., and Hanna, J.H. (2016). Dynamic stem cell states: naive to primed pluripotency in rodents and humans. Nat. Rev. Mol. Cell Biol. *17*, 155–169.

Werbowetski-Ogilvie, T.E., Bossé, M., Stewart, M., Schnerch, A., Ramos-Mejia, V., Rouleau, A., Wynder, T., Smith, M.J., Dingwall, S., Carter, T., et al. (2009). Characterization of human embryonic stem cells with features of neoplastic progression. Nat. Biotechnol. *27*, 91–97.

Wood, K.A., Ellingford, J.M., Thomas, H.B., Genomics UK Research Consortium, Douzgou, S., Beaman, G.M., Hobson, E., O'Keefe, R.T., O'Keefe, R.T., and Newman, W.G. (2022). Expanding the genotypic spectrum of TXNL4A variants in Burn-McKeown syndrome. Clin. Genet. *101*, 255–259.

Wuu, Y.D., Pampfer, S., Vanderheyden, I., Lee, K.H., and De Hertogh, R. (1998). Impact of tumor necrosis factor alpha on mouse embryonic stem cells. Biol. Reprod. *58*, 1416–1424.

Yamanaka, S. (2020). Pluripotent Stem Cell-Based Cell Therapy—Promise and Challenges. Cell Stem Cell *27*, 523–531.

Zambelli, F., Mertens, J., Dziedzicka, D., Sterckx, J., Markouli, C., Keller, A., Tropel, P., Jung, L., Viville, S., Van de Velde, H., et al. (2018). Random Mutagenesis, Clonal Events, and Embryonic or Somatic Origin Determine the mtDNA Variant Type and Load in Human Pluripotent Stem Cells. Stem Cell Rep. *11*, 102–114.

Zhang, J., Tao, R., Campbell, K.F., Carvalho, J.L., Ruiz, E.C., Kim, G.C., Schmuck, E.G., Raval, A.N., da Rocha, A.M., Herron, T.J., et al. (2019). Functional cardiac fibroblasts derived from human pluripotent stem cells via second heart field progenitors. Nat. Commun. *10*, 2238.

Stem Cell Reports, Volume *19*

Supplemental Information

SALL3 mediates the loss of neuroectodermal differentiation potential in human embryonic stem cells with chromosome 18q loss

Yingnan Lei, Diana Al Delbany, Nuša Krivec, Marius Regin, Edouard Couvreu de Deckersberg, Charlotte Janssens, Manjusha Ghosh, Karen Sermon, and Claudia Spits

Supplementary table 1. Passage range and karyotype of the lines in this study

Line	Passage range	Karyotype	Method of karyotyping and size in Mb
VUB14^{wt}	51-55	46, XX	Shallow whole genome sequencing
VUB04^{wt}	16-23	46, XX	Shallow whole genome sequencing
VUB03^{wt}	19-25	46, XX	Shallow whole genome sequencing
VUB13^{del18q}	52-58	46, XX<br>dup(5) (q21.3qter)<br>del(18) (q21.2qter)	Shallow whole genome sequencing<br>chr5: 33 Mb<br>chr18: 29 Mb
VUB14^{del18q}	49-54	46, XX,<br>dup(7)(p22.3pter)<br>del(18)(q21.32qter)	Shallow whole genome sequencing<br>chr7: 32 Mb<br>chr18: 21 Mb
VUB04	29	46, XX	Array-based comparative genomic hybridization (published in Spits et al., 2008)
	66	46, XX,<br>dup(5)(q14.2qter),<br>del(18)(q21.2qter)	Array-based comparative genomic hybridization (published in Spits et al., 2008)<br>Chr5: 99.5 Mb<br>Chr18: 28.7 Mb
VUB26	10	46, XX,<br>dup(7)(q33qter),<br>del(18)(q23qter)	Array-based comparative genomic hybridization (published in Spits et al., 2008)<br>Chr7: 11.3 Mb<br>Chr18: 4.6 Mb

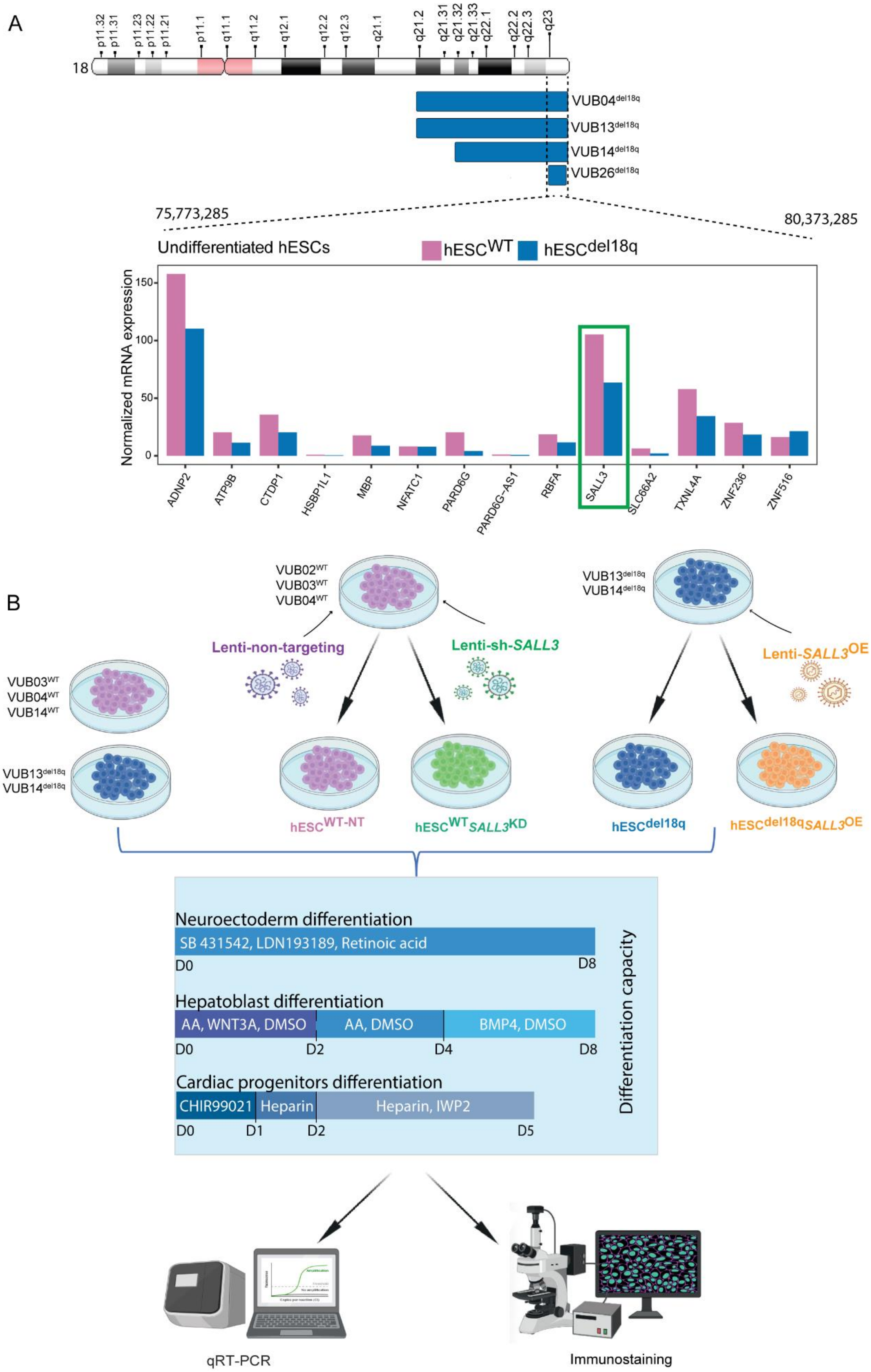
A
18
VUB04del18q
VUB13del18q
VUB14del18q
VUB26del18q
75,773,285
80,373,285
Undifferentiated hESCs
hESCWT
hESCdel18q
Normalized mRNA expression
ADNP2
ATP9B
CTDP1
HSBP1L1
MBP
NFATC1
PARD6G
PARD6G-AS1
RBFA
SALL3
SLC66A2
TXNL4A
ZNF236
ZNF516
B
VUB02WT
VUB03WT
VUB04WT
VUB13del18q
VUB14del18q
Lenti-non-targeting
Lenti-sh-SALL3
Lenti-SALL3OE
VUB03WT
VUB04WT
VUB14WT
VUB13del18q
VUB14del18q
hESCWT-NT
hESCWT SALL3KD
hESCdel18q
hESCdel18q SALL3OE
Neuroectoderm differentiation
SB 431542, LDN193189, Retinoic acid
D0
D8
Hepatoblast differentiation
AA, WNT3A, DMSO
AA, DMSO
BMP4, DMSO
D0
D2
D4
D8
Cardiac progenitors differentiation
CHIR99021
Heparin
Heparin, IWP2
D0
D1
D2
D5
Differentiation capacity
qRT-PCR
Immunostaining

Supplementary figure 1. The minimal common region of 18q loss spans 14 genes expressed in undifferentiated hESCs, including *SALL3*. (A) Graphical representation of the regions of chromosome 18 loss in all VUB hESC^{del18q} lines and showing the normalized expression of the genes located in the minimal common region of 18q loss examined by bulk-RNA sequencing to compare the transcriptome of hESC^{del18q} and hESC^{WT} as well as the genetically modified counterparts. (B) Overview of the experimental setup. We used two lines carrying a loss of 18q and three genetically balanced lines. The WT lines were also genetically modified to down-regulate *SALL3*, while 18q loss lines were modified to over-express *SALL3*. All lines were subjected to differentiate to neuroectoderm, hepatoblasts and cardiac progenitors, all differentiation experiments were carried out at least in triplicate. qRT-PCR and immunostaining were used to assess the identity of the differentiated cells. AA: Activin A; Lenti-sh-*SALL3*: lentiviral transduction for a short-hairpin RNA against *SALL3.* Lenti-*SALL3*-OE: lentiviral transduction with a construct for the transgenic over-expression of *SALL3.*

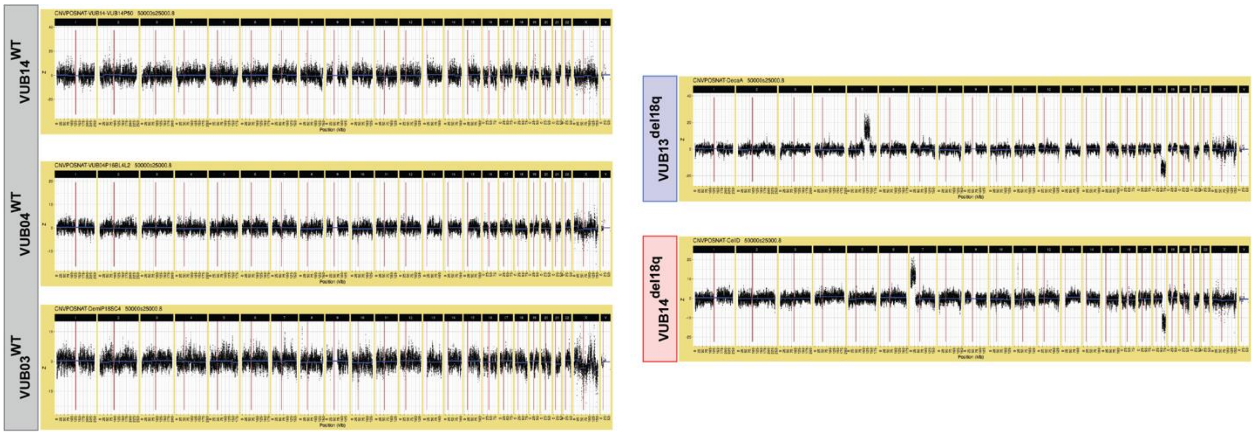

Supplementary figure 2. Shallow sequencing results of the lines used in this study

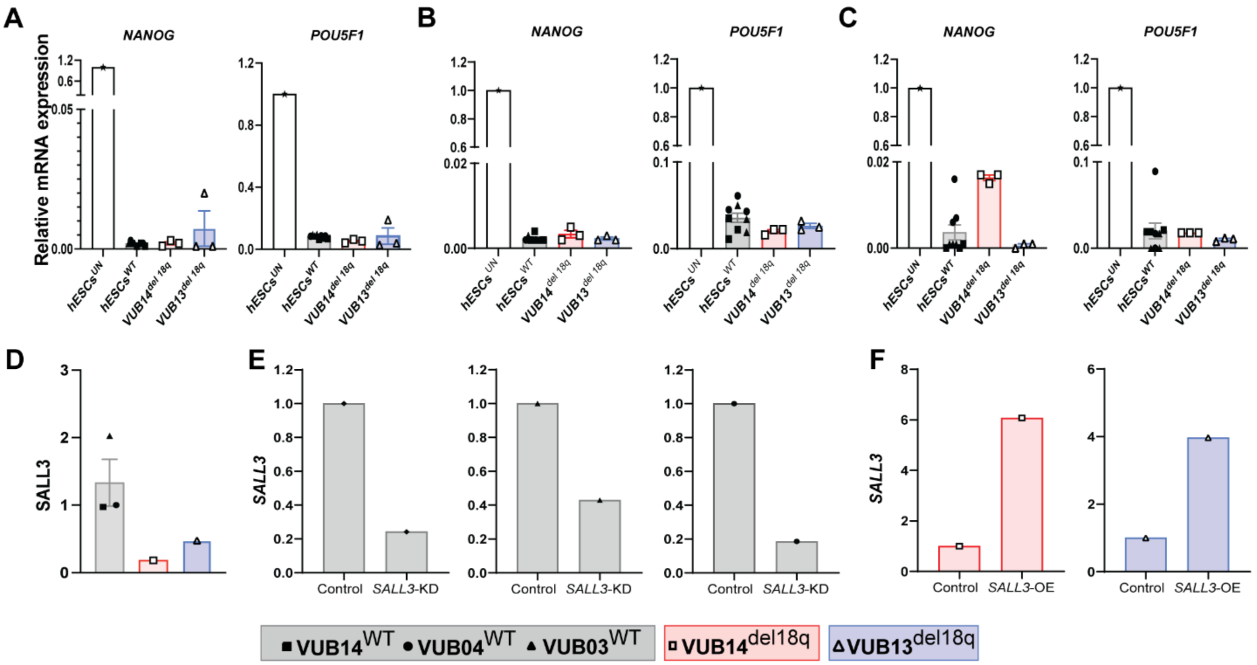

Supplementary figure 3. The relative mRNA expression of undifferentiated state markers during the differentiation (A-C) **and the relative *SALL3* mRNA expression** (D-F). Relative mRNA expression as measured by qRT-PCR for pluripotency markers *NANOG*, *POU5F1* during the differentiation process (n = 3). Data are shown as means ± SEM, each pattern indicates different cell lines. Neuroectoderm (A) cardiac progenitor (B) hepatoblasts (C). Relative *SALL3* mRNA expression as measured by qRT-PCR between hESC^{del18q} compared to hESC^{WT} (D). Relative *SALL3* mRNA expression as measured by qRT-PCR between hESC$^{WT\text{-}NT}$ and hESC^{WT_SALL3KD} (E). Relative *SALL3* mRNA expression as measured by qRT-PCR between hESC^{del18q} and hESC^{del18q_SALL3OE} (F)

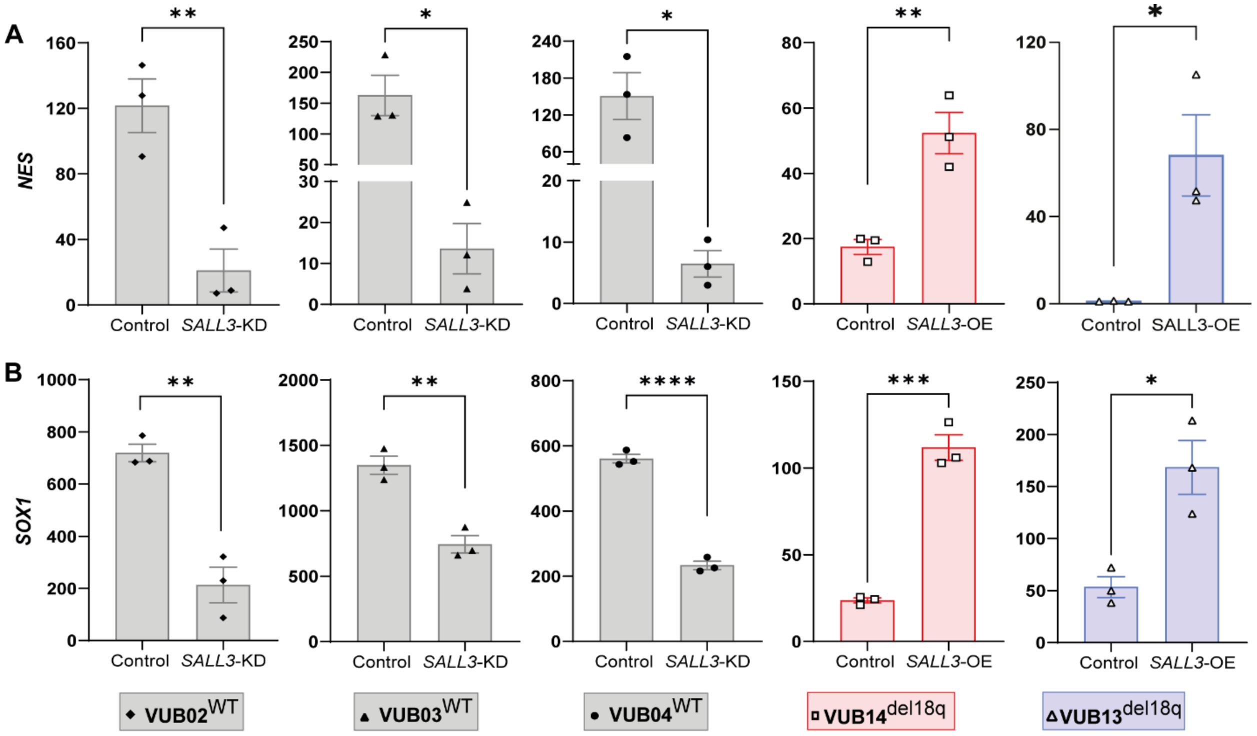

Supplementary Figure 4. *SALL3* drives the impaired neuroectoderm differentiation (A-B) Relative mRNA expression for neuroectoderm markers (n = 3) by qRT-PCR. Different patterns indicate different cell lines, and the horizontal bars with asterisks *, **, *** and **** represent statistical significance between samples at 5%, 1%, 0.1% and 0.01% respectively (unpaired t-test). *NES* (A), *SOX1*(B).

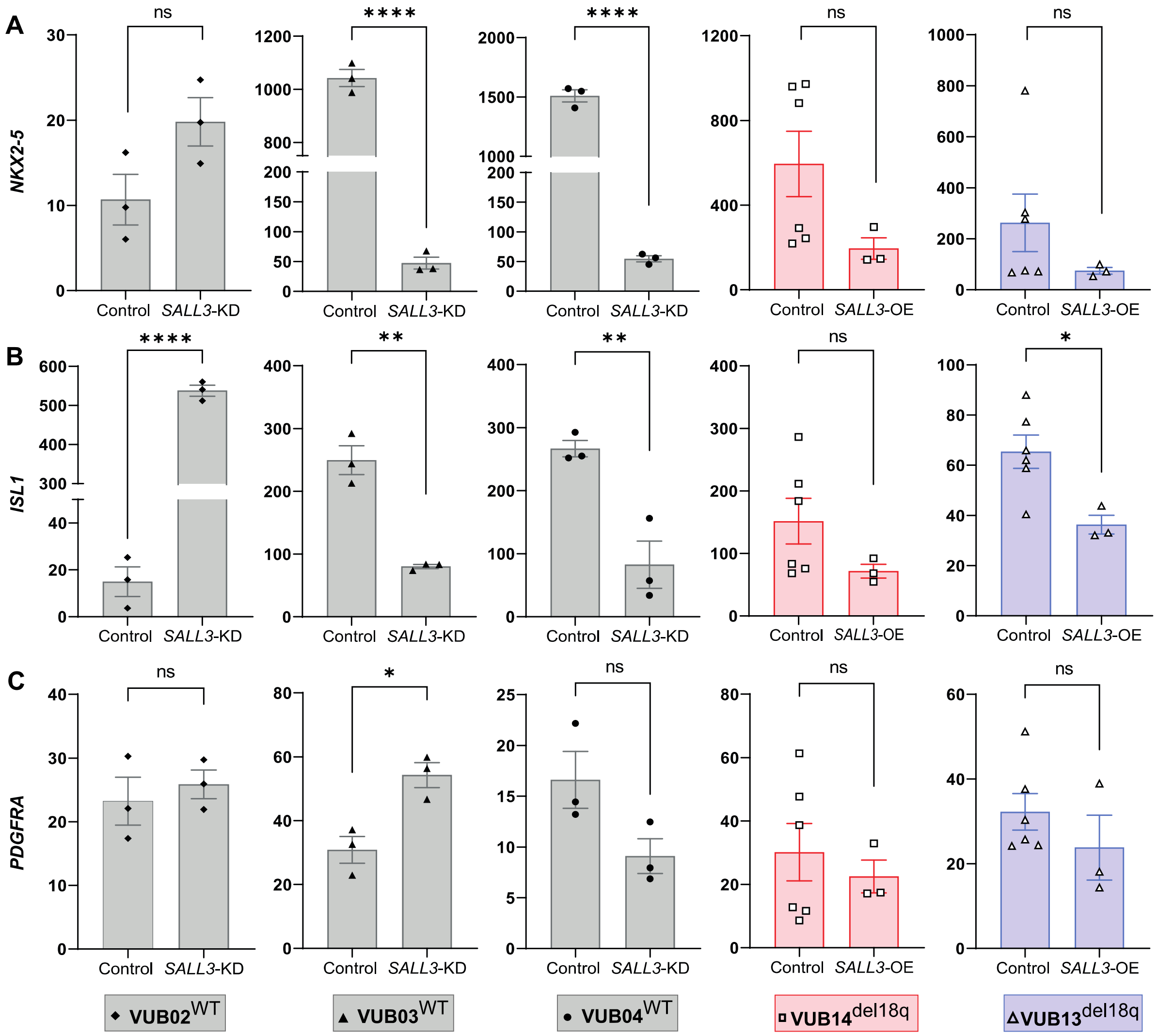

Supplementary Figure 5. *SALL3* is not affecting the cells ability to generate mesoderm (A-C) Relative mRNA expression for mesoderm markers measured by qRT-PCR (n = 3). Data are shown as the means ± SEM. Different patterns indicate different cell lines, and the horizontal bars with asterisks *, **, *** and **** represent statistical significance between samples at 5%, 1%, 0.1% and 0.01% respectively (unpaired t-test). *NKX2-5*(A), *ISL1*(B), *PDGFRA*(C).

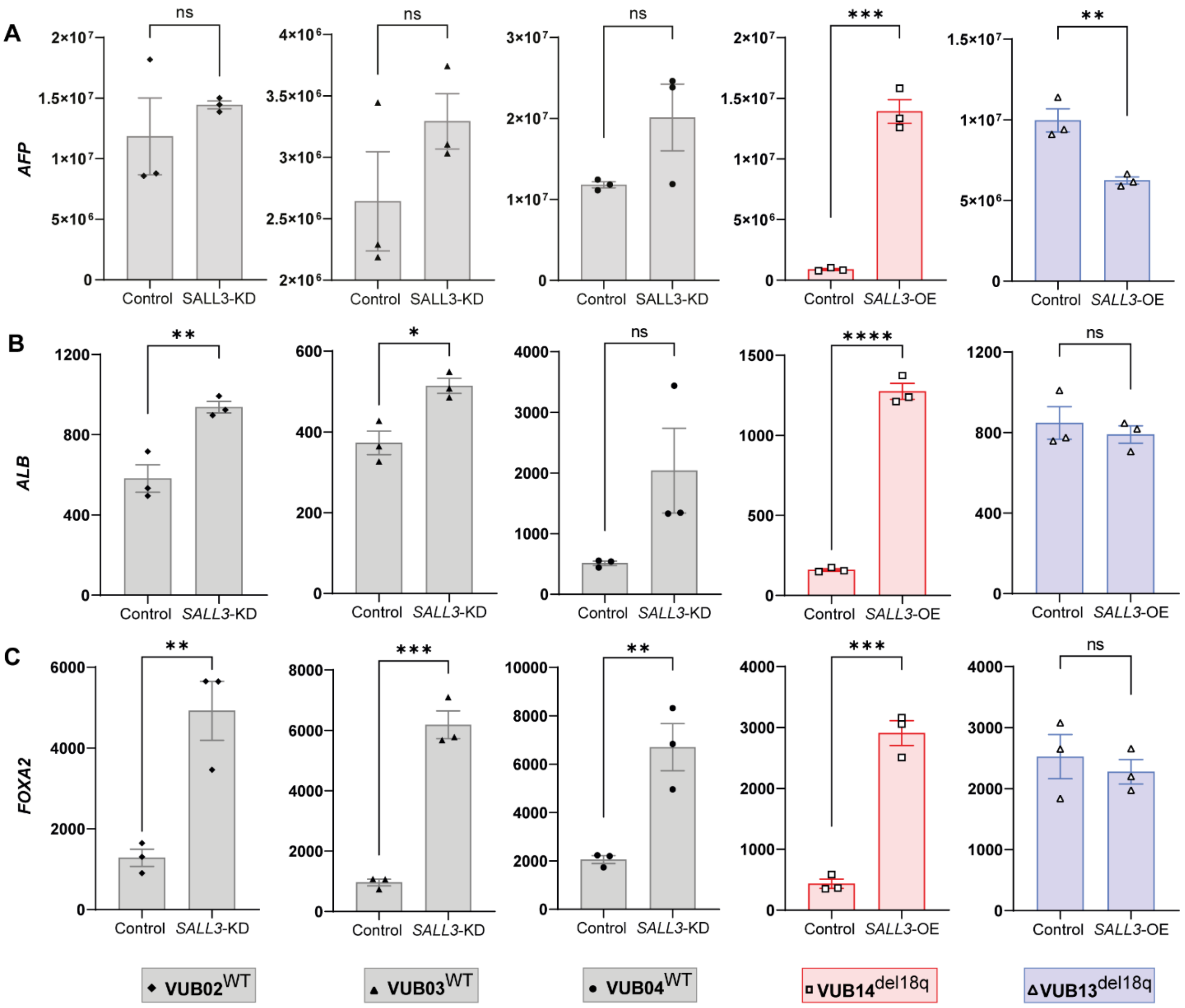

Supplementary Figure 6. *SALL3* is affecting the cells ability to generate endoderm (A-C) Relative mRNA expression for endoderm markers (n = 3) by qRT-PCR. Data are shown as the means ± SEM. Different patterns indicate different cell lines, and the horizontal bars with asterisks *, **, *** and **** represent statistical significance between samples at 5%, 1%, 0.1% and 0.01% respectively (unpaired t-test). *AFP*(A), *ALB*(B), *FOXA2*(C).

A

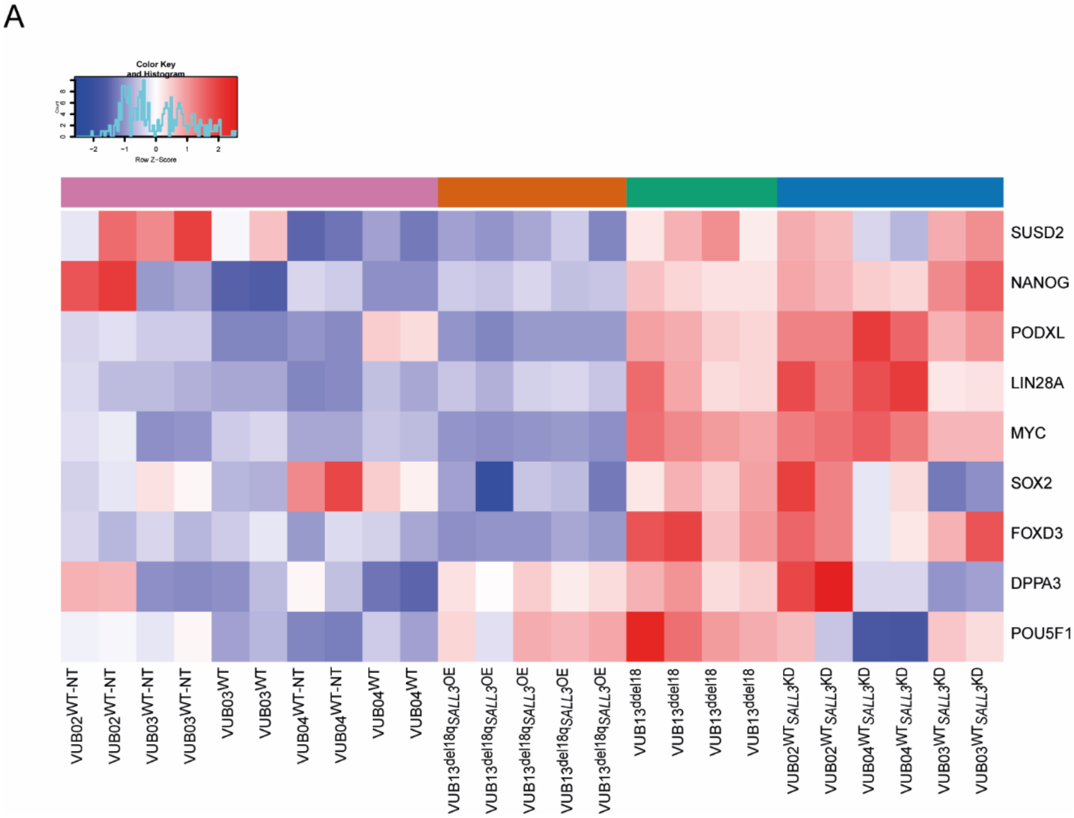

Supplementary Figure 7. Global transcriptomic profile analysis (A) Heatmap of pluripotency-associated genes

Supplementary methods

Definitive neuroectoderm specification

The protocol for inducing neuroectoderm differentiation was adapted from the protocol described by Douvaras and colleagues(Douvaras and Fossati, 2015). In brief, hESCs were seeded on Biolaminin 521 (Biolamina®)-coated 24-well plates at a ratio of 100,000 cells per cm^2 and grown to 90% confluence. Then, differentiation was induced by incubating the hESCs with neural induction specification medium for up to 8 days with daily medium changes. The neural induction specification medium consisted of basal medium supplemented with the following differentiation factors: 100 nM retinoic acid (Sigma-Aldrich), 10 µM SB431542 (Tocris), and 250 nM LDN193189 (STEMCELL Technologies). The basal medium was prepared by mixing DMEM/F12 (Thermo Scientific) basic medium supplemented with 1x NEAA (Thermo Scientific), 1x GlutaMAX (Thermo Scientific), 1x 2-mercaptoethanol (Thermo Scientific), 25 µg/mL insulin (Sigma-Aldrich) and 1x penicillin/streptomycin (P/S) (Thermo Scientific).

Cardiac progenitor differentiation

The induction of cardiac progenitor differentiation was initiated using a slightly modified version of the protocol from a previous publication(Lin and Zou, 2020). hESCs were seeded on Biolaminin-521-coated 24-well plates at a density of 100,000 cells/cm^2 and allowed to reach 80–90% confluence. At this point, differentiation was initiated by treating the hESCs with cardiomyocyte differentiation basal medium (CDBM) with 5 mM CHIR99021 (Tocris) to activate Wnt/β-catenin signaling and form the mesendoderm layer. After 24 h, CHIR99021 was removed, and the cells were cultured in CDBM with 0.6 U/mL heparin (Sigma-Aldrich) for 24 h. Subsequently, the CDBM medium was supplemented with 0.6 U/ml heparin and 3 mM IWP2 (Tocris) for another 3-day incubation with medium refreshed daily. The cardiomyocyte differentiation basal medium (CDBM) was composed of 10 µg/ml transferrin (Sigma Aldrich), 1x chemically defined lipid concentrate (Thermo Scientific) and E8 basal medium. The E8 medium was composed of DMEM/F12 (Thermo Scientific) basic medium with 64 mg/L L-ascorbic acid (Sigma-Aldrich) and 13.6 µg/L sodium selenium (Sigma-Aldrich).

Hepatoblast differentiation

Differentiation of hESCs into hepatoblasts was conducted using a protocol based on(Boon et al., 2020). The hESCs were seeded at a density of $5x10^4$ cells/cm^2 on 24-well plates precoated with Biolaminin-521. Upon reaching approximately 40-50% confluency, the hESCs were treated with liver differentiation medium (LDM) along with 50 ng/mL Activin A (STEMCELL Technologies), 50 ng/mL WNT3A (PeproTech) and 6 µL/mL DMSO (Sigma-Aldrich) for 48 h. The cells were incubated for an additional 48 h in the same medium without WNT3A. Then, the medium was changed to LDM with 50 ng/mL BMP4 (STEMCELL Technologies) and 6 µL/mL DMSO for the following 4 days, with the medium changed every two days. The samples were collected on day 8. LDM medium was created by adding MCDB 201 medium (pH=7.2, Sigma-Aldrich), L-ascorbic acid (Sigma-Aldrich), insulin-transferrin-selenium (ITS-G, Thermo Scientific),

linoleic acid-albumin from bovine serum albumin (LA-BSA, Sigma-Aldrich), 2-mercaptoethanol (Thermo scientific), and dexamethasone (Sigma-Aldrich) to DMEM high glucose medium (Westburg Life Sciences).

RNA sequencing

The FastQC algorithm(Love et al., 2014) was used to perform quality control on the raw sequence reads prior to the downstream analysis. The raw reads were aligned to the new version of the human Ensembl reference genome (GRCh38.p13) with Ensembl (GRCh38.83gtf) annotation using STAR version 2.5.3 in 2-pass mode(Dobin et al., 2013).The aligned reads were then quantified, and transcript abundances were estimated using RNA-seq by expectation maximization (RSEM, version 1.3.3)(Li and Dewey, 2011).

The count matrices were imported into R software (version 3.3.2) for further processing. The edgeR(Robinson et al., 2010) package was utilized to identify differentially expressed genes (DEGs) between groups. Transcripts with a count per million (cpm) greater than 1 in at least two samples were considered for the downstream analysis. Genes with a log2-fold change greater than 1 or less than -1 and a false discovery rate (FDR)-adjusted P-value less than 0.05 were considered significantly differentially expressed. Volcano plots of DEGs were generated using the ggplot2(Wickham, 2016) package in R, while Venn diagrams using VennDiagram function were used to visualize the overlap of the DEGs among different individuals. Principal component analysis (PCA) and heatmap clustering were performed using normalized counts and R packages. The heatmap was generated using the heatmap.2 funtion. PCA was performed using the prcomp function and plotted using ggplot2. Gene set enrichment analysis (GSEA) was applied to detect the enrichment of pathways using the GSEA function in R with the MSigDB C2 and H databases. Values are ranked by sign(logFC)*(-log10(FDR)). |NES| > 1 and p-value < 0.05 were considered the thresholds for significance for the gene sets.

References

Boon, R., Kumar, M., Tricot, T., Elia, I., Ordovas, L., Jacobs, F., One, J., De Smedt, J., Eelen, G., Bird, M., et al. (2020). Amino acid levels determine metabolism and CYP450 function of hepatocytes and hepatoma cell lines. Nature Communications 2020 11:1 *11*, 1–16. https://doi.org/10.1038/s41467-020-15058-6.

Dobin, A., Davis, C.A., Schlesinger, F., Drenkow, J., Zaleski, C., Jha, S., Batut, P., Chaisson, M., and Gingeras, T.R. (2013). STAR: ultrafast universal RNA-seq aligner. Bioinformatics *29*, 15. https://doi.org/10.1093/BIOINFORMATICS/BTS635.

Douvaras, P., and Fossati, V. (2015). Generation and isolation of oligodendrocyte progenitor cells from human pluripotent stem cells. Nature Protocols 2015 10:8 *10*, 1143–1154. https://doi.org/10.1038/nprot.2015.075.

Li, B., and Dewey, C.N. (2011). RSEM: Accurate transcript quantification from RNA-Seq data with or without a reference genome. BMC Bioinformatics *12*, 1–16. https://doi.org/10.1186/1471-2105-12-323/TABLES/6.

Lin, Y., and Zou, J. (2020). Differentiation of Cardiomyocytes from Human Pluripotent Stem Cells in Fully Chemically Defined Conditions. STAR Protoc *1*. https://doi.org/10.1016/J.XPRO.2020.100015.

Love, M.I., Huber, W., and Anders, S. (2014). Moderated estimation of fold change and dispersion for RNA-seq data with DESeq2. Genome Biol *15*, 1–21. https://doi.org/10.1186/S13059-014-0550-8/FIGURES/9.

Robinson, M.D., McCarthy, D.J., and Smyth, G.K. (2010). edgeR: a Bioconductor package for differential expression analysis of digital gene expression data. Bioinformatics *26*, 139–140. https://doi.org/10.1093/BIOINFORMATICS/BTP616.

Wickham, H. (2016). ggplot2. https://doi.org/10.1007/978-3-319-24277-4.

Supplementary table 5

TaqMan Gene Expression Assays

Gene	Supplier	Catalog number
GUSB	Applied Biosystems™	4326320E
PAX6	Thermo Scientific	Hs0024087
SOX1	Thermo Scientific	Hs01057642
NES	Thermo Scientific	Hs04187831
AFP	Thermo Scientific	Hs00173490
HNF4A	Thermo Scientific	Hs00604435
FOXA2	Thermo Scientific	Hs00232764
ALB	Thermo Scientific	Hs00609411
GATA4	Thermo Scientific	Hs01034629
NKX2-5	Thermo Scientific	Hs00231763
PDGFRA	Thermo Scientific	Hs00998018
ISL1	Thermo Scientific	Hs00158126
POU5F1	Thermo Scientific	Hs00742896
NANOG	Thermo Scientific	Hs02387400

TaqMan Copy Number Variation Assay

Gene	Supplier	Catalog number	Chromosome
RNaseP	Thermo Scientific	4403326	reference
ID1	Thermo Scientific	Hs01892845	20q
KIF14	Thermo Scientific	Hs00637799	1q
NANOG	Thermo Scientific	Hs03820140	12p
NMT1	Thermo Scientific	Hs0550678	17q

Supplementary table 6. Numbers of cells quantified for each image.

**Figure 1, Neuroectoderm**	Each datapoint shown in 1C represents the average of the quantification of 3 randomly taken images of the same well: VUB14^{wt} N= 21907 cells, VUB04^{wt} N= 22399 cells, VUB03^{wt} N= 22696 cells, VUB14^{del18q} N= 10882 cells, VUB13^{18qdel} N= 13462 cells.
**Figure 2, Cardiac progenitors**	Each datapoint shown in 2C represents the average of the quantification of 5 randomly taken images of the same well: VUB14^{wt} N= 25513 cells, VUB04^{wt} N= 27758 cells, VUB03^{wt} N= 24031 cells, VUB14^{del18q} N= 25348 cells, VUB13^{18qdel} N= 23857 cells.
**Figure 3, Hepatoblasts**	Each datapoint shown in 3C represents the average of the quantification of 5 randomly taken images of the same well: VUB14^{wt} N= 15325 cells, VUB04^{wt} N= 12171 cells, VUB03^{wt} N= 11816 cells, VUB14^{del18q} N= 21692 cells, VUB13^{del18q} N= 17302 cells
**Figure 4, Neuroectoderm**	Each datapoint shown in 4C represents the average of the quantification of 3 randomly taken images of the same well: VUB04^{WT-NT} N= 23280 cells, VUB04^{WT_SALL3KD} N= 16441 cells, VUB03^{WT-NT} N= 21189 cells, VUB03^{WT_SALL3KD} N= 20844 cells, VUB02^{WT-NT} N= 23952 cells, VUB02^{WT_SALL3KD} N= 13023 cells, VUB14^{del18q} N= 27466 cells, VUB14^{del18q_SALL3OE} N= 49709 cells, VUB13^{del18q} N= 39424 cells, VUB13^{del18q_SALL3OE} N= 48155 cells.
**Figure 5, Cardiac progenitors**	Each datapoint shown in 5C represents the average of the quantification of 5 randomly taken images of the same well: VUB04^{WT-NT} N= 15015 cells, VUB04^{WT_SALL3KD} N= 14997 cells, VUB03^{WT-NT} N= 23719 cells, VUB03^{WT_SALL3KD} N= 13114 cells, VUB02^{WT-NT} N= 14659 cells, VUB02^{WT_SALL3KD} N= 12089 cells, VUB14^{del18q} N= 23652 cells, VUB14^{del18q_SALL3OE} N= 23809 cells, VUB13^{del18q} N= 22286 cells, VUB13^{del18q_SALL3OE} N= 17318 cells.
**Figure 6, Hepatoblasts**	Each datapoint shown in 6C represents the average of the quantification of 5 randomly taken images of the same well: VUB04^{WT-NT} N= 15703 cells, VUB04^{WT_SALL3KD} N= 12316 cells, VUB03^{WT-NT} N= 28766 cells, VUB03^{WT_SALL3KD} N= 17809 cells, VUB02^{WT-NT} N= 20361 cells, VUB02^{WT_SALL3KD} N= 17414 cells, VUB14^{del18q} N= 22061 cells, VUB14^{del18q_SALL3OE} N= 18296 cells, VUB13^{del18q} N= 17302 cells, VUB13^{del18q_SALL3OE} N= 16282 cells.

Supplementary table 7

Primary Antibodies

Gene	Species	Supplier	Catalog number	Dilution
PAX6	Mouse	Abcam	ab78545	1:200
HNF4A	Mouse	Santa Cruz Biotechnology	Sc-374229	1:200
GATA4	Rabbit	Cell Signaling Technology	36966S	1:200
POU5F1	Rabbit	Cell Signaling Technology	2840S	1:400
POU5F1	Mouse	Santa Cruz Biotechnology	sc-5279	1:200

Secondary Antibodies

Gene	Conjugate	Supplier	Catalog number	Dilution
Donkey anti Mouse	Alexa Fluor 488	Thermo Fischer Scientific	A-21202	1:200
Donkey anti Mouse	Alexa Fluor 594	Thermo Fischer Scientific	A-21203	1:200
Donkey anti Rabbit	Alexa Fluor 488	Thermo Fischer Scientific	A-21206	1:200
Donkey anti Rabbit	Alexa Fluor 594	Thermo Fischer Scientific	A-21207	1:200
Hoechst 33342		Invitrogen	H3570	1:2000
